# Phenotypic high-throughput screening identifies modulators of gut microbial choline metabolism

**DOI:** 10.1128/mbio.01172-25

**Published:** 2026-02-23

**Authors:** Amelia Y. M. Woo, Walter J. Sandoval-Espinola, Maud Bollenbach, Alison Wong, Mariko Sakanaka-Yokoyama, Qijun Zhang, Vincent Nieto, Federico E. Rey, Emily P. Balskus

**Affiliations:** 1 Department of Chemistry and Chemical Biology, Harvard University 1812, Cambridge, Massachusetts, USA; 2 Department of Bacteriology, University of Wisconsin-Madison 5228, Madison, Wisconsin, USA; 3 Department of Medical Microbiology and Immunology, University of Wisconsin-Madison 5228, Madison, Wisconsin, USA; 4 Howard Hughes Medical Institute, Havard University 2405, Cambridge, Massachusetts, USA; Ludwig-Maximilians-Universitat Munchen, Munich, Germany

**Keywords:** gut microbiome, small molecule inhibitors, phenotypic screening, anaerobic choline metabolism, TMA, TMAO, gnotobiotic

## Abstract

Anaerobic metabolism of dietary choline to trimethylamine (TMA) by the human gut microbiome is a disease-associated pathway. The host’s impaired ability to oxidize TMA to trimethylamine-*N*-oxide (TMAO) results in trimethylaminuria (TMAU), while elevated serum TMAO levels have been positively correlated with cardiometabolic disease. Small molecule inhibition of gut bacterial choline metabolism attenuates the development of disease in mice, highlighting the therapeutic potential of modulating this metabolism. Inhibitors previously developed to target this pathway are often designed to mimic choline, the substrate of the key TMA-generating enzyme choline trimethylamine-lyase (CutC). Here, we use a growth-based phenotypic high-throughput screen and medicinal chemistry to identify distinct chemical scaffolds that can modulate anaerobic microbial choline metabolism and lower TMAO levels *in vivo*. These results illustrate the potential of using phenotypic screening to rapidly discover new inhibitors of gut microbial metabolic activities.

**IMPORTANCE:**

Gut microbial metabolic activities play important roles in human health, prompting interest in the discovery of gut microbiome-targeted small molecule inhibitors as potential therapeutics. Anaerobic choline metabolism by the gut microbiome generates trimethylamine and its downstream metabolite trimethylamine-*N*-oxide (TMAO), which cause trimethylaminuria and are correlated with cardiometabolic diseases, respectively. Current strategies for modulating microbial metabolism with small molecule inhibitors typically require having a target enzyme. Here, we show that a growth-based phenotypic screen can identify inhibitors of choline metabolism with chemical scaffolds that are structurally distinct from choline and existing inhibitors. The resulting optimized compounds lower serum TMAO in gnotobiotic mice without significantly perturbing gut microbiome composition. This work highlights the potential of using phenotypic screening to rapidly discover additional inhibitors of microbial metabolic activities, which would accelerate mechanistic studies of the microbiome and deepen our understanding of disease biology from correlation to causation.

## INTRODUCTION

The human gut microbiome has been increasingly associated with host physiology, and this has prompted the development and application of various approaches to manipulate the composition and functions of this microbial community (1). These strategies include fecal microbiome transplant (FMT), use of antibiotics, prebiotics, wild-type or genetically modified probiotics, and phage therapy (2, 3). Small molecule inhibitors that confer precise control over specific metabolic activities broadly across multiple gut microbial species, without exhibiting broad-spectrum antimicrobial activity, are ideal not just for therapeutic applications but also for basic studies of the gut microbiome (4, 5). Changes in host phenotypes induced by treatment with gut microbiome-targeted small molecule inhibitors in animal models could be used to infer the causality of host–microbiome associations and could provide critical support for microbiome-targeted therapeutic interventions (6).

Efforts to develop small molecule inhibitors as tools to study the gut microbiome have accelerated over the last decade, beginning with the seminal example of bacterial β-glucuronidase inhibitors (7–11). Other key examples include inhibitors of choline trimethylamine-lyase (CutC) (12–15), amino acid decarboxylases (16, 17), bile salt hydrolases (18, 19), and tryptophanases (20). However, inhibitor discovery in this space has primarily relied on having a biochemically well-characterized target for inhibitor development. This sharply contrasts with antibiotic (21), anti-virulence (22–24), and anti-infective (25–27) drug discovery, in which target-agnostic, phenotypic screening campaigns are common.

While underutilized in the context of the gut microbiome, phenotypic screening offers several advantages over target-based inhibitor discovery strategies (28). First, the requirement for compounds to access their targets in a cellular setting is built into the screen. This may be especially important when targeting metabolic activities that are widespread across bacterial phyla, as differences in cell membrane architectures can significantly influence the permeability of small molecules (29). Secondly, such screens can be performed with more complex microbial communities and gut microbiome samples (30), which may provide results that are more physiologically relevant, such as in the case of phenotypic screens in organoids (31). Lastly, phenotypic screens are target-agnostic and therefore provide an opportunity to both discover new biologically relevant targets and shed light on previously unappreciated aspects of microbial function.

**Fig 1:**
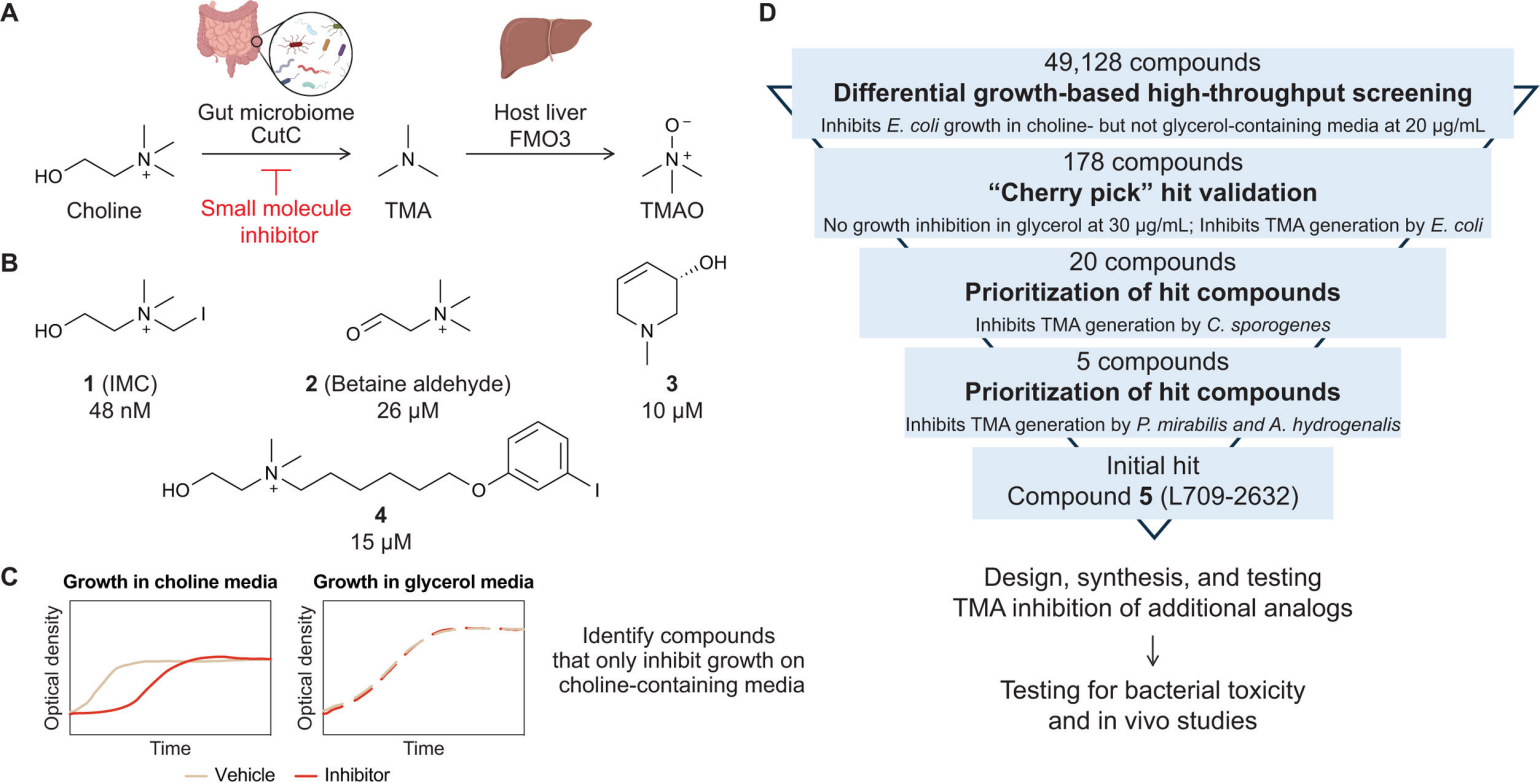
Small molecule inhibitors of microbial choline metabolism can be identified through phenotypic screening. (**A**) Dietary choline is metabolized by the gut microbial enzyme CutC into TMA, which is further oxidized to TMAO by host FMO3 enzymes in the liver. Both TMA and TMAO are disease-associated metabolites, and generation of these metabolites can potentially be reduced by targeting gut microbial choline metabolism with small molecule inhibitors. (**B**) Chemical structures of selected reported inhibitors of anaerobic choline metabolism and their IC_50_ values against bacterial cultures. (**C**) Small molecule inhibitors of choline metabolism can be identified by comparing bacterial growth on minimal media supplemented with choline or an alternate carbon and energy source. (**D**) Overall inhibitor development workflow began with identifying inhibitors using high-throughput phenotypic screening for growth inhibition of *E. coli* MS 200-1 followed by validation and prioritization of compounds based on their EC_50_ for inhibition of TMA production from choline across multiple choline-metabolizing bacteria. New analogs were designed, synthesized, and iteratively tested for their TMA inhibitory activity, and promising compounds were evaluated for their bacterial toxicity and *in vivo* activity.

One disease-associated gut metabolic activity of interest is the anaerobic catabolism of choline to trimethylamine (TMA) (32–34). TMA is an exclusively microbial metabolite in the body, with choline as its major precursor (32, 35). TMA generated by the gut microbiome is typically oxidized in the liver by flavin monooxygenase 3 (FMO3) enzymes to form trimethylamine-*N*-oxide (TMAO), which is then excreted (36) (Fig. 1A). However, individuals who have an impaired ability to oxidize TMA excrete this malodorous metabolite and suffer from a socially debilitating condition called trimethylaminuria (TMAU) (37). Multiple studies have also found that high levels of circulating serum TMAO in humans strongly correlate with the incidence of cardiometabolic diseases, such as cardiovascular disease (38–40), chronic kidney disease (41, 42), type II diabetes (43), and non-alcoholic fatty liver disease (44). Mice that have been fed with high choline or high TMAO diets develop symptoms associated with cardiovascular disease and have more severe heart failure (45), and this pathway has also been shown to play a causal role in atherosclerosis development in ApoE knockout mice (38). Moreover, the metabolism of choline by gut microbes can also reduce availability of this essential nutrient for the host (46). Due to the prevalence of diseases linked to TMA, there is strong interest in developing inhibitors of gut microbial choline metabolism as potential therapeutics. Besides the opportunity for drug development, small molecules that can selectively inhibit anaerobic choline metabolism in complex gut microbiomes would be useful tools for understanding this metabolic activity, as there remain questions regarding how dietary choline impacts chronic plasma TMAO levels (47).

Anaerobic choline metabolism is widespread across bacterial phyla, and this activity has been observed in both gram-positive and gram-negative gut bacteria (48). This metabolism requires enzymes encoded within the choline utilization (*cut*) gene cluster (49), including the glycyl radical enzyme CutC, which performs the chemically challenging C–N bond cleavage of choline to generate TMA and acetaldehyde (50). Bacteria that encode and express the *cut* gene cluster metabolize the acetaldehyde byproduct into ethanol and acetyl-CoA, enabling energy production, while TMA is excreted. As such, CutC has been the canonical target for the development of inhibitors of anaerobic choline metabolism.

Structural mimics of choline such as halomethylcholines (13), betaine aldehyde (**2**) (14), and cyclic choline analogs (**3**) (15), have been identified based on the structure of CutC and its proposed mechanism (50, 51) and have shown to be potent inhibitors of anaerobic choline metabolism both *in vitro* and *in vivo* (13, 52–54) (Fig. 1B). Iodomethylcholine (**1**, IMC), one of the most potent CutC inhibitors, has a half-maximal inhibitory concentration (IC_50_) of 1.5 nM against *Proteus mirabilis* lysates (13). In animal models of disease, IMC has also been shown to reduce renal tubulointerstitial fibrosis (52), reduce markers of renal injury (53), and improve cardiac function (54). When dosed at 100 mg/kg, IMC is found at around 6 mM in mice cecum and colon samples and is thus largely retained in the gut after 24 h (13). However, the inhibitor and its metabolite, iodomethylbetaine, can be detected at low micromolar levels in serum, peaking at 2 h post-gavage (13). IMC has previously been shown to be a potent inhibitor of choline transport in rat synaptosomes, with an IC_50_ of 0.49 µM, and is thought to bind irreversibly to the high affinity choline transporter (HAChT) (55), which raises concerns of potential neurotoxicity and motivates the discovery of additional inhibitors.

To our knowledge, there has only been one prior attempt to identify inhibitors of anaerobic choline metabolism using phenotypic screening. A matrix-assisted laser desorption/ionization time-of-flight (MALDI–TOF) mass spectrometry-based phenotypic screen measured derivatized TMA in bacterial cultures and identified an inhibitor of a human choline transporter (**4**) as a modulator of anaerobic choline metabolism, with an IC_50_ of 15 µM in *Hungatella hathewayi* cultures (56). This compound inhibits choline transport in human cells that overexpress choline transporters with an IC_50_ of 44 nM (57).

Given the structural similarity of reported inhibitors to choline and their potential ability to interact with host choline receptors and transporters, we sought to identify inhibitors of microbial choline metabolism with more drug-like scaffolds. Here, we identify novel, broad-spectrum inhibitors of anaerobic microbial choline metabolism using a target-agnostic differential growth-based high-throughput screen (HTS). Our inhibitor optimization efforts reveal that a cyclic amine is essential for activity. These compounds are structurally distinct from previously reported CutC inhibitors. They are non-lethal to both gram-positive and gram-negative commensal bacteria and have improved broad-spectrum activity compared to our initial hit. Finally, we demonstrate that these inhibitors can lower TMAO levels in gnotobiotic and conventional mouse models. We anticipate that this type of growth-based HTS screening workflow can be readily adapted to identify compounds that modulate additional gut bacterial metabolic activities, including those with no known associated enzyme targets.

## RESULTS

### Differential growth as a phenotype for high-throughput screening

To identify inhibitors of choline metabolism in a target-agnostic manner, we envisioned using a phenotypic HTS with choline-metabolizing bacteria. The previous phenotypic screen for choline metabolism inhibitors used high-throughput quantitation of derivatized TMA by MALDI–TOF mass spectrometry to screen a library of 10,229 compounds (56). While this method successfully identified compounds that inhibit the transformation of choline to TMA, this approach has several disadvantages, including a requirement for specialized instrumentation, the need for chemical derivatization, and an analysis time that scales considerably with library size. Microbial growth is a more straightforward readout for screening, as optical density of cultures can be measured quickly with a plate reader in a high-throughput fashion. This type of screen is routinely used for antibiotic discovery but is rarely used to identify inhibitors of specific microbial metabolic activities because many functions of interest are not readily linked to bacterial growth (58, 59).

Inspired by our previous data showing that a rationally designed CutC inhibitor could inhibit bacterial growth on choline (14), we conceived of an alternative phenotypic screening approach. Anaerobic choline metabolism is a non-essential pathway when alternate carbon and energy sources are available. However, it can become essential when choline is the sole carbon and energy source during anaerobic growth (46). Thus, choline metabolism can be linked directly to a growth phenotype, enabling a HTS. To identify and remove broad-spectrum bacterial growth inhibitors from consideration, such as antibiotics and antimicrobials, we envisioned performing a counter-screen using the same minimal medium containing an alternative carbon and energy source. In this format, compounds that specifically interfere with anaerobic choline metabolism should inhibit bacterial growth in choline-containing medium but not in the alternate medium (Fig. 1C). This differential growth-based screening strategy is straightforward to implement, making it easily scalable to screen large compound libraries and readily extendable to other organisms and metabolic activities.

We chose to use the choline-metabolizing strain *Escherichia coli* MS 200-1 for the HTS as it is a human gut isolate and can be grown anaerobically at 37°C in no carbon essential (NCE) medium supplemented with choline (46). This medium contains fumarate as the terminal electron acceptor and can be supplemented with other carbon sources. Using kanamycin and DMSO as the positive and negative controls, respectively, we optimized the assay in a 384-well plate format to have Z’ values > 0.5, making it suitable for the HTS (60). We chose to measure optical densities of the cultures at 7 h and 20 h after inoculation to identify partial (decreased growth rate) or total growth inhibitors in choline-containing media. A simultaneous counter-screen with glycerol-containing NCE medium would also be performed. Thus, compounds that inhibited *E. coli*’s growth on choline but not glycerol NCE media would be considered potential hits.

We then planned to validate the activity of potential hits toward *E. coli* MS 200-1 by confirming that they had no growth-inhibitory effects in NCE glycerol medium and by determining their TMA inhibitory activity. Half-maximal effective concentration (EC_50_) values of TMA inhibition were calculated after measurement using a liquid chromatography–mass spectrometry (LC–MS/MS)-based assay. We planned to further prioritize compounds to test against additional choline-metabolizing gut isolates based on their EC_50_ values. Direct measurement of TMA inhibition would be a key step in our HTS efforts because the growth-based screen was not designed to differentiate compounds that specifically inhibit TMA generation from compounds that may inhibit the downstream metabolism of acetaldehyde, which is also linked to growth. Scaffolds identified through this screening would be further optimized for their potency for TMA inhibition using medicinal chemistry. Finally, the most potent analogs would be evaluated for their toxicity against a panel of gut bacterial isolates and their ability to lower TMAO levels *in vivo* (Fig. 1D).

### Growth-based phenotypic HTS identifies new inhibitor scaffolds

The primary screen was performed in duplicate using the ChemDiv7 commercial library from the Institute of Chemistry and Cell Biology–Longwood (ICCB-L) Screening Facility, consisting of 49,128 compounds (Fig. 1D). Each compound was tested at a concentration of 20 µg/mL. As described above, compounds that differentially inhibited *E. coli* MS 200-1 growth on choline (Z-score < –2) but not glycerol (Z-score ≥ 0) media at either the 7 h or 20 h time points were considered potential hits (Fig. S1).

**Fig 2:**
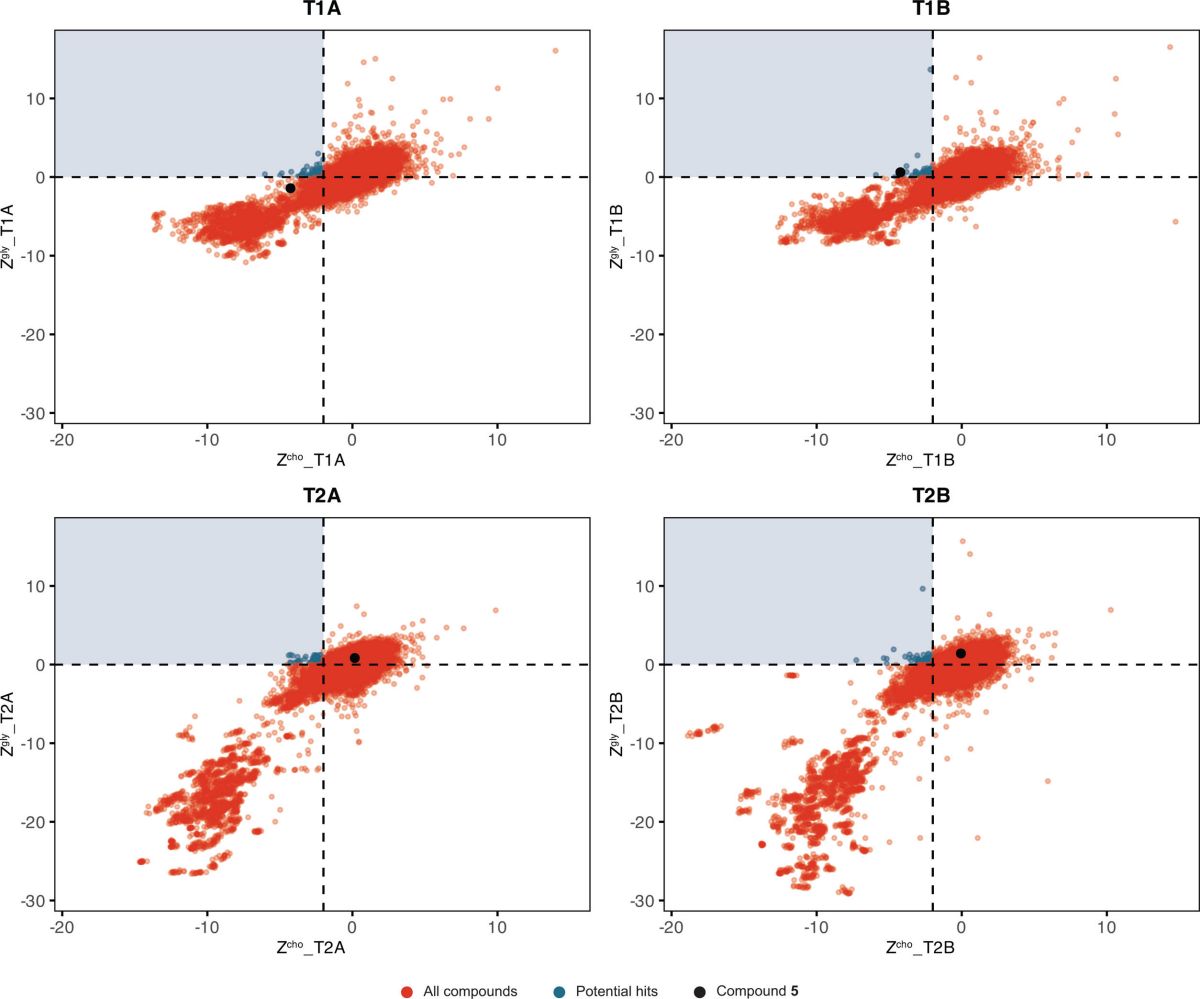
Differential growth-based phenotypic screening identified inhibitors of anaerobic gut bacterial choline metabolism. Scatter plots of both replicate plates comparing Z-scores of compounds in choline medium versus glycerol medium at 7 h (T1) and 20 h (T2). Compounds that met the criteria on either replicate plate were chosen for retesting and are colored in blue. Hit compound **5** is labeled and colored in black in all plots. Positive and negative control wells with kanamycin and DMSO vehicle were not given Z-scores.

The HTS identified 60 compounds that differentially inhibited growth on choline at 7 h and 122 compounds that differentially inhibited growth at 20 h on either replicate plate (Fig. 2). Of these, 4 compounds differentially inhibited growth at both 7 h and 20 h, resulting in a total of 178 potential hits at either time point. We next performed a “cherry pick” screen (i.e., hit validation) with 178 compounds, or 0.362% of the ChemDiv7 library. Comparatively, when screening a smaller library of 10,229 compounds, the MALDI–TOF method had a higher hit rate of 2.5% (56), suggesting that this growth-based screening strategy is not only easier to scale but also may afford fewer false positives.

To validate the potential hits from the HTS, we tested the 178 compounds for inhibition of growth of *E. coli* MS 200-1 in glycerol minimal media at 30 µg/mL, following up with those without activity. As the number of compounds re-evaluated was significantly fewer than the primary screen, we defined growth inhibition as cultures that had an optical density below 5% standard deviation from the mean optical density of the plate, rather than using Z-scores. We then determined EC_50_ values for inhibition of TMA production from choline by bacterial cultures incubated with [trimethyl-*d*_9_]choline in phosphate-buffered saline. Of the 178 compounds tested in both growth and TMA inhibition assays, 20 inhibited TMA generation by *E. coli* MS 200-1 without growth-inhibitory effects in glycerol minimal medium.

We then used secondary assays to prioritize scaffolds that had broad-spectrum TMA inhibitory activity against additional choline-metabolizing gut bacteria. For this, we used *Clostridium sporogenes* ATCC 15579, a human gut isolate that is able to efficiently ferment choline in addition to other carbon sources, as well as *Anaerococcus hydrogenalis* DSM 7454 and *Proteus mirabilis* ATCC 29906, human gut isolates previously identified to metabolize choline *in vitro* (61). In particular, *E. coli* MS 200-1 and *C. sporogenes* ATCC 15579 have been used in gnotobiotic studies to investigate the effects of bacterial choline metabolism on the host and microbiome (46, 61, 62). These strains are representative of taxa frequently detected in humans (63) and, together with *E. coli*, are representative of the major phyla and genera in which TMA producers have been detected across human gut isolates (48). Importantly, this group of strains included both gram-positive and gram-negative bacteria. Gram-negative bacteria have an additional outer membrane layer and associated efflux pumps (29), which typically decreases small molecule permeability compared to gram-positive bacteria. It also included bacteria with *cut* gene cluster architectures that differ in the number and types of microcompartment proteins encoded and in the size of the CutC enzyme (48). We hypothesized that testing the inhibitors against multiple choline-utilizing gut bacteria would increase the likelihood of identifying compounds with broad-spectrum activity.

**Fig 3:**
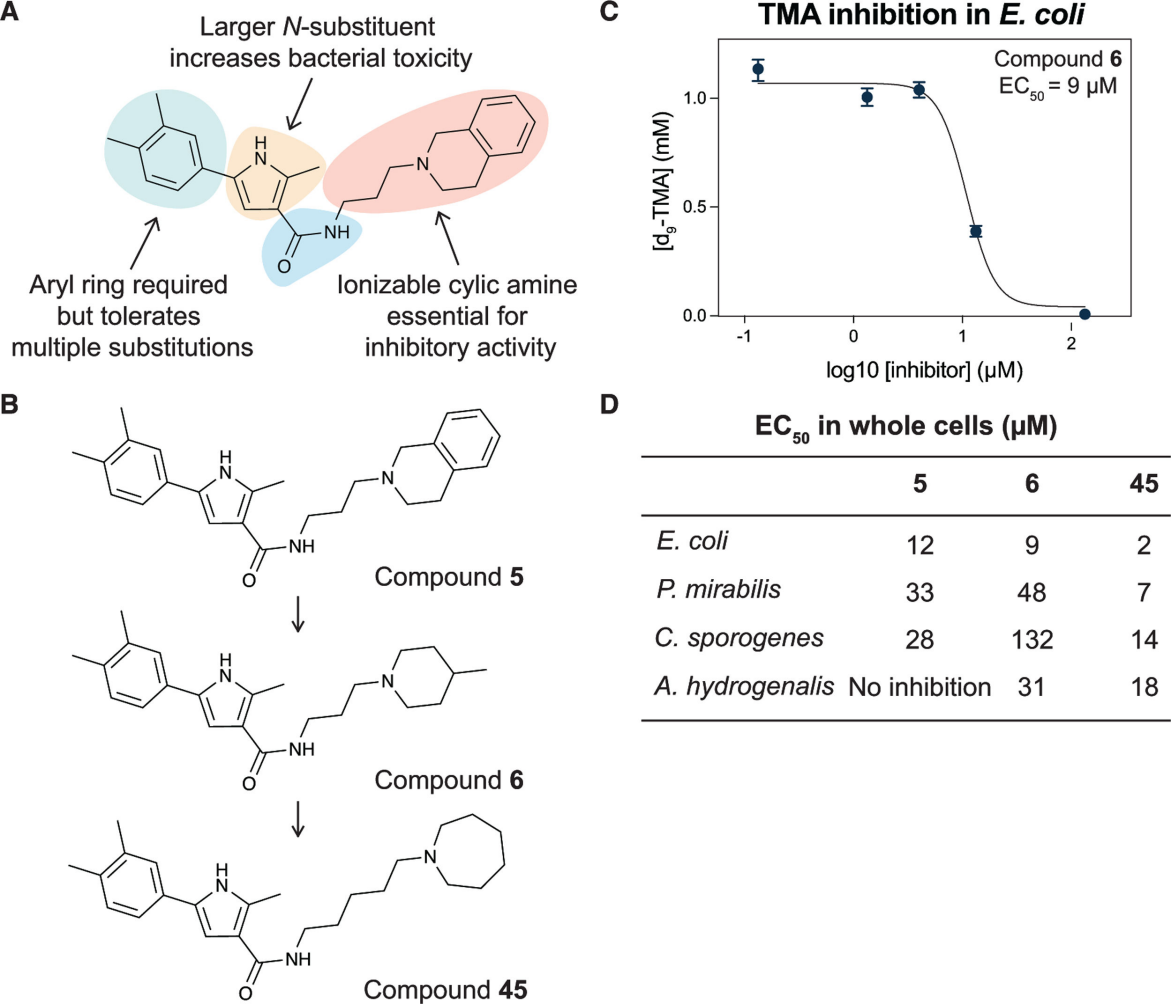
Iterative design, synthesis, and testing of analogs resulted in improved inhibitor **45**. (**A**) Summary of structure–activity relationships around each component of compound **5**. (**B**) Chemical structures of initial hit compound **5**, subsequent analog **6**, and most potent lead compound **45**. (**C**) TMA inhibitory activity (EC_50_ in µM) of compound **6** against *E. coli* MS 200-1. (**D**) TMA inhibitory activity (EC_50_ in µM) of compounds **5**, **6**, and **45** against the panel of four choline-metabolizing gut bacteria.

This series of secondary assays identified five heterocyclic compounds that inhibited TMA production across multiple bacterial strains (Table 1), of which compound **5** emerged as a promising lead for medicinal chemistry efforts. This compound displayed the best potency across three of the four choline metabolizers, with EC_50_ values for TMA inhibition ranging from 12 to 33 µM, and it had no impact on growth of *E. coli* MS 200-1 in glycerol medium. Compound **5** has a pyrrole-3-carboxamide as its core scaffold, with a phenyl group at C5 of the pyrrole and an amine linked through the carboxamide, all of which were investigated for their effects on TMA inhibitory activity (Fig. 3A). Compound **5** was an attractive starting point for our medicinal chemistry efforts, as it was more drug-like than existing inhibitors and its analogs were synthetically accessible. Testing commercially available analogs of **5** identified inhibitor **6**, which was active across all four choline-metabolizers tested (Fig. 3B through D). Compound **6** had an EC_50_ of 9 µM against *E. coli* in our LC–MS/MS-based TMA inhibition assay and was subsequently used as our reference compound when comparing inhibition of TMA generation in downstream EC_50_ assays across additional analogs.

**TABLE 1:** Z-scores and EC50 values of the top 5 hits identified from HTS and secondary screening

Compound	Z-scores from HTS				EC_50_ (µM) in whole-cell assays			
	Z^cho^ at T1	Z^gly^ at T1	Z^cho^ at T2	Z^gly^ at T2	*E. coli*	*P. mirabilis*	*C. sporogenes*	*A. hydrogenalis*
L709-2632 (compound 5)	−4.27–4.23	−1.420.61	0.16–0.06	0.831.41	12	33	28	No inhibition
G789-2247	−2.76–3.09	−0.820.62	0.630.33	0.231.46	29	59	31	
F154-0585	−2.13–1.18	0.810.71	0.290.61	1.24–0.39	31	100	31	No inhibition
G856-6017	−2.41–2.89	0.02–0.33	−0.7–0.05	−0.76–1.1	43	No inhibition	15	
G781-1263	−0.93–2.05	−2.090.48	−1.77–2.53	−2.11–1.65	74	73	137	No inhibition

### Medicinal chemistry effort improves inhibitor potency and broad-spectrum activity

We next sought to improve the potency of inhibitor **6** in reducing TMA generation from choline across various choline-metabolizing gut bacteria, while minimizing its effects on the growth of other gut bacteria. Thus, we generated and evaluated analogs of **6** for their TMA inhibitory activity and gut bacterial growth inhibition. To guide these efforts, we tested the analogs’ ability to inhibit the generation of TMA from choline by the four choline-metabolizing gut bacteria described above. We prioritized compounds that maintained activity across at least one gram-positive and one gram-negative strain for further testing. We next tested the effects of prioritized compounds on the anaerobic growth of nine gut commensal bacteria in complex media, including the four choline-metabolizers and five non-choline-metabolizing organisms (*Bacteroides caccae* ATCC 43185, *Bacteroides ovatus* ATCC 8483, *Bacteroides thetaiotaomicron* VPI-5482, *Collinsella aerofaciens* ATCC 25986, and *Eubacterium rectale* ATCC 33656). These additional species are prevalent human gut bacteria and have been used in prior gnotobiotic mouse studies of choline metabolism (61). We prioritized compounds that had minimum inhibitory concentrations (MICs) ≥200 µM across all strains, i.e., 10-fold higher than their EC_50_s for inhibition of TMA generation by choline-metabolizing bacteria.

We first investigated the importance of the heterocyclic core of compound **6** (see Table S1). Removing the C2 methyl group on the pyrrole (**7**) reduced potency by about 2-fold across all four choline-metabolizing bacteria, while moving the methyl group to the C4 position (**8**) increased the EC_50_ in *E. coli* by more than 10-fold. Changing the C2 methyl substituent to an ethyl group (**9**) improved activity slightly in *C. sporogenes* but reduced activity in *E. coli*. We then changed the relative positions of the aryl and carboxamide on the pyrrole. Keeping the aryl substituent at C5 and moving the carboxamide from C3 (**7**) to C2 (**10**) largely decreased activity in *E. coli* and *C. sporogenes* while slightly improving activity in *P. mirabilis*. Keeping the carboxamide on C3 and moving the aryl substituent from C5 (**7**) to C4 (**11**) similarly decreased activity in *C. sporogenes* while improving activity in *P. mirabilis*. Replacing the pyrrole core (**6**) with a furan (**12**) decreased activity across bacteria except for *C. sporogenes*. Comparatively, replacing the pyrrole core (**7**) with thiophene (**13**) maintained activity in *E. coli* and *P. mirabilis* and improved activity in *C. sporogenes*, while a thiazole (**14**) scaffold decreased activity across all bacteria tested.

Keeping the pyrrole scaffold, we then introduced larger substituents on the pyrrole nitrogen (see Table S2). Methylating the pyrrole nitrogen (**15**) improved activity in *C. sporogenes* compared to the non-methylated counterpart (**7**) but reduced activity across the rest of the bacteria panel. Analogs with larger *N*–substituents (**16**–**19**) were found to be at least 10-fold more active against *C. sporogenes* but were also less active towards *E. coli*. We next explored modifying the aryl group at the C5 position (see Table S3). We found this substituent was critical, as removal of this group (**20**) greatly reduced activity across all bacteria. Replacement of the aryl group with cyclohexane (**21**) improved potency in *C. sporogenes* but reduced potency in *E. coli*. Removal of the 3,4-dimethyl substituents (**22**) or the 4-methyl substituent (**23**) on the aryl group decreased activity against *P. mirabilis* while generally retaining activity across other bacteria, whereas removal of the 3-methyl substituent (**24**) greatly reduced activity in *E. coli*. Moving the 4-methyl substituent to the 5-position (**25**) retained activity across all bacteria. However, replacing the aryl group with a 2-methylpyridin-4-yl substituent (**26**) abolished activity. We then examined the necessity of the carboxamide linkage at the C3 position and found that the methylated amide (**27**) and ester (**28**) derivatives were less active in *E. coli* than the original hit (see Table S4).

Lastly, we studied the importance of the cyclic amine linked to the carboxamide (Table 2). Varying the linker length between 2 and 5 carbons (**29**–**31**) showed that the longest linker length improved potency in *C. sporogenes* and retained activity in *E. coli*, *P. mirabilis*, and *A. hydrogenalis*, while shortening the linker was detrimental in all organisms except *E. coli*. Compared to compound **29**, replacement of the cyclic amine with a cyclohexene (**32**) or ether (**33**) abolished activity. When the cyclic amine in **29** is replaced with a primary (**34**), secondary (**35**), or acyclic tertiary amine (**36**), the activity across bacteria is greatly reduced. Replacing the 4-methylpiperidine of **6** with a morpholine (**37**) or 4-methyl-3-oxopiperazine (**39**) largely decreased activity, while the 4-methylpiperazine (**38**) analog lost activity in *A. hydrogenalis*. Varying the ring size of the cyclic amine of **6** (**40**–**43**) identified eight-membered ring analog **43** with generally improved activity across bacteria. Combining an increase in the ring size from piperidine to azepane with an increased linker length (**44, 45**) improved potencies across bacteria and led to the most promising derivative (**45**), which is 2–10-fold more potent across the panel of bacteria than **6**. Compared to compound **6**, the most active compound, **45**, contains an azepane instead of the 4-methylpiperidine and has a linker length of five carbons. Compound **45** has improved EC_50_ values against all bacteria tested compared to **6**, with a marked improvement in activity in *C. sporogenes*.

**TABLE 2:** EC_50_ values of compounds **6** and **29**–**45** with varying amine substituents

Compound	EC_50_ in whole cells (µM)			
	*E. coli*	*P. mirabilis*	*C. sporogenes*	*A. hydrogenalis*
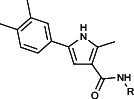				
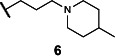	9	48	132	31
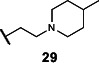	7	64	213	176
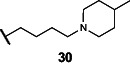	5	35	138	27
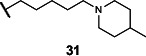	11	66	84	33
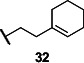	>100	N/A^*a*^	No inhibition at 100	N/A
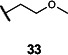	No inhibition at 100	N/A	No inhibition at 100	N/A
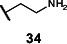	140	No inhibition at 312	No inhibition at 312	N/A
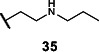	60	>312	>312	N/A
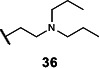	200	>312	>312	N/A
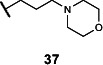	>125	No inhibition at 312	No inhibition at 312	N/A
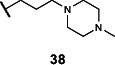	14	58	92	No inhibition at 312
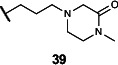	>125	No inhibition at 312	>312	N/A
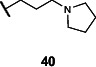	11	104	78	83
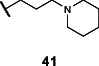	10	193	267	244
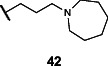	2	40	104	43
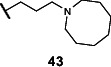	3	28	40	28
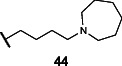	8	33	68	7
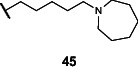	2	7	14	18

^*a*^ N/A indicates that compound was not tested against bacterial strain.

To ensure that the TMA inhibition observed was not due to antibacterial activity, we tested select compounds for their growth inhibition of commensal bacteria (see Table S5). While most analogs did not display antibacterial activity up to 200 µM, an analog with a benzyl substituent on the pyrrole nitrogen (**19**) inhibited growth (MIC < 200 µM) in five out of 9 bacterial strains tested. While the potent TMA inhibitory activity of this compound against *C. sporogenes* was not likely due to antibacterial activity, this highlighted the need to validate potent compounds in the MIC assay to ensure that any reduction in choline metabolism was not due to growth inhibition and broad toxicity.

Overall, through our medicinal chemistry efforts, we found that the cyclic amine was crucial for TMA inhibitory activity. Larger *N*–substituents on the pyrrole scaffold increased bacterial toxicity, while the aryl ring generally tolerated various substitutions. Interestingly, we observed that the activity of the inhibitors did not correlate with whether the target bacteria were gram-positive or negative. In general, the compounds tested were most active against *E. coli* and *A. hydrogenalis* and less active against *C. sporogenes* and *P. mirabilis*. Most compounds tested were non-toxic to commensal bacteria (MICs >200 µM across all strains) with the notable exception of analogs with larger *N*-substitutions on the pyrrole. Our medicinal chemistry efforts highlight the importance of testing both compound efficacy and antibacterial activity across multiple bacteria.

### Preliminary mode of action assays suggest a complex mode of action for inhibitors

In addition to the key TMA-forming enzymes CutC and CutD, previous genetic studies in *cut*^+^ *E. coli* 536 identified transcriptional regulators and putative transporters encoded in the *cut* gene cluster as important for TMA generation (64). To gain preliminary insights into potential modes of action of our inhibitors, we assayed their effects on the activity of CutC/D *in vitro*, the transcription of the *cut* gene cluster, and the transport of choline into bacteria.

To investigate if our compounds were inhibiting CutC directly, we tested their activity toward the purified enzyme. The activity of CutC was determined using a coupled enzyme assay, where the acetaldehyde product from the cleavage of choline to TMA is reduced to ethanol by yeast alcohol dehydrogenase (YADH) using nicotinamide adenine dinucleotide (NADH) as a cofactor. The consumption of NADH results in a change in absorption at 340 nm, and this rate of change of absorption was used to calculate the initial rates of reaction of CutC in the presence of varying concentrations of inhibitors (14, 65).

We found that our analogs had varying inhibitory effects on CutC *in vitro*. While most compounds tested had some activity *in vitro*, this did not always correlate with their TMA inhibitory activity in whole cells (Table 3). For example, compounds **20** and **37** displayed low or no inhibition in both whole cell and enzyme assays, and compounds **6**, **42**, and **45** showed improved activity in whole cells and improved activity toward the purified enzyme. However, compounds **23** and **28** that were potent in whole cells showed lower or no activity toward CutC, and compounds **26** and **27,** which were not active in whole cells, inhibited CutC. This suggested that while some compounds may exert their TMA inhibitory effects in part through CutC, the analogs may display different cell permeability, have different access to CutC/D in the microcompartments found in whole cells, or have different toxicities. Because several compounds active in whole cells do not inhibit CutC *in vitro*, this suggests that there are other cellular targets beyond CutC that are important for inhibitory activity. As we were unable to reconstitute CutC/D encapsulated in the microcompartments *in vitro* for testing, we are hesitant to conclude that CutC is the direct target of our compounds.

**TABLE 3:** Comparison of whole-cell EC50 values in *E. coli* and purified CutC IC50 values of select compounds

Compound	Whole cell EC_50_ in *E. coli* (µM)	Purified CutC IC_50_ (µM)
6	9	48
20	>125	>125
23	5	64
26	>125	45
27	>125	18
28	15	No inhibition
37	>125	No inhibition
42	2	24
45	2	17

To evaluate the impacts of our compounds on *cut* gene cluster expression, we leveraged the observation that expression is induced by choline in growth media. We cultured *E. coli* in NCE glycerol, a defined medium without choline. Either vehicle (DMSO) or 10 µM of compound **42** was included in the growth medium. We added choline at mid-log phase and measured transcription of the *cut* genes using a Nanostring nCounter. We verified that transcription of the *cut* gene cluster was induced by choline addition and found that compound **42** did not significantly change gene expression as compared to the DMSO control (Fig. S2A). This result implies that repression of the *cut* gene transcription is not likely a mechanism by which compound **42** inhibits TMA generation.

To test if our compounds inhibited choline transport into bacterial cells, we incubated ∆*cutC E. coli* MS 200-1 cells with [trimethyl-^14^C_3_]choline and measured the uptake of radioactivity. The ∆*cutC* strain of *E. coli* was used in this assay to prevent the formation of volatile radioactive TMA. When cells were grown aerobically, incubation with compound **42** increased choline uptake by 15 times compared to vehicle treatment (Fig. S2B). Similarly, when cells were grown anaerobically, incubation with inhibitor **42** also increased choline uptake, though only 1.2 times compared to vehicle. These preliminary results suggest that compound **42** does not inhibit choline transport.

### Inhibitor 45 lowers serum TMAO in gnotobiotic and conventional mice

We then evaluated a promising inhibitor in a gnotobiotic mouse model. We ranked the compounds based on their potency in *E. coli* whole cells and lack of toxicity against the gut commensal panel. We chose to test inhibitor **45** as it was one of the most potent compounds across the choline-metabolizing bacteria (EC_50_ values between 2 and 18 µM) while remaining non-toxic to other commensals (MICs >200 µM). In our experiment, we used germ-free C57BL/6 mice colonized with a previously described defined microbial community consisting of five non-choline-metabolizing strains from diverse phylogenetic groups and *E. coli* MS 200-1 as the sole choline-metabolizing strain. This gnotobiotic model has been used to investigate the impact of gut microbial choline metabolism on host physiology (46). Since this *E. coli* strain is susceptible to our inhibitor *in vitro* and is the only choline-metabolizing strain in this community, any reduction in TMA generation *in vivo* can be attributed to the activity of inhibitor **45** *in vivo*.

**Fig 4:**
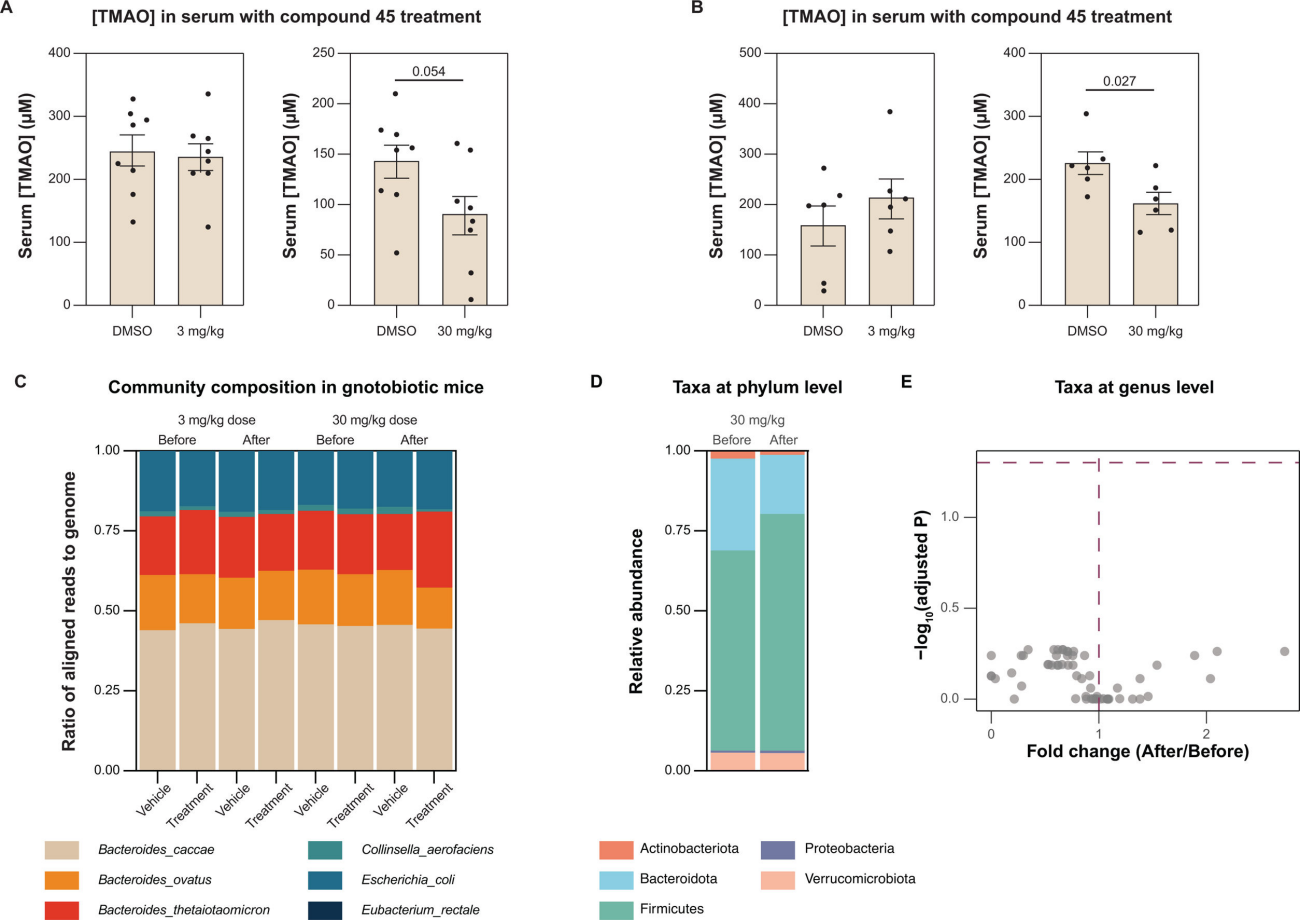
Treatment with inhibitor **45** decreased serum TMAO in gnotobiotic and conventional mice without significant changes in gut microbiome composition. (**A**) Serum TMAO levels in gnotobiotic mice after inhibitor **45** treatment. TMAO levels decreased by ~40% when dosed at 30 mg/kg. Statistical significance calculated by unpaired *t*-test, *n* = 8, *P* = 0.054. (**B**) Serum TMAO levels in conventional mice after inhibitor **45** treatment. TMAO levels decreased by ~40% when dosed at 30 mg/kg. Statistical significance calculated by unpaired *t*-test, *n* = 6, *P* = 0.027. (**C**) Abundance ratio of bacterial species as determined by COPRO-seq shows no significant differences in community composition before and after inhibitor **45** treatment at both low and high doses (See also Fig. S3B) (**D**) Relative abundance of phylum-level taxa from gut microbiomes of conventional mice before and after treatment with inhibitor **45** showed an increased proportion of Firmicutes and decreased proportion of Bacteroidota at the phylum level. The change in relative abundance was not statistically significant. (**E**) Differential abundance of genus-level taxa between groups showed no significant change between gut microbial community of conventional mice before and after inhibitor **45** treatment. Statistical significance was calculated by unpaired two-tailed Wilcoxon signed-rank test.

Mice were kept on a 1% choline diet and administered inhibitor **45** at a dose of either 3 mg/kg or 30 mg/kg daily via oral gavage for 3 days. Serum samples were collected 24 h after the last inhibitor dose and serum concentrations of TMAO were measured (see Fig. S3A). We observed reduced serum TMAO levels in the animals administered the higher inhibitor dose (Fig. 4A). This experiment was also replicated in conventional C57BL/6 mice with similar results (Fig. 4B). In both gnotobiotic and conventional mice, inhibitor treatment at the higher concentration lowered TMAO levels to about 60% of vehicle treatment, with *P*-values of 0.054 and 0.027, respectively (Fig. 4A and B). While the results in gnotobiotic mice were not statistically significant, we note that they reflected a similar trend to the results in conventional mice, which displayed a statistically significant reduction of serum TMAO. Despite the moderate reduction in serum TMAO levels, these results suggest the inhibitor is active *in vivo*.

Fecal samples from the gnotobiotic mice were also collected prior to and after inhibitor treatment, and the community was profiled as previously described (46). This analysis showed that inhibitor treatment at either concentration did not significantly alter community composition (Fig. 3C; Fig. S3B). Similarly, fecal samples collected from conventional mice before and after treatment with inhibitor **45** were sequenced. The alpha diversity of the gut microbial community moderately decreased after inhibitor **45** treatment (see Fig. S4A). PCoA analysis of unweighted and weighted UniFrac distances of fecal bacterial communities showed that mice from both inhibitor treatment groups were not distinguishable by beta diversity (see Fig. S4B and C). While the relative abundance of Firmicutes increased and that of Bacteroidota decreased after inhibitor **45** treatment, this was not statistically significant (Fig. 4D). There were also no significantly altered genus-level taxa after inhibitor **45** treatment (Fig. 4E). Taken together, these results suggest that inhibitor **45** does not significantly alter the composition of the gut microbial community at 30 mg/kg, a dose at which moderate reduction of serum TMAO is observed.

Finally, we measured the concentration of compound **45** in serum and cecal contents collected at the point of sacrifice of the gnotobiotic mice. Compound **45** was found at a concentration of 463 pmol/µg of wet cecal mass and was under the limit of detection (1 µM) in serum (see Fig. S5). This result suggested that compound **45** is retained in the gut at least 24 h after compound administration, confirming its presence at the site of choline metabolism. While this is only a single time point, the relatively low concentration of inhibitor in serum after 24 h could indicate that it is either metabolized quickly or is mostly not absorbed into the blood stream.

## DISCUSSION

We have designed a phenotypic HTS strategy to discover modulators of anaerobic gut microbial choline metabolism. This screen allowed for the rapid testing of a large chemical library and the identification of a novel scaffold that is less structurally similar to choline compared to existing inhibitors. We found that inhibition in the differential growth-based screen was a good predictor of inhibition of TMA production in whole cells (Table 1). Conversely, analogs optimized to inhibit TMA generation through our medicinal chemistry efforts also displayed the differential growth phenotype in choline- and glycerol-containing media (see Fig. S6), further validating the potential of using a growth-based readout for identifying choline metabolism inhibitors. The importance of the cyclic amine for activity suggests that it could potentially be involved in target engagement by mimicking choline or aiding the compound in entering cells. These inhibitors also did not display antibacterial activity at 200 µM, a concentration 100-fold higher than the EC_50_ of 2 µM required to half-maximally inhibit TMA generation in cultures. This may minimize potential unwanted shifts in gut microbiome composition due to inhibitor treatment. When tested in the gnotobiotic mouse model, inhibitor **45** did not significantly change the composition of the defined bacterial community, suggesting that inhibitor treatment at this concentration may not apply substantial selective pressure on the gut microbiome. Similarly, treatment with inhibitor **45** did not significantly shift the composition of the gut microbiome in conventional mice. In contrast, when choline metabolism is eliminated in this defined microbial community by replacing the wild-type *E. coli* strain with the ∆*cutC* strain, the abundance ratio of *C. aerofaciens* increases at the expense of *E. coli* (46). In conventional mice, inhibition of choline metabolism by IMC treatment induced a significant shift in the gut microbial community, with a notable increase in proportion of *Akkermansia* (13). Further studies on how inhibitor treatment may impact a more complex gut microbial community over time will be helpful in illuminating possible secondary effects beyond direct inhibition of TMA generation.

We also observed that while inhibitor **45** reduced the conversion of choline to TMA by bacteria efficiently *in vitro* (>80%), it had moderate efficacy *in vivo*, decreasing TMAO levels by approximately 40% compared to vehicle treatment. In contrast, previously reported halomethylcholine inhibitors completely inhibited TMAO production *in vivo* when dosed at 100 mg/kg, a concentration 3.3-fold higher than that used in our experiments, with an EC_50_ of 3.4 mg/kg and 45 mg/kg for fluoromethylcholine (FMC) and IMC, respectively (13). While preliminary animal experiments suggest that inhibitor **45** may be retained in the gut, further optimization of inhibitor dosage and an understanding of its pharmacokinetics may be required to improve *in vivo* efficacy.

A key consideration in developing small molecule inhibitors to study gut microbial metabolism is the potential metabolic crosstalk between microbiome and host. Many metabolites, dietary compounds, and xenobiotics can be metabolized by both gut microbial enzymes and human enzymes. As such, designing inhibitors that are specific for the microbial enzyme is critical for the compound to be a useful tool for disentangling host effects. Phenotypic HTS may be advantageous in this respect as it may be possible to identify novel chemical scaffolds that are distinct from the endogenous enzyme substrates. Although IMC and FMC are more potent than compound **45** *in vivo*, their structural similarity to choline may make them more likely to interact with host choline receptors, as evidenced by their potential neurotoxicity and the detection of related inhibitor metabolites in mice serum (13, 55).

While there is the possibility to discover new biological targets from phenotypic screening, another challenge associated with inhibitors discovered from this approach is the need for target identification. Because the gut microbiome harbors diverse bacterial taxa with different metabolic capabilities, it may be necessary to identify molecular targets and validate their functions in multiple organisms. Our compounds have varying activity against CutC *in vitro*, which suggests that CutC may be a potential target for some analogs. However, the inconsistent activity across whole cell and *in vitro* enzyme assays may point to alternative mode(s) of action. Preliminary assays investigating the effect of inhibitor **42** on choline uptake and transcription of the *cut* gene cluster yielded negative results. Further characterization of the cellular targets of these inhibitors will be required to better understand their mechanisms of action and potential off-target effects.

Although target-agnostic phenotypic screening is commonly used in antibiotic drug discovery, it has not yet been widely employed to discover non-lethal inhibitors of gut microbial metabolism. Major challenges associated with this screening approach include choosing an appropriate metabolic activity of interest and detection method. Methods that measure changes in levels of a specific metabolite of interest are often more resource and time intensive than measuring bacterial growth. However, it is not always straightforward to link a specific metabolic transformation of interest to growth, and not all bacteria can be grown in the lab in minimal media. For example, while we were able to culture choline-metabolizing *E. coli* in minimal media, this was not the case for other choline-metabolizing strains, such as *A. hydrogenalis* and *C. sporogenes*. Moreover, compounds may inhibit growth on a substrate, but not inhibit the specific metabolic transformation of interest. In the case of TMA generation, potential hits identified through the growth-based screen could potentially inhibit the downstream processing of the acetaldehyde byproduct rather than inhibit the transformation of choline to TMA. As such, previously used phenotypic screens focused on gut bacterial metabolism have tended to rely on MS-based detection methods (56). One notable exception is a recent report that screened extracts containing dietary phytochemicals for inhibition of the aerobic microbial transformation of L-carnitine to TMA by measuring growth of *Klebsiella pneumoniae* on media containing either L-carnitine or glucose as the sole carbon source and measuring resazurin reduction as a readout for metabolism (59). This proof-of-concept study screened 39 extracts and found 2 that could reduce TMA generation from L-carnitine *in vitro* by 84.6% and 61.3%. However, a major limitation of measuring resazurin reduction as a proxy for metabolic activity rather than optical density for growth is that anaerobes likely do not reduce resazurin (66). As such, optical density may be a more appropriate measurement when studying strictly anaerobic bacteria or metabolic processes. Our work similarly leveraged the knowledge that anaerobic choline metabolism is essential for growth only under certain conditions. A better understanding of how various bacterial enzymatic activities may be relevant to primary metabolism could allow for accelerated discovery of inhibitors through growth-based HTS.

The discovery of new inhibitors of anaerobic microbial choline metabolism in this work will inform future efforts to characterize biological targets involved in choline metabolism and to investigate how specifically modulating choline metabolism impacts host physiology. These inhibitors, or additional analogs, may be candidate tool compounds to study the link between TMAO generated through the anaerobic choline metabolism pathway and cardiometabolic diseases, or be developed as potential therapeutics for TMAU, a condition for which there are limited treatment options besides antibiotics (67). Because these inhibitors are structurally distinct from previously established inhibitors of anaerobic choline metabolism, their activity could not have been predicted or identified based on structure-based rational design. Our success in identifying novel inhibitors of anaerobic choline metabolism using this differential growth-based phenotypic HTS not only demonstrates the utility of this approach but also suggests that it may be applied more broadly to identify inhibitors of other gut bacterial metabolic activities of interest.

## MATERIALS AND METHODS

### Reagents and resources

Reagents and resources are listed in Table 4. Requests for further information and requests for resources and reagents should be directed to the corresponding author.

**TABLE 4:** Reagents and resources

Reagent or resource	Source	Identifier
Bacterial and virus strains		
*Escherichia coli*	BEI	MS 200-1
*Clostridium sporogenes*	ATCC	15579
*Proteus mirabilis*	ATCC	29906
*Anaerococcus hydrogenalis*	DSMZ	DSM 7454
*Bacteroides thetaiotaomicron* VPI-5482	ATCC	29148
*Bacteroides ovatus*	ATCC	8483
*Bacteroides caccae*	ATCC	43185
*Collinsella aerofaciens*	ATCC	25986
*Eubacterium rectale*	ATCC	33656
Chemicals, peptides, and recombinant proteins		
(Trimethyl-d_9_)-choline chloride	Cambridge Isotope Laboratories	DLM-549-PK
d_9_-trimethylamine hydrochloride	Sigma Aldrich	613843
Deposited data		
High-throughput screening raw data	PubChem	AID 1963822
Experimental models: organisms/strains		
*Mus musculus* C57BL/6	Germ-free mice raised in the gnotobiotic facility at the University of Wisconsin–Madison	
*Mus musculus* C57BL/6	Conventional mice raised in the gnotobiotic facility at the University of Wisconsin–Madison	
Software and algorithms		
MassHunter Workstation Qualitative Analysis	Agilent Technologies	
GraphPad Prism 10	GraphPad Software	graphpad.com

### Experimental model and subject details

#### *In vivo* animal studies

##### Preparation of core gut microbial community

A core community of five gut bacterial species*—Bacteroides caccae* ATCC 43185, *Bacteroides ovatus* ATCC 8483, *Bacteroides thetaiotaomicron* VPI-5482, *Collinsella aerofaciens* ATCC 25986, and *Eubacterium rectale* ATCC 33656—as well as *Escherichia coli* MS 200-1 obtained from the Balskus lab, were cultured in CMM medium at 37°C in an anaerobic environment. One milliliter of culture from each of the six individually grown strains was added to create this mixed culture.

##### Preparation of inhibitor

In a biosafety cabinet, a 300 mg/mL stock of sterile inhibitor in filter-sterilized DMSO was prepared. After this preparation, a working solution of 3 mg/mL of the inhibitor stock in sterile 1% hydroxyethyl cellulose/1% polysorbate 80 was prepared within 4 days of gavaging the mice and stored at room temperature. A low dose of the inhibitor was also prepared, using a working solution of 0.3 mg/mL of the inhibitor stock in sterile 1% hydroxyethyl cellulose/1% polysorbate 80. The vehicle was prepared in the same way with DMSO without inhibitor.

##### Inhibitor treatment and sample collection

Female C57BL/6 germ-free mice (*n* = 8 per inhibitor group), 10–12 weeks of age in an isolator, were colonized with 200 µL of mix containing the five bacterial species mentioned above at ~10^8^ bacterial cells/mL. Mice were fed a 1% choline diet (Envigo) *ad libitum*. Two weeks after colonization, mice were administrated by gavage with 3 mg/kg of the inhibitor or vehicle between 6 and 8 a.m. once a day for three consecutive days. After a 10-day washout period, mice were orally administered 30 mg/kg of the inhibitor once a day (same time as above) for three consecutive days, and blood was collected 24 h after the last gavage.

Eight week old conventionally raised female C57BL/6 mice (*n* = 6 per inhibitor group) were fed a 1% choline diet (Envigo) *ad libitum* for 10–13 days. All mice were administered by gavage with 3 mg/kg or 30 mg/kg of the inhibitor once a day (between 6 and 8 a.m.) for three consecutive days.

A fecal sample was collected before the first gavage. At sacrifice, tissues collected were blood, cecal contents, a fecal pellet, proximal colon, distal small intestine, liver, and kidney. Mice were anesthetized with isoflurane and underwent cardiac puncture for a terminal blood draw, followed by cervical dislocation. Tissues were snap-frozen in liquid nitrogen. Blood was spun down at 8,000 × *g* for 8 min at room temperature; the resulting serum layer was removed and snap-frozen. All snap-frozen tissues were stored at −80°C until needed.

### Method details

#### Bacterial culture conditions and media preparation

Bacteria were grown overnight in an anaerobic chamber (Coy Laboratories) at 37°C under an atmosphere of either 95% N_2_ and 5% H_2_ for the HTS and TMA inhibition EC_50_ assays or 75% N_2_, 20% CO_2_, and 5% H_2_ for MIC assays. For TMA inhibition assays, *E. coli* and *P. mirabilis* were grown in NCE medium supplemented with 20 mM choline chloride, *A. hydrogenalis* was grown in MEGA medium supplemented with 3 mM choline chloride, and *C. sporogenes* was grown in BHI medium supplemented with 20 mM of choline chloride to induce expression of the *cut* gene cluster. For MIC assays, all bacteria strains were grown in MEGA medium.

NCE media was adapted based on the “no carbon E” media as previously described (14), and contains 29 mM potassium phosphate monobasic, 29 mM potassium phosphate dibasic, 17 mM ammonium sodium phosphate dibasic, 40 mM sodium fumarate dibasic, 0.1% (wt/vol) casamino acids, and either 20 mM choline chloride or 3 g/L glycerol. This mixture was supplemented with 1 mM magnesium sulfate, 1% ATCC vitamin supplement, and 1% ATCC trace mineral supplement.

MEGA medium was prepared as previously described (61), and CMM medium was prepared by supplementing MEGA medium with 0.9 g of D-maltose, 0.86 g of D-cellobiose, and 0.43 g of D-fructose per L of medium.

#### Growth-based phenotypic high-throughput screening

Compounds from the Institute of Chemistry and Cell Biology-Longwood Screening Facility (ICCB-L) commercial ChemDiv7 library were transferred to 384-well plates at 5 mg/mL in DMSO.

*E. coli* was grown anaerobically in LB media containing 1 mM choline chloride overnight at 37°C and then resuspended (two log-fold dilution) in either NCE media supplemented with 20 mM choline or 3 g/L glycerol for screening. The *E. coli* cultures were then added to the compound plates and incubated at 37°C anaerobically. The final concentration of compounds in culture was 20 µg/mL. OD_600_ was measured at 7 h and 20 h in duplicate compound plates. For control, 60 µg/mL kanamycin was used. Z-scores of each compound were calculated by measuring the number of standard deviations of each well’s OD_600_ with respect to the plate’s mean OD_600_. Compounds with a Z-score of less than –2 on choline but more than or equal to 0 on glycerol, in at least one replicate plate, were selected for further validation. Z’ was calculated using the control columns, as an internal quality control.

#### Determination of EC_50_ for TMA inhibition in bacterial whole cells

Compounds were stored as 10 mM DMSO stocks at –20°C and diluted in DMSO to prepare varying concentrations of inhibitors for testing. Bacteria were grown anaerobically overnight in media supplemented with choline as described above. Cells were pelleted and resuspended in PBS, and d_9_-choline chloride was added to a final concentration of 1 mM. The final OD_600_ of the PBS cell resuspensions is as follows: 0.4 (*E. coli*), 0.15–0.2 (*P. mirabilis*), 0.2 (*C. sporogenes*), and 0.4 (*A. hydrogenalis*). The resuspended cells were added to 96-well plates containing inhibitors (final concentration of 1% DMSO, with final inhibitor concentrations ranging between 0 and 325 µM). The plates were sealed and incubated for 75 min (or 90 min for *P. mirabilis*) at 37°C. Following incubation, the plates were removed from the anaerobic chamber and analyzed immediately or kept at –20°C until LC–MS/MS analysis. Each inhibitor concentration was tested in triplicate. EC_50_ values were calculated with GraphPad Prism by fitting the log(inhibitor) vs response—Variable slope (four-parameter) model for non-linear regression.

#### Determination of MIC against bacterial panel

Compounds were diluted in DMSO to prepare a series of concentrations ranging from 0 to 10 mM in a 384-well plate. From this compound plate, 1 µL of each compound concentration was transferred into three new 384-well plates. Overnight cultures of bacteria in MEGA media were resuspended in fresh MEGA media (1% inoculum), and 49 µL of these bacterial cultures were added to each well containing compounds. The final concentration of compounds in culture ranged between 0 and 200 µM. DMSO was used as the growth control, and media was used as the sterile control. Plates were incubated anaerobically at 37°C for 24 h, and OD_600_ was measured after incubation. Percent growth of wells was calculated with the following formula: (sample OD_600_ – sterile control OD_600_)/(DMSO control OD_600_ – sterile control OD_600_) × 100%. Wells with less than 50% growth were determined to have no growth. Starter cultures of *B. caccae*, *C. aerofaciens*, and *E. rectale* were grown for 2 days rather than overnight before testing as they grow slowly.

#### Determination of IC_50_ toward purified CutC enzymes

CutC and CutD enzymes from *Desulfovibrio desulfuricans* G20 were purified, CutC was activated by incubation with CutD, and the amount of glycyl radical installation was quantified as previously described (15). The IC_50_ of compounds was determined in an *in vitro* CutC activity assay, where CutC activity is coupled to the NADH-dependent enzymatic reduction of acetaldehyde by YADH (15). Briefly, assays were conducted in an anaerobic chamber (MBraun) under N_2_ atmosphere at 20°C. In 50 mM potassium phosphate buffer (pH 8.0, 50 mM KCl), 40 µM of CutD and 150 µM of sodium dithionite were incubated for 20 min before the addition of 200 µM of SAM and 20 µM of CutC monomer. The mixture was incubated for 1 h to activate the glycyl radical enzyme, after which the activated CutC mixture was diluted 20-fold in 50 mM potassium phosphate buffer (pH 8.0, 50 mM KCl). A master mix containing 200 µM NADH, 0.4 µM YADH (Sigma), and the diluted CutC mixture (5 nM final concentration in assay) was prepared and aliquoted into 96-well plates (180 µL per well). Ten microliters of inhibitor or DMSO vehicle was added, followed by 10 µL of aqueous choline chloride solution (final concentration 200 µM). Absorbance was measured at 340 nm immediately after addition of all assay components, with measurements taken every 20 s for 10 min. Initial rates were calculated from absorbance measurements and fitted to Michaelis–Menten equation using nonlinear regression in GraphPad Prism 10 (v. 10.4.0). Background activity was subtracted using no substrate controls, and initial rates for each inhibitor concentration were normalized to no inhibitor controls. IC_50_ values were obtained by plotting normalized activity values against the logarithm of inhibitor concentration and fitting to a nonlinear regression model in GraphPad Prism 10 (v. 10.4.0).

#### Quantification of gene transcripts with Nanostring

Nanostring probes (proprietary) were designed and synthesized based on the DNA sequences of housekeeping genes (*gyrA*, *rpoB*), the choline utilization gene cluster (*cutW*, *cutX*, *cmcA*, *cmcB*, *cmcC*, *cutF*, *cmcD*, *cutO*, *cutC*, *cutD*, *cmcE*, *cutH*, *cutU*, *cutV*), and putative quaternary amine transport genes (*betT*, *caiT*, *proV*, *proW*, *proX*, *gdx*, *emrE*) in *E. coli* MS 200-1. *E. coli* MS 200-1 cells were grown anaerobically overnight in NCE medium supplemented with 3 g/L glycerol. The cells were then inoculated to 2% volume in fresh NCE glycerol medium containing either vehicle (DMSO) or 10 µM of inhibitor **42**. After 13 h of anaerobic growth at 37°C (OD_600_ 0.4–0.5), choline chloride was spiked into the samples at a final concentration of 1 mM to induce expression of the choline utilization gene cluster. Cells were harvested 1.5 h after induction, and RNA was isolated using RNeasy Mini Kit (Qiagen). RNA was hybridized with the Nanostring probes by incubating at 67°C for 18 h and analyzed on the Nanostring nCounter SPRINT instrument to measure differential gene expression. OD_600_ measurements were taken before and after choline induction to ensure that cells were in log phase. Data analysis was performed using nSolver Analysis Software (version 4.0.70). Transcripts were considered differentially regulated if log_2_ fold change > 1 and *P*-value < 0.05 based on *t*-test.

#### Quantification of [^14^C]-choline transport

*E. coli* MS 200-1 ∆*cutC* was grown either aerobically or anaerobically in LB medium supplemented with 1 mM choline chloride. Cells were pelleted and resuspended in fresh PBS after overnight growth to OD_600_ of 1 and aliquoted into microcentrifuge tubes. Either vehicle (DMSO) or 10 µM of inhibitor **42** was added to the cell resuspensions and incubated for 15 min at room temperature, either aerobically or anaerobically. 5 µM [^14^C] choline was added to the cells and left to incubate for 15 mins aerobically. At the end of the incubation period, cells were washed with PBS three times by centrifugation at 3,000 × *g* for 5 min and resuspended in equal volume of PBS. The resulting cell resuspension in PBS was transferred to a scintillation vial, where 10 mL of liquid scintillation cocktail was added and mixed. Radioactivity was counted using a Beckman Coulter LS6000 liquid scintillation counter for 1 min per sample.

#### Quantification of d_9_-TMA and d_9_-choline using LC–MS/MS

Samples were kept frozen until analysis and kept cold on ice during handling due to the volatility of TMA. Bacterial cell resuspensions in PBS were diluted 100-fold in LCMS-grade acetonitrile with 0.1% formic acid to lyse cells, precipitate proteins, and extract metabolites. Samples were centrifuged at 4,000 rpm for 10 min at 4°C to pellet proteins. The supernatants were then further diluted 20-fold in LCMS-grade acetonitrile with 0.1% formic acid, and the resulting solution was analyzed by LC–MS/MS.

LC–MS/MS analysis was performed on an Agilent 1290/6470 Triple Quadrupole LC–MS instrument (Agilent Technologies) using electrospray ionization. The mass spectrometer was operated in multiple reaction monitoring (MRM) mode with positive ionization monitoring. The precursor–product ion pairs used in MRM mode were as follows: *m*/*z* 69.1*m*/*z* → 49.1 (d_9_-TMA) and *m*/*z* 113.2 *m*/*z* → 69.1 (d_9_-choline). The capillary voltage was set to 4.0 kV, and the fragmentor voltage was set to 30 V (d_9_-TMA) and 115 V (d_9_-choline). The collision energy for the precursor–product ion pairs was set to 29 V (d_9_-TMA) and 21 V (d_9_-choline). MS^1^ resolution was set to wide and MS^2^ resolution to unit. The drying gas temperature was maintained at 250°C, with a flow rate of 11 L/min and a nebulizer pressure of 45 psi. The time filter width used was 0.07 min. The injection volume of all samples was 1 μL. Samples were injected onto InfinityLab Poroshell 120 (Agilent) HILIC columns (2.7 μm, 100 mm × 2.1 mm, 100 Å) preceded by InfinityLab Poroshell 120 HILIC guard columns (2.1 mm, 1.9µm UHPLC guard). The LC conditions on the binary pump were as follows: a gradient of 5–35% A increasing over 1.95 min (1.2 mL/min), a gradient of 35–5% A decreasing over 0.05 min (1.6 mL/min), and held at 5% A for 0.5 min (2 mL/min). The columns were regenerated on the quaternary pump with the following conditions: 60% A for 1.25 min (1.2 mL/min), a decreasing gradient of 60–5% A over 0.05 min (1.2 mL/min), and held at 5% A for 0.5 min (1.5 mL/min). Solvent A was 10 mM ammonium formate with 0.1% formic acid, and solvent B was acetonitrile with 0.1% formic acid. Data analysis was performed with Mass Hunter Workstation Data Acquisition and Qualitative Analysis software (Agilent Technologies).

#### Quantification of TMAO in mouse serum samples using LC–MS/MS

One volume of plasma and 4 volumes of ice-cold extraction solution (HPLC-grade methanol with 2.5 µM d_9_-TMAO internal standard) were combined and spun at 21,100 × *g* for 3 min at 4°C. One volume of the resulting supernatant was then mixed with 1 volume of HPLC-grade water. The resulting samples were injected onto a Waters ACQUITY C18 UPLC column (1.7 µm, 2.1 mm × 100 mm) that was coupled to a Thermo Fisher Q-Exactive mass spectrometer at a flow rate of 0.2 mL/min. Elution of samples occurred over a 7 min isocratic gradient of 25% water, 5 mM ammonium acetate, 0.05% acetic acid, and 75% methanol. TMAO was quantified in the positive mode using Parallel Reaction Monitoring (PRM) with an inclusion list of 76.076 and 85.132 *m*/*z* (for TMAO and d_9_-TMAO, respectively). El-MAVEN was used for peak quantification, and internal standards were utilized as a comparison to calculate plasma concentrations. Statistical significance was calculated using an unpaired *t*-test.

#### Quantification of inhibitor 45 in serum and cecal contents of mice using LC–MS/MS

A standard curve of inhibitor **45** spiked in commercial mouse serum (Sigma) was prepared. To 50 µL of serum sample, 450 µL of a mixture 75% acetonitrile, 25% methanol, 1% formic acid was added to precipitate proteins. Samples were centrifuged at 21,000 rcf for 10 min at 4°C. Phospholipids were removed by aspirating and dispensing the supernatants using HybridSPE DPX (Sigma-Aldrich) tips. The resulting samples were aliquoted into 96-well plates for LC-MS analysis.

Cecal samples were thawed and weighed into bead-beating tubes. An extraction solvent of 75% acetonitrile, 25% methanol, and 1% formic acid was prepared. A 100 µM internal standard solution was prepared in the extraction solvent. To each sample, 5 µL of internal standard solution was added and 500 µL of extraction solvent (final concentration of internal standard 1 µM). Tubes were cooled on ice, and a bead beater was used to homogenize samples for 2 min. Samples were rested on ice while processing other samples, then were homogenized again for 2 min. Samples were cooled on ice for at least 5 min before centrifuging for 10 min at 21,000 rcf. Supernatants were transferred into Captiva EMR-lipid plates (Agilent) and centrifuged at 500 rpm for 1 min into 96-well plates for LC-MS analysis.

LC-MS/MS analysis was performed on an Agilent 1290/6470 Triple Quadrupole LC-MS instrument (Agilent Technologies) using electrospray ionization. The mass spectrometer was operated in multiple reaction monitoring (MRM) mode with positive ionization. The precursor–product ion pair used in MRM mode was *m*/*z* 396.3*m*/*z* → 212.1 (inhibitor **45**). The capillary voltage was set to 4.0 kV, and the fragmentor voltage was to 162 V. The collision energy for the precursor–product ion pairs was set to 36 V. MS^1^ resolution was set to wide and MS^2^ resolution to unit. The drying gas temperature was maintained at 250°C, with a flow rate of 11 L/min and a nebulizer pressure of 45 psi. The time filter width used was 0.07 min. The injection volume of all samples was 1 μL. Samples were injected onto InfinityLab Poroshell 120 (Agilent) EC-C18 columns (1.9 μm, 50 mm × 2.1 mm, 100 Å) preceded by InfinityLab Poroshell 120 (Agilent) EC-C18 guard columns (2.1 mm, 1.9µm UHPLC guard). The LC conditions on the binary pump were as follows: 95% A for 0.25 min, a gradient of 95–50% A decreasing over 0.25 min, held at 50% A for 1 min, a gradient of 50–5% A decreasing over 0.5 min, and held at 5% A for 3 min (0.8 mL/min flow rate throughout). The columns were regenerated on the quaternary pump with the following conditions: 5% A for 3.5 min, an increasing gradient of 5–95% A over 0.5 min, and held at 95% A for 1 min (1 mL/min throughout). Solvent A was water with 0.1% formic acid, and solvent B was acetonitrile with 0.1% formic acid. Data analysis was performed using Mass Hunter Workstation Data Acquisition and Qualitative Analysis software (Agilent Technologies).

#### Sample preparation and sequencing of mouse fecal samples

DNA was extracted from fecal samples according to published bead-beating procedures (68, 69), and pellets were dried with ethanol and resuspended in 10 mM Tris-HCl, pH 8.5. NucleoSpin Gel and PCR Clean-up Kit (Macherey-Nagel) was used to remove contaminants. Isolated DNA was stored at −80°C until downstream processing. Libraries were prepared according to the manufacturer’s protocol (15,031,942 v05) for Illumina Nextera XT DNA Library Preparation Kit (Illumina). Briefly, 1.0 ng of each input DNA was enzymatically fragmented and tagged by fragmentation. The cleaved DNA was subjected to limited-cycle PCR for indexing with i5 and i7 index adaptors. The PCR products were purified using Agencourt AMPure XP beads (Beckman Coulter) and normalized using the Nextera XT Library Normalization Beads (Illumina). The normalized libraries were diluted and pooled at final loading concentration of 150 pM, according to the manufacturer’s protocol (1000000036024 v07). The pooled library was spiked with 2% non-denatured TailorMix Dual Indexed PhiX Control Library (SeqMatic) and sequenced with Illumina iSeq 100 (v3.0.0.359, 2×150 cycles, paired-end). Quality control monitoring of the library preparation process was performed using agarose gel electrophoresis. Results were processed using the software pipeline detailed by McNulty et al. (70).

#### Microbiome 16S sequencing and analysis

DNA from flash-frozen fecal samples was extracted via bead-beating with phenol-chloroform and cleaned as described previously (71). Purified DNA was used for 16S rRNA V4 library preparation. Sequencing was performed at the UW-Madison Next Generation Sequencing Core using an Illumina MiSeq. The resulting 16S rRNA amplicon sequences were demultiplexed and cleaned using the DADA2 package (72). QC and removal of chimeric reads resulted in an average of 141,867 reads per sample. ASV taxonomy was assigned using a pre-trained SILVA classifier (silva-138-99-nb-classifier) in QIIME2 (73). Alpha and beta diversity were calculated at a rarefaction depth of 10,000 sequences per sample. Weighted and unweighted UniFrac distances were calculated using ASV relative abundance tables with phylogenetic trees and used to generate principal coordinate analysis (PCoA) ordination plots. Differential abundance of genus-level features between groups was calculated using unpaired two-tailed Wilcoxon signed-rank test. For multiple testing, the Benjamini–Hochberg false discovery rate (FDR) procedure was used to adjust *P* values.

### Quantification and statistical analysis

All data fitting and statistical analysis were performed using GraphPad Prism version 10 (GraphPad Software, La Jolla, California, USA, https://www.graphpad.com/). Statistical values and statistical significance were also reported in the corresponding figure or figure legends.

## Data Availability

The HTS screening data reported in the paper is deposited in PubChem under the PubChem AID 1963822.

## SUPPLEMENTAL MATERIAL

Document S1 mbio.01172-25-s0001.pdf Figures S1-S6 and Tables S1-S5.

Document S2 mbio.01172-25-s0002.pdf Synthetic procedures of compounds 5-45.

Document S3 mbio.01172-25-s0003.pdf NMR spectra of synthesized compounds 5-45.

ASM does not own the copyrights to Supplemental Material that may be linked to, or accessed through, an article. The authors have granted ASM a non-exclusive, world-wide license to publish the Supplemental Material files. Please contact the corresponding author directly for reuse.
