# Supplementary material for: Phenotypic high-throughput screening identifies modulators of gut microbial choline metabolism
Source: mBio. 2026 Feb 23;17(3):e01172-25. doi: 10.1128/mbio.01172-25 (PMC12977544; doi:10.1128/mbio.01172-25)
Supplement: Document S1 — Figures S1-S6 and Tables S1-S5. [file mbio.01172-25-s0001.pdf]

Supplementary Figures

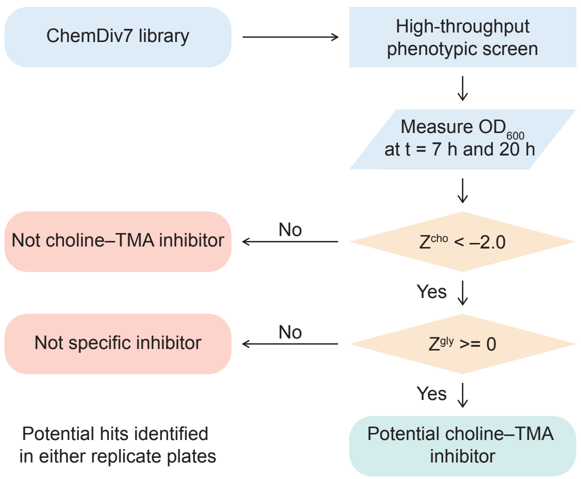

Fig S1: Flowchart of data analysis pipeline to identify hit compounds in the HTS primary screen. Compounds on either replicate plate at either 7 h or 20 h time points with Z-scores of < –2.0 in choline and >= 0 in glycerol were considered potential hits.

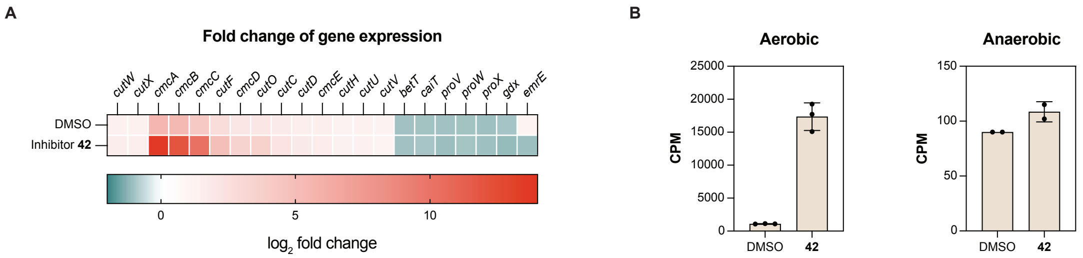

Fig S2: Preliminary mode of action assays. (A) Inhibitor **42** does not repress the expression of the *cut* gene cluster significantly in *E. coli*. Assays were performed in triplicates. Data are represented as mean ± SEM. Student's t-test, n = 3. (B) Inhibitor **42** does not inhibit choline uptake in *E. coli* when cells were grown aerobically or anaerobically. Assays were performed in triplicates for aerobic growth and duplicates for anaerobic growth. Data are represented as mean ± SD.

A

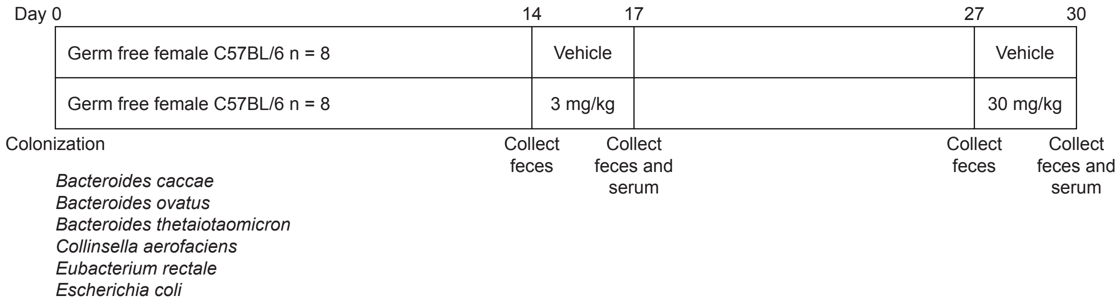

B

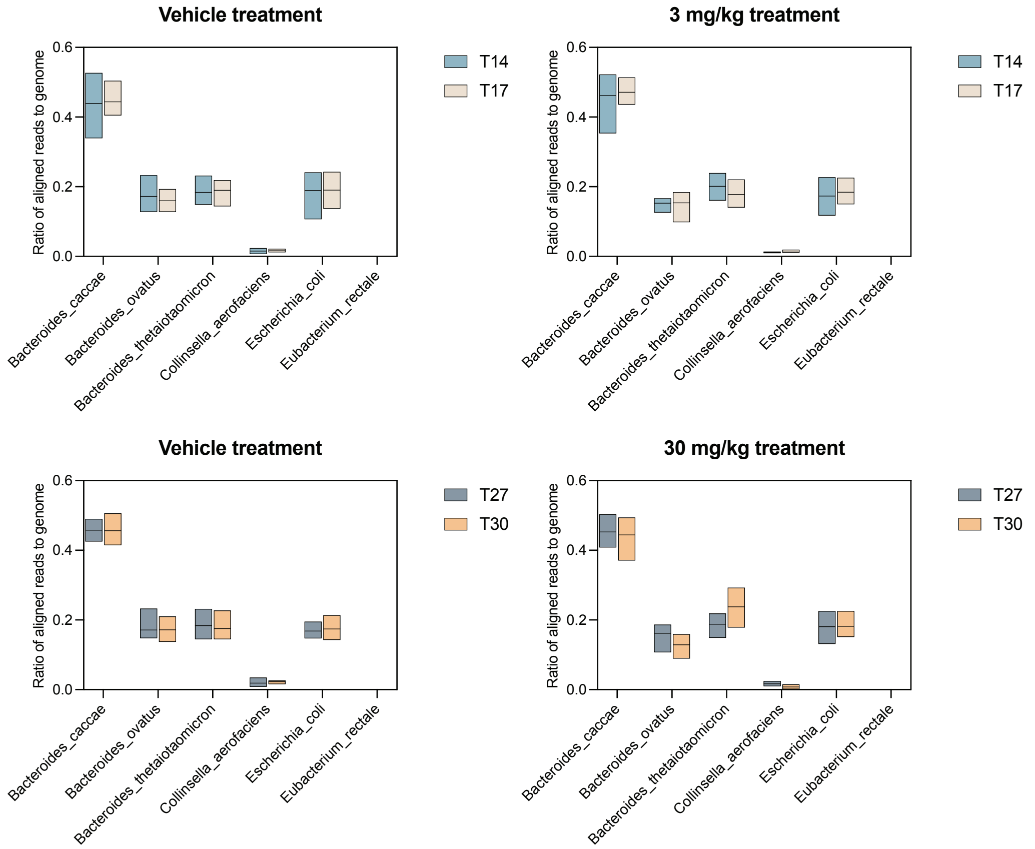

Fig S3: Inhibitor treatment in gnotobiotic mice. (A) Timeline of colonization, inhibitor dosage, and sample collection. (B) Abundance ratios of bacterial species present in fecal samples as determined by COPRO-seq shows no statistically significant differences in abundance of each bacterial species before and after vehicle treatment, and inhibitor treatment at both dosages. Data are represented as mean ± SEM. Wilcoxon-sign ranked test, n = 8.

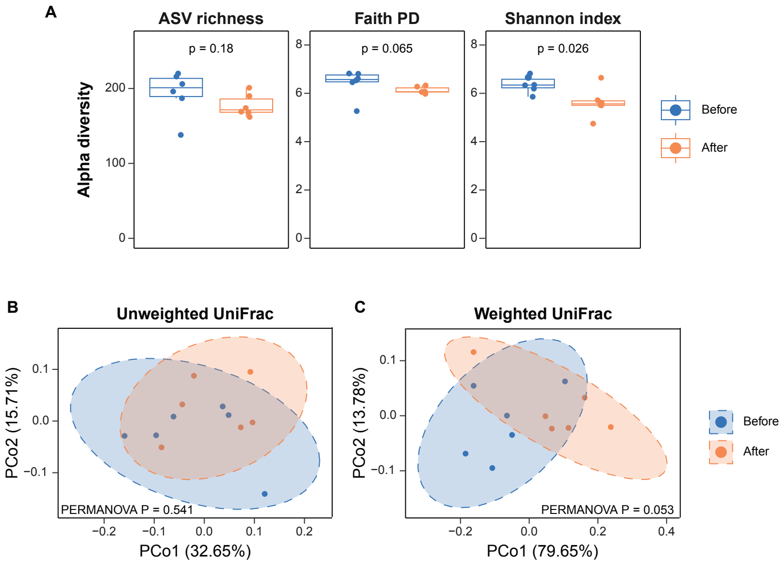

Fig S4: Gut microbiome composition of conventional mice upon 30 mg/kg inhibitor treatment. (A) Alpha diversity (Faith's phylogenetic diversity and Shannon index) of gut microbiome community of conventional mice decreased after inhibitor **45** treatment. (B) PCoA plot using unweighted UniFrac distances of 16S rRNA ASV profiles from gut microbiome community before and after inhibitor **45** treatment. (C) PCoA plot using weighted UniFrac distances of 16S rRNA ASV profiles from gut microbiome community before and after inhibitor **45** treatment. Samples from before and after inhibitor treatment were not distinguishable by beta diversity.

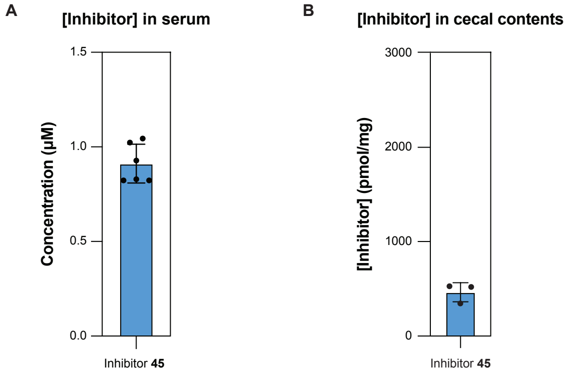

Fig S5: Measurement of inhibitor **45** concentration in gnotobiotic mice samples. (A) Inhibitor **45** was below the limit of detection of 1 μM in serum samples 24 h after inhibitor gavage. Data are represented as mean ± SD, n = 6. (B) Inhibitor **45** was detected at a concentration of 463 pmol/μg in wet mass of cecal contents 24 h after inhibitor gavage. Data are represented as mean ± SD, n = 3

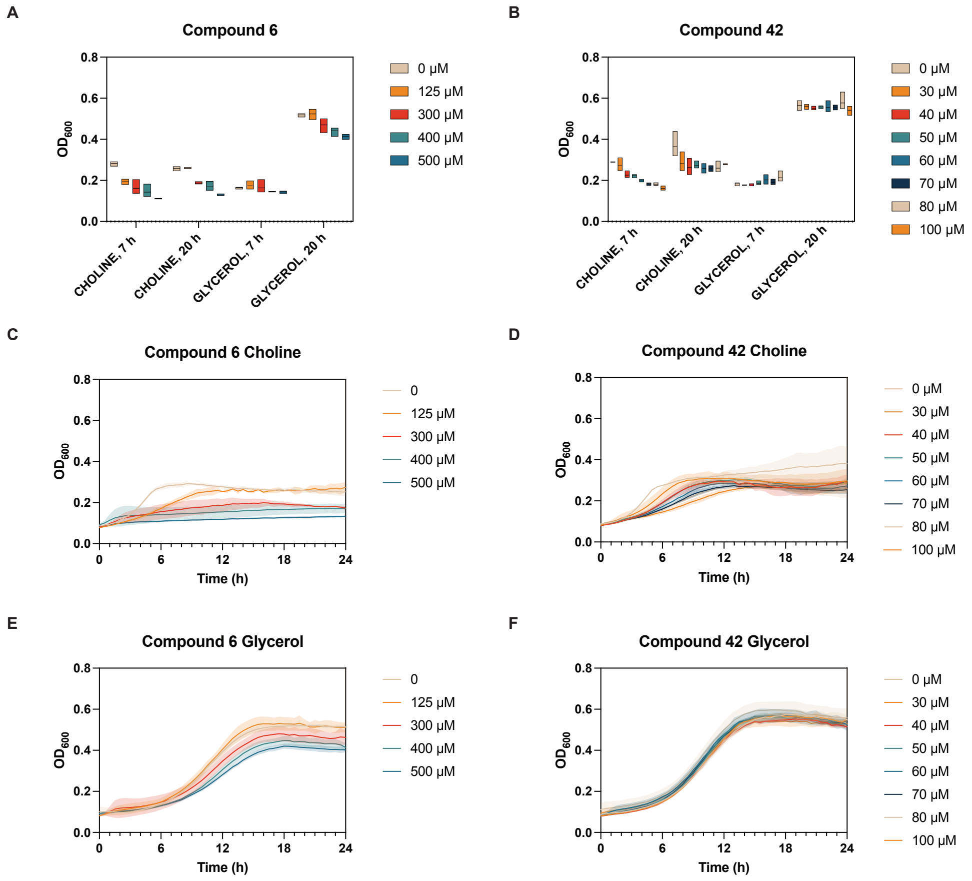

Fig S6: Growth inhibition of *E. coli* cultures in choline- and glycerol-containing minimal media. (A, B) Compounds **6** and **42** inhibit *E. coli* growth on choline in a dose-dependent manner at 7 h, but not on glycerol. This differential growth was similarly observed with the initial HTS hit compound **5**. (C–F) Full growth curves of *E. coli* in choline and glycerol minimal media in the presence of varying concentrations of compounds **6** and **42**. For both compounds, growth in choline is delayed as concentration of inhibitors increase, which is not observed in glycerol. Assays were performed in triplicates. Data are represented as mean ± SEM.

Table S1: EC_{50} values of compounds **6**–**14** with varying heterocyclic cores

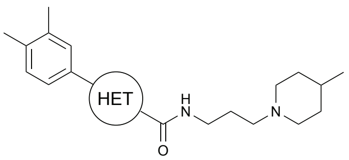

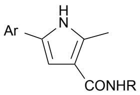

6

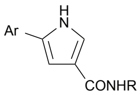

7

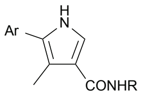

8

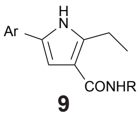

9

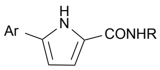

10

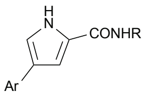

11

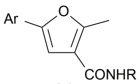

12

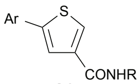

13

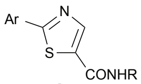

14

EC_{50} in whole cells (μM)

Compound	*E. coli*	*P. mirabilis*	*C. sporogenes*	*A. hydrogenalis*
**6**	9	48	132	31
**7**	16	80	215	66
**8**	>125	131	97	N/A
**9**	35	N/A	103	N/A
**10**	98	63	312	N/A
**11**	13	42	>312	N/A
**12**	15	>312	54	54
**13**	14	50	85	N/A
**14**	27	197	215	N/A

*N/A denotes that compound was not tested against bacterial strain.

Table S2: EC_{50} values of compounds **6**, **15**–**19** with varying *N*-pyrrole substituents

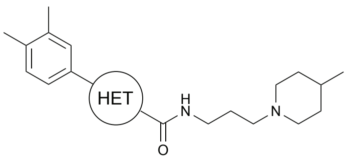

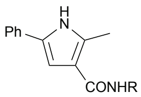

6

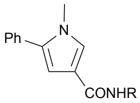

15

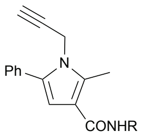

16

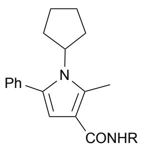

17

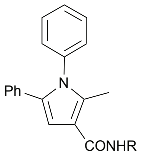

18

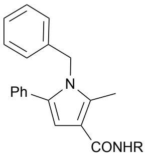

19

	EC_{50} in whole cells (μM)			
Compound	*E. coli*	*P. mirabilis*	*C. sporogenes*	*A. hydrogenalis*
**6**	9	48	132	31
**15**	21	98	114	116
**16**	39	N/A	5	N/A
**17**	69	N/A	8	N/A
**18**	489	N/A	3	N/A
**19**	28	N/A	4	N/A

*N/A denotes that compound was not tested against bacterial strain.

Table S3: EC_{50} values of compounds **6**, **20**–**26** with varying substituents on C5

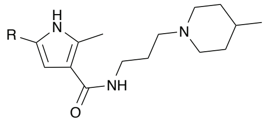

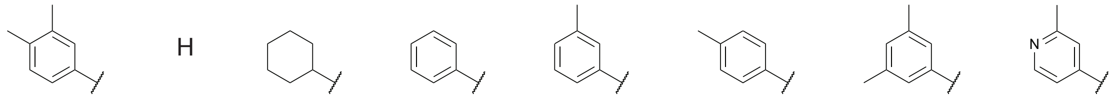

6 **20** **21** **22** **23** **24** **25** **26**

	**EC_{50} in whole cells (μM)**			
Compound	*E. coli*	*P. mirabilis*	*C. sporogenes*	*A. hydrogenalis*
**6**	9	48	132	31
**20**	>125	No inhibition at 312	No inhibition at 312	No inhibition at 312
**21**	51	N/A	8	32
**22**	7	157	147	24
**23**	5	46	95	35
**24**	92	131	117	N/A
**25**	6	28	133	19
**26**	>125	>312	No inhibition at 312	No inhibition at 312

Table S4: EC_{50} values of compounds **6**, **27**, **28** with varying substituents on the C3 carboxamide

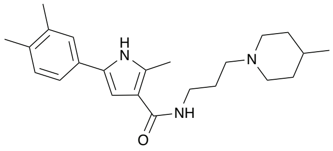

6

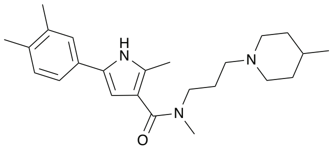

27

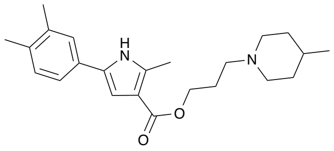

28

EC_{50} in whole cells (μM)

Compound	*E. coli*	*P. mirabilis*	*C. sporogenes*	*A. hydrogenalis*
**6**	9	48	132	31
**27**	>125	>312	12	44
**28**	15	>312	18	11

*N/A denotes that compound was not tested against bacterial strain.

Table S5: MIC values of select compounds against panel of commensal bacterial strains

	MIC in MEGA medium (µM)			
Compound	*E. coli*	*P. mirabilis*	*C. sporogenes*	*A. hydrogenalis*
**6**	>200	>200	>200	200
**19**	>200	>200	>200	25
**23**	>200	>200	>200	>200
**44**	>200	>200	>200	>200
**45**	>200	>200	>200	>200

	MIC in MEGA medium (µM)				
Compound	*B. thetaiotaomicron*	*B. ovatus*	*B. caccae*	*C. aerofaciens*	*E. rectale*
**6**	>200	>200	>200	>200	200
**19**	50	25	>200	6.25	25
**23**	>200	>200	>200	200	>200
**44**	>200	>200	>200	>200	N/A
**45**	>200	>200	>200	>200	>200

*N/A denotes that compound was not tested against bacterial strain.
