# Supplementary material for: Phenotypic high-throughput screening identifies modulators of gut microbial choline metabolism
Source: mBio. 2026 Feb 23;17(3):e01172-25. doi: 10.1128/mbio.01172-25 (PMC12977544; doi:10.1128/mbio.01172-25)
Supplement: Document S3 — NMR spectra of synthesized compounds 5-45. [file mbio.01172-25-s0003.pdf]

^{1}H and ^{13}C NMR spectra of synthesized compounds 5–45

Compound 5

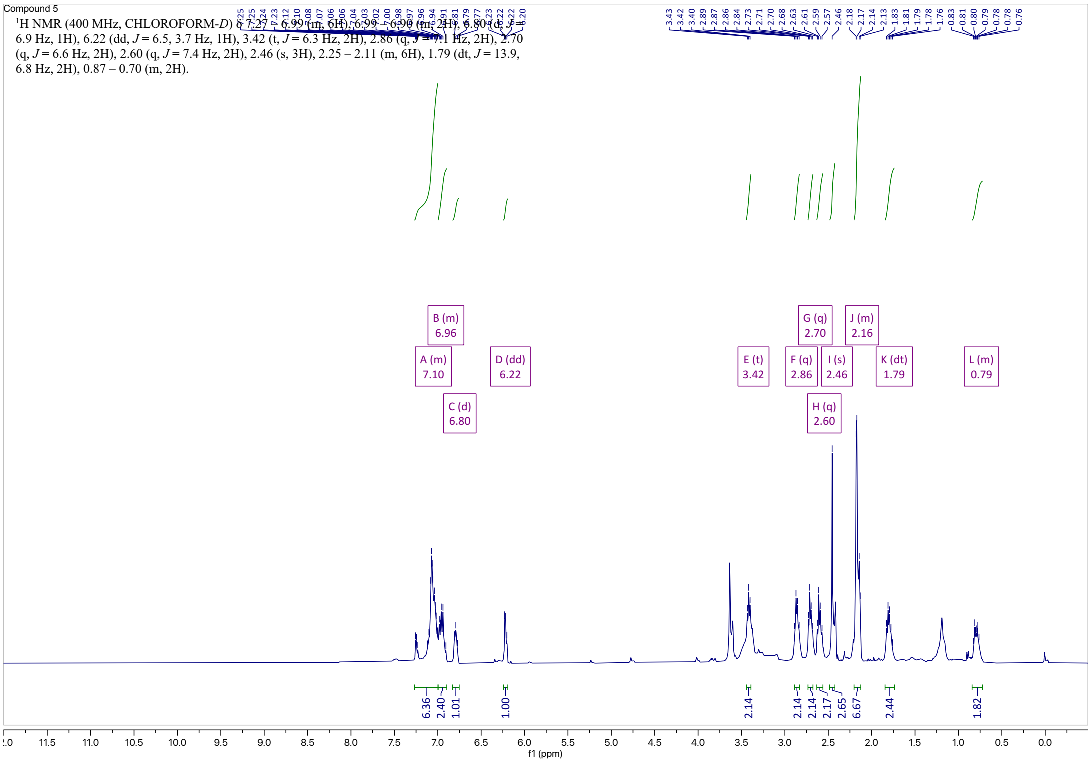

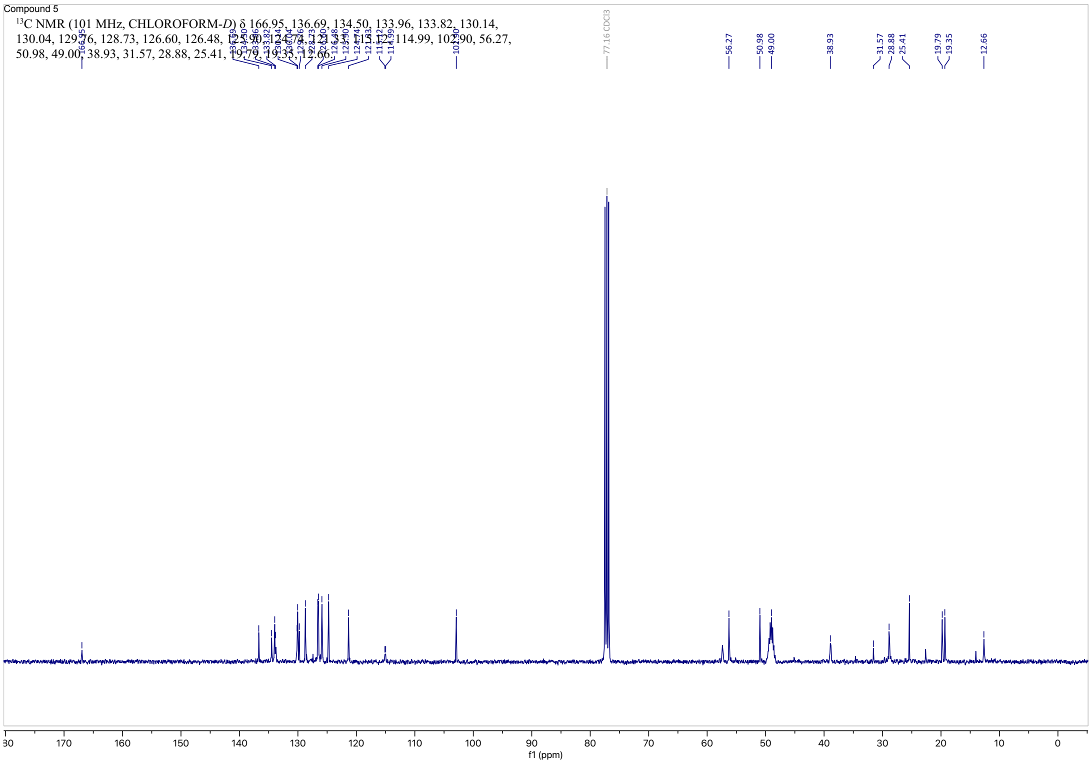

Compound 6

Compound 6

^{1}H NMR (400 MHz, DMSO-D_6) δ 11.21 (s, 1H), 7.66 (s, 1H), 7.36 (s, 1H), 7.29 (d, J = 7.1 Hz, 1H), 7.10 (d, J = 7.9 Hz, 1H), 6.75 (s, 1H), 3.18 (t, 2H), 2.86 (d, J = 11.0 Hz, 2H), 2.45 (s, 3H), 2.34 (t, J = 6.9 Hz, 2H), 2.23 (s, 3H), 2.20 (s, 3H), 1.92 – 1.82 (m, 2H), 1.69 – 1.54 (m, 4H), 1.32 (s, 1H), 1.16 (td, J = 10.5, 5.1 Hz, 2H), 0.87 (d, J = 6.4 Hz, 3H).

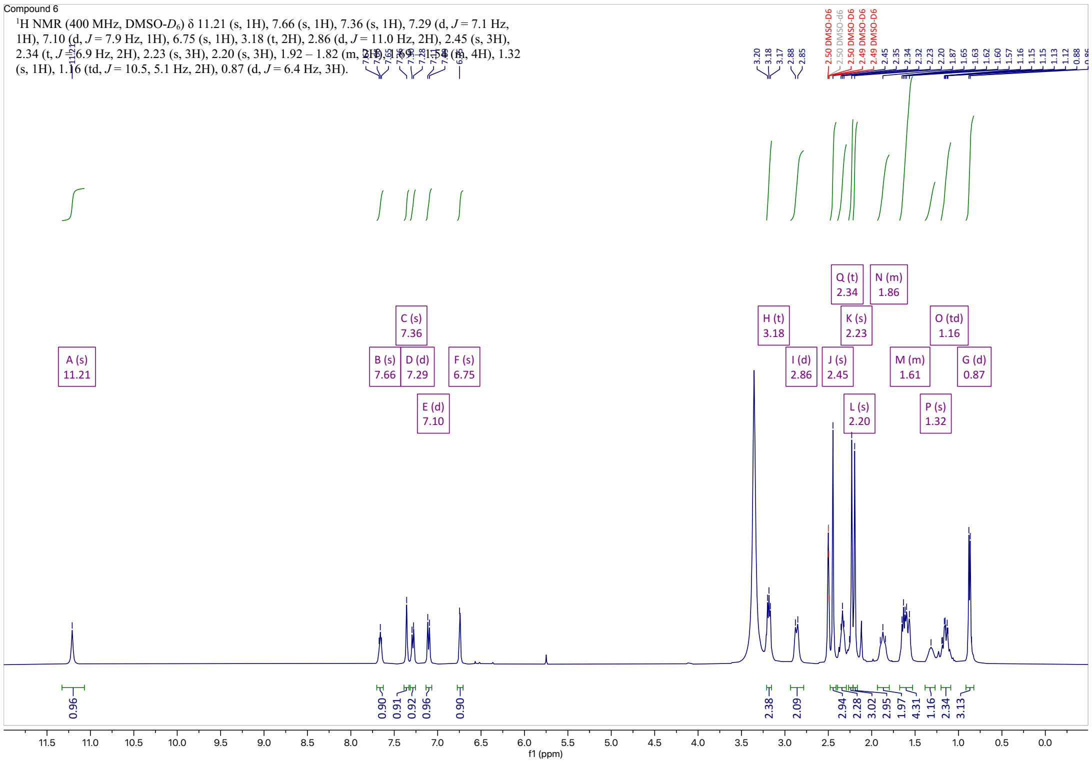

Compound 6

^{13}C NMR (101 MHz, DMSO-D_6) δ 164.91, 136.32, 133.56, 133.08, 130.00, 129.80, 128.79, 124.37, 120.63, 115.50, 103.74, 56.48, 53.46, 37.39, 33.92, 30.32, 26.44, 21.77, 19.56, 19.00, 12.57.

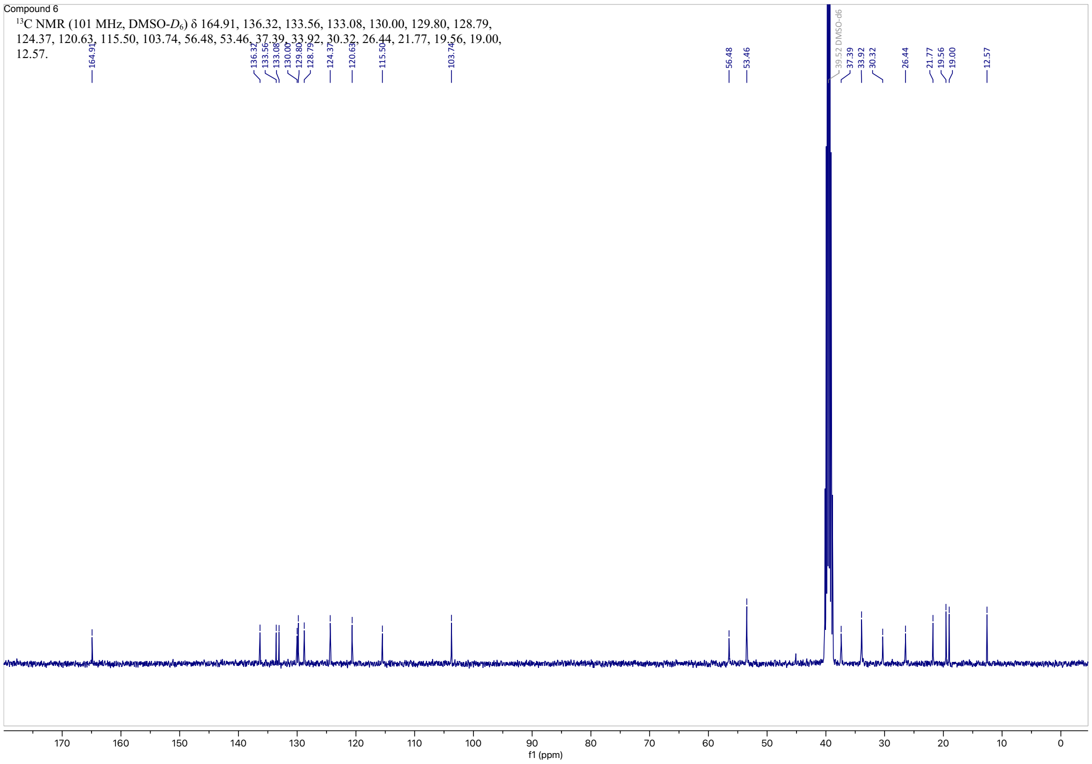

Compound 7

Compound 7

^{1}H NMR (400 MHz, DMSO-D_6) δ 11.53 (s, 1H), 7.84 (s, 1H), 7.40 (s, 1H), 7.32 (d, J = 7.9 Hz, 2H), 7.12 (d, J = 7.7 Hz, 1H), 6.80 (s, 1H), 5.75 (s, 1H), 3.22 (s, 2H), 2.84 (d, J = 26.7 Hz, 2H), 2.37 (s, 2H), 2.24 (s, 3H), 2.20 (s, 3H), 1.91 (s, 2H), 1.69 – 1.49 (m, 4H), 1.33 (s, 1H), 1.15 (q, J = 13.3 Hz, 2H), 0.88 (d, J = 6.3 Hz, 3H).

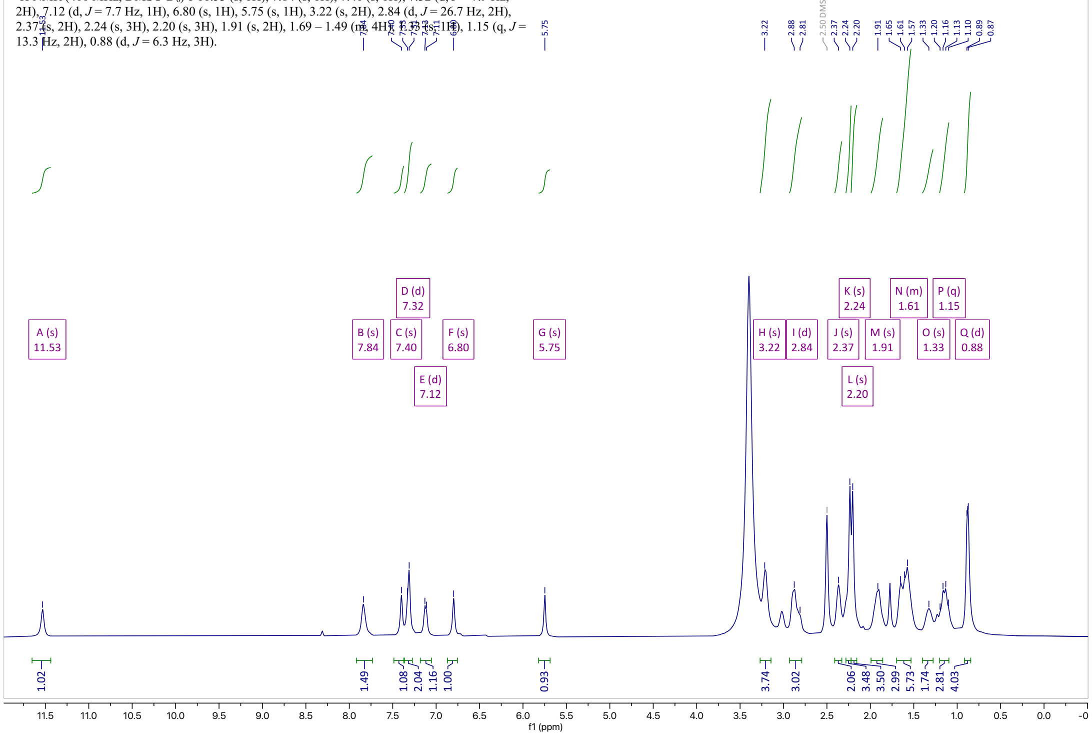

Compound 7

^{13}C NMR (101 MHz, DMSO-D_6) δ 163.85, 136.44, 134.09, 131.85, 129.92, 129.87, 124.80, 121.07, 121.01, 103.96, 56.11, 55.70, 54.91, 53.33, 37.25, 37.00, 33.74, 30.20, 26.48, 26.40, 22.64, 21.75, 19.55, 19.03.

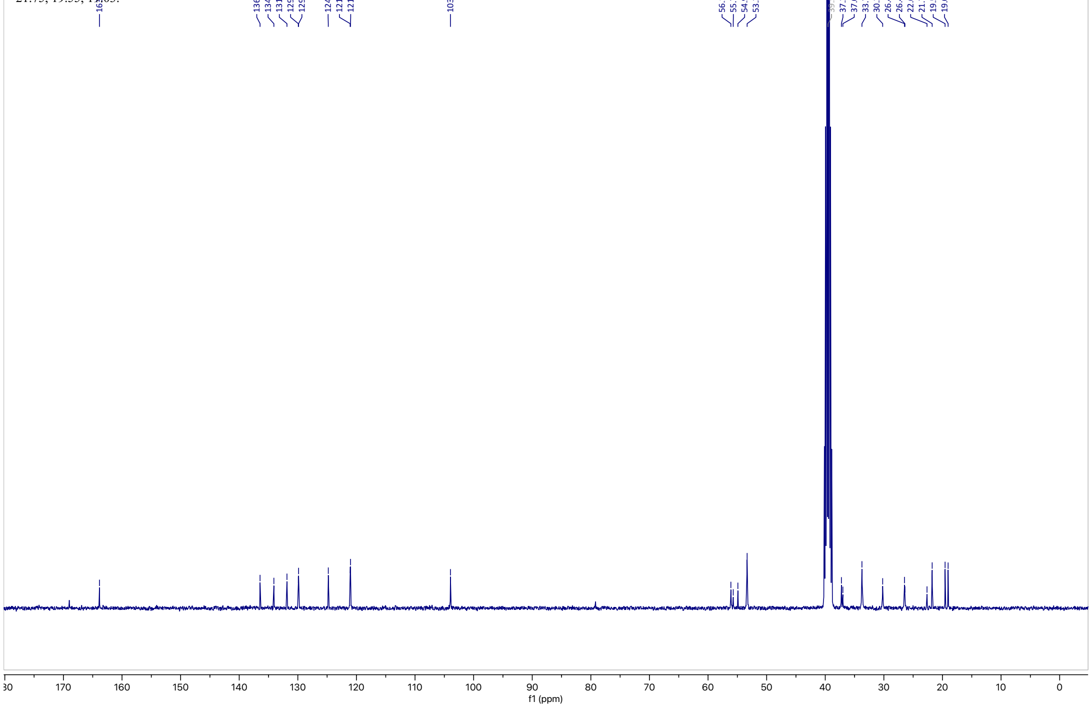

Compound 8

Compound 8

^{1}H NMR (400 MHz, CHLOROFORM-*D*) δ 8.74 (s, 1H), 7.26 (d, *J* = 2.5 Hz, 1H), 7.18 (d, *J* = 1.8 Hz, 1H), 7.19 – 7.12 (m, 2H), 7.10 (t, *J* = 6.2 Hz, 1H), 3.47 (q, *J* = 5.9 Hz, 2H), 3.07 – 2.97 (m, 2H), 2.57 (t, *J* = 6.6 Hz, 2H), 2.41 (s, 3H), 2.29 (s, 3H), 2.28 (s, 3H), 2.06 (t, *J* = 12.0 Hz, 2H), 1.87 – 1.78 (m, 2H), 1.67 (dt, *J* = 13.2, 5.0 Hz, 2H), 1.47 – 1.35 (m, 1H), 1.32 (dd, *J* = 12.5, 3.4 Hz, 2H), 0.93 (d, *J* = 6.3 Hz, 3H).

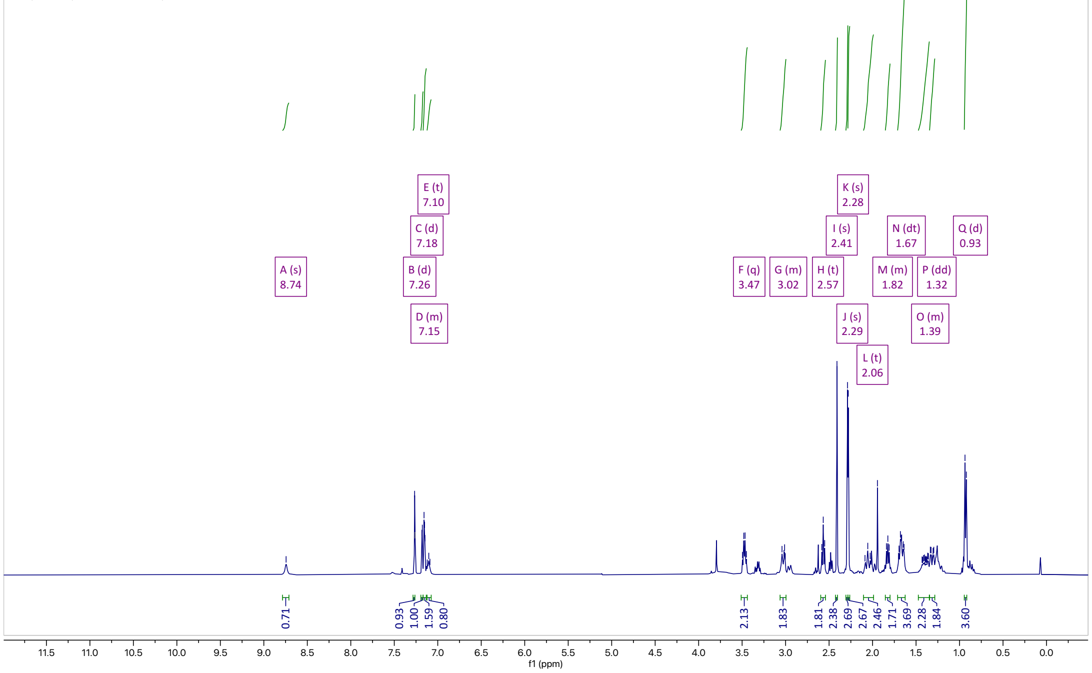

Compound 8

^{13}C NMR (101 MHz, CHLOROFORM-*D*) δ 166.50, 137.02, 135.37, 130.65, 130.50, 130.07, 128.70, 124.99, 120.49, 119.60, 115.13, 57.18, 53.95, 38.56, 34.40, 33.64, 25.56, 21.81, 19.99, 19.60, 11.50.

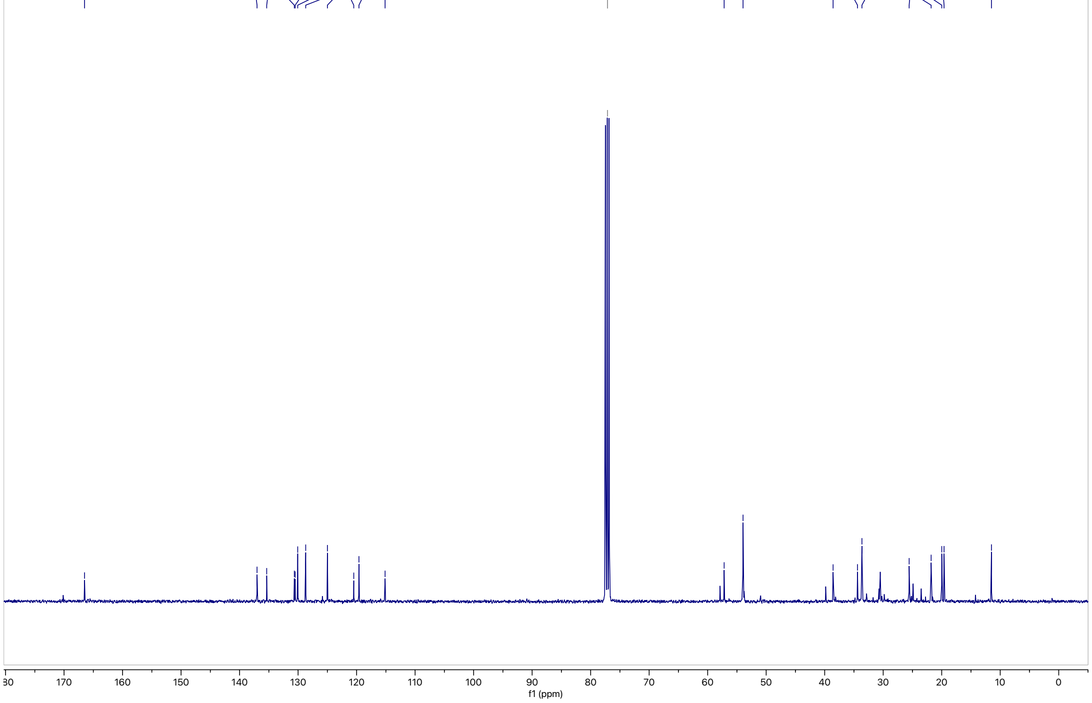

Compound 9

Compound 9

^{1}H NMR (400 MHz, DMSO) δ 11.26 – 11.20 (m, 1H), 7.89 (t, J = 6.0 Hz, 1H), 7.37 (d, J = 1.9 Hz, 1H), 7.30 (dd, J = 7.7, 2.0 Hz, 1H), 7.13 (d, J = 7.9 Hz, 1H), 6.78 (d, J = 2.6 Hz, 1H), 3.43 (s, 2H), 3.33 (s, 2H), 3.26 (q, J = 6.3 Hz, 2H), 3.04 (t, J = 7.5 Hz, 2H), 2.92 (q, J = 7.4 Hz, 2H), 2.24 (s, 3H), 2.21 (s, 3H), 1.85 (td, J = 16.2, 9.7 Hz, 4H), 1.60 (s, 1H), 1.41 – 1.25 (m, 2H), 1.17 (t, J = 7.4 Hz, 3H), 0.93 (d, J = 6.5 Hz, 3H).

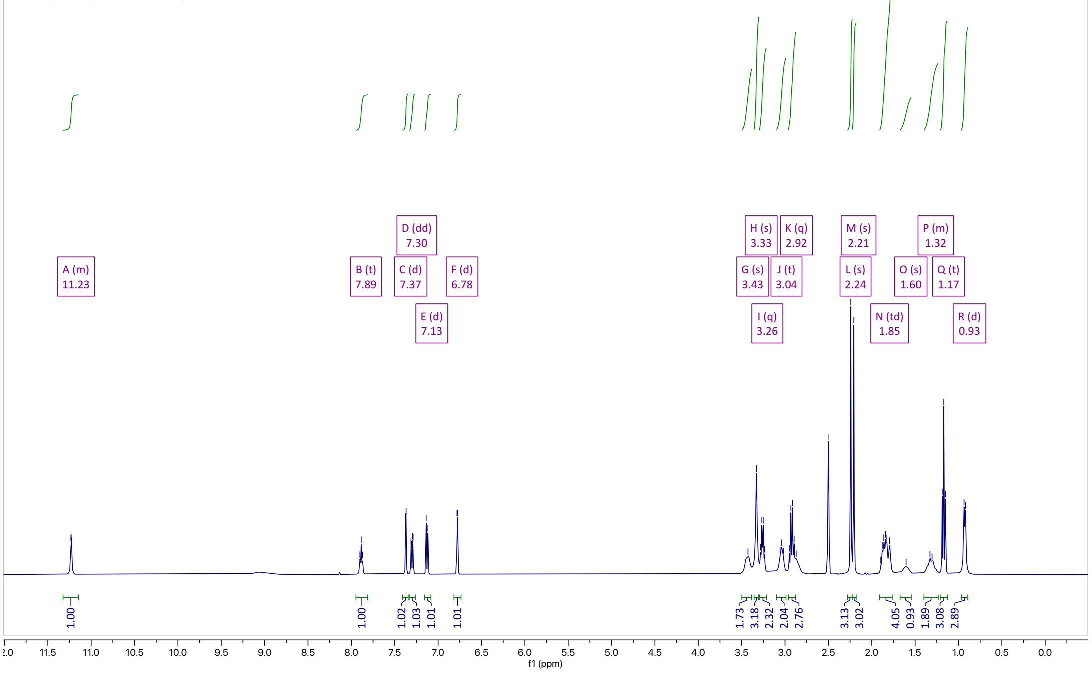

Compound 9

^{13}C NMR (101 MHz, DMSO) δ 165.62, 140.01, 136.36, 133.76, 129.89, 129.85, 129.08, 124.49, 120.81, 114.01, 103.87, 54.02, 51.99, 35.50, 31.14, 28.14, 24.62, 21.09, 19.63, 19.57, 19.02, 14.90.

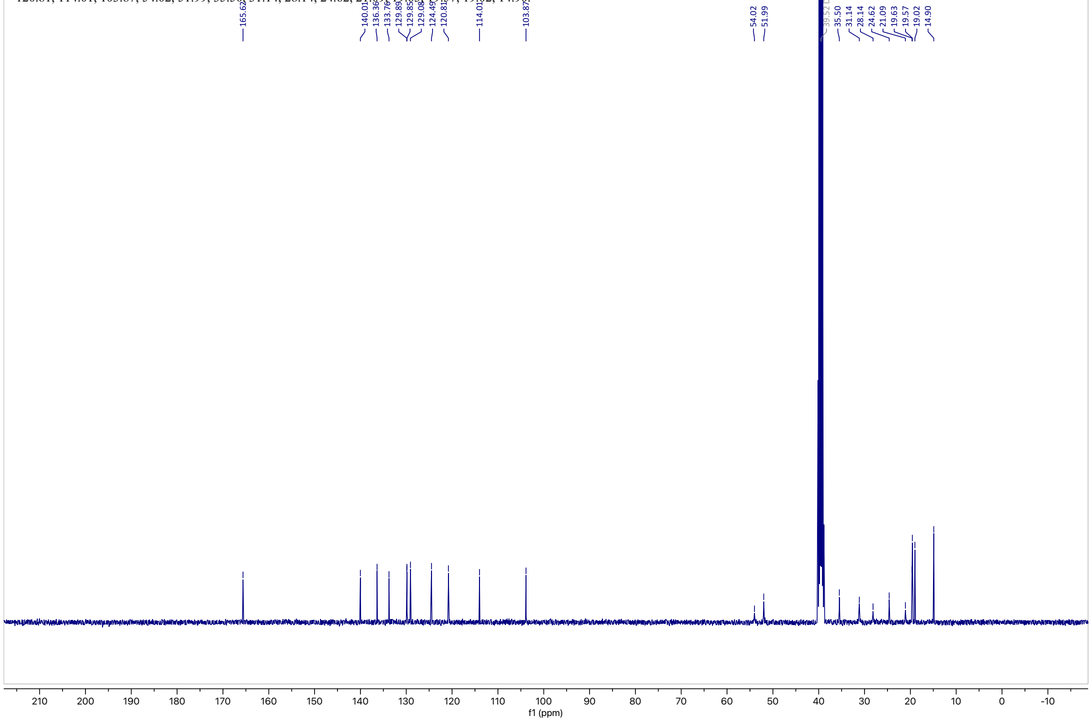

Compound 10

Compound 10

^{1}H NMR (400 MHz, CHLOROFORM-*D*) δ 9.78 (s, 1H), 8.09 (s, 1H), 7.36 (s, 1H), 7.32 (d, *J* = 7.9 Hz, 1H), 7.14 (d, *J* = 7.8 Hz, 1H), 6.70 (t, *J* = 3.1 Hz, 1H), 6.46 (t, *J* = 3.3 Hz, 1H), 3.53 (q, *J* = 5.6 Hz, 2H), 3.06 (d, *J* = 11.3 Hz, 2H), 2.66 (s, 1H), 2.59 (t, *J* = 6.1 Hz, 2H), 2.29 (s, 3H), 2.26 (s, 3H), 2.10 – 1.98 (m, 2H), 1.82 (t, *J* = 6.1 Hz, 2H), 1.71 (d, *J* = 11.5 Hz, 2H), 1.42 (d, *J* = 10.8 Hz, 2H), 0.98 (d, *J* = 5.3 Hz, 3H).

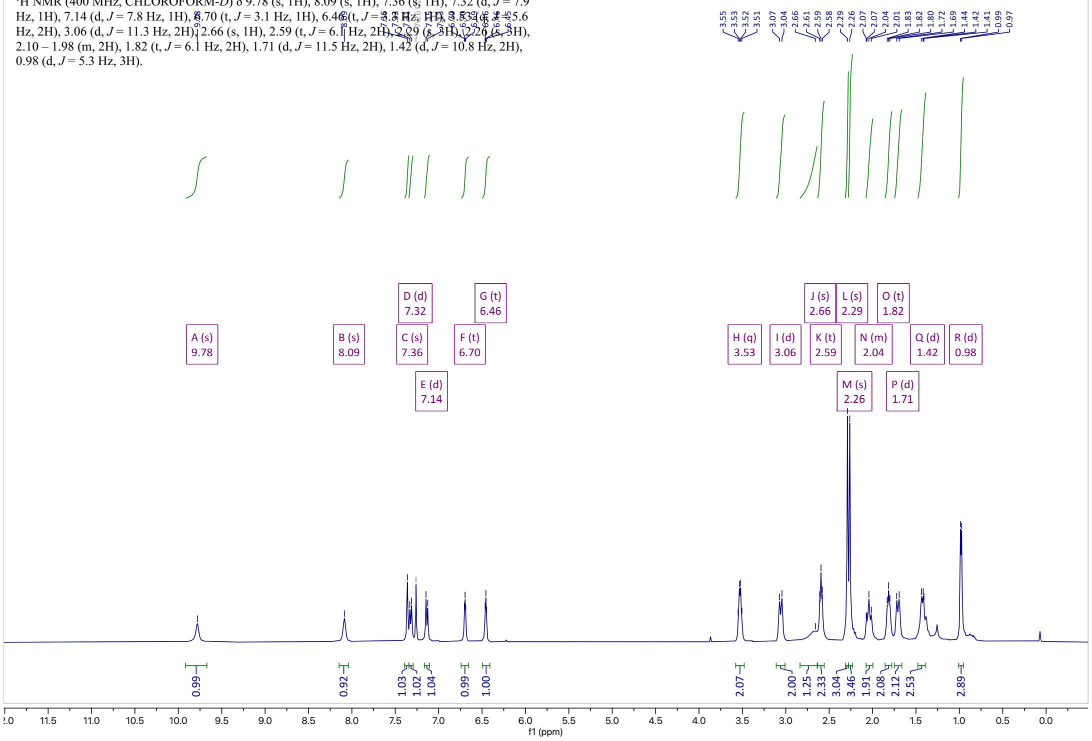

Compound 10

^{13}C NMR (101 MHz, CHLOROFORM-*D*) δ 161.39, 137.21, 135.87, 135.41, 130.29, 129.55, 126.67, 125.95, 122.09, 110.98, 106.67, 58.14, 54.16, 39.71, 33.92, 30.59, 24.70, 21.86, 20.00, 19.63.

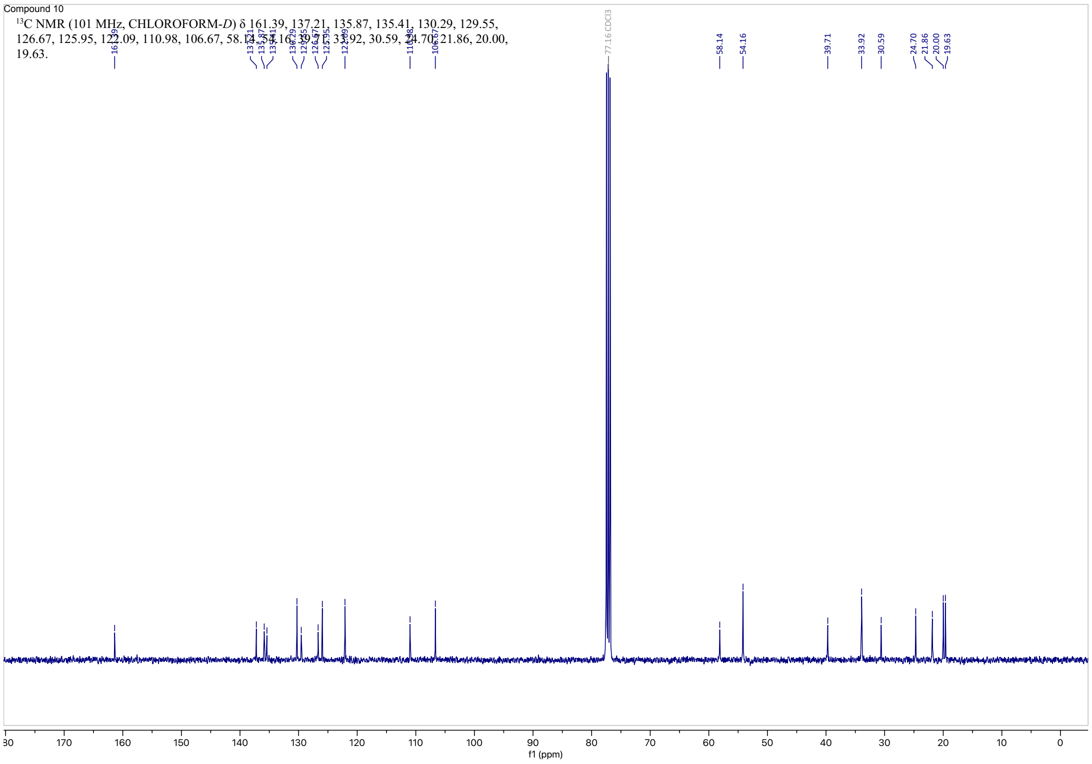

Compound 11

Compound 11

^{1}H NMR (400 MHz, CHLOROFORM-*D*) δ 10.02 (s, 1H), 8.28 (s, 1H), 7.26 (d, *J* = 12.9 Hz, 2H), 7.14 (s, 1H), 7.08 (d, *J* = 7.9 Hz, 1H), 6.86 (s, 1H), 3.52 (dt, *J* = 12.4, 5.9 Hz, 2H), 3.03 (d, *J* = 12.6 Hz, 4H), 2.53 (q, *J* = 5.7 Hz, 2H), 2.26 (s, 3H), 2.24 (s, 3H), 1.94 (d, *J* = 10.2 Hz, 2H), 1.73 (dt, *J* = 26.8, 9.1 Hz, 5H), 0.98 (d, *J* = 5.3 Hz, 3H).

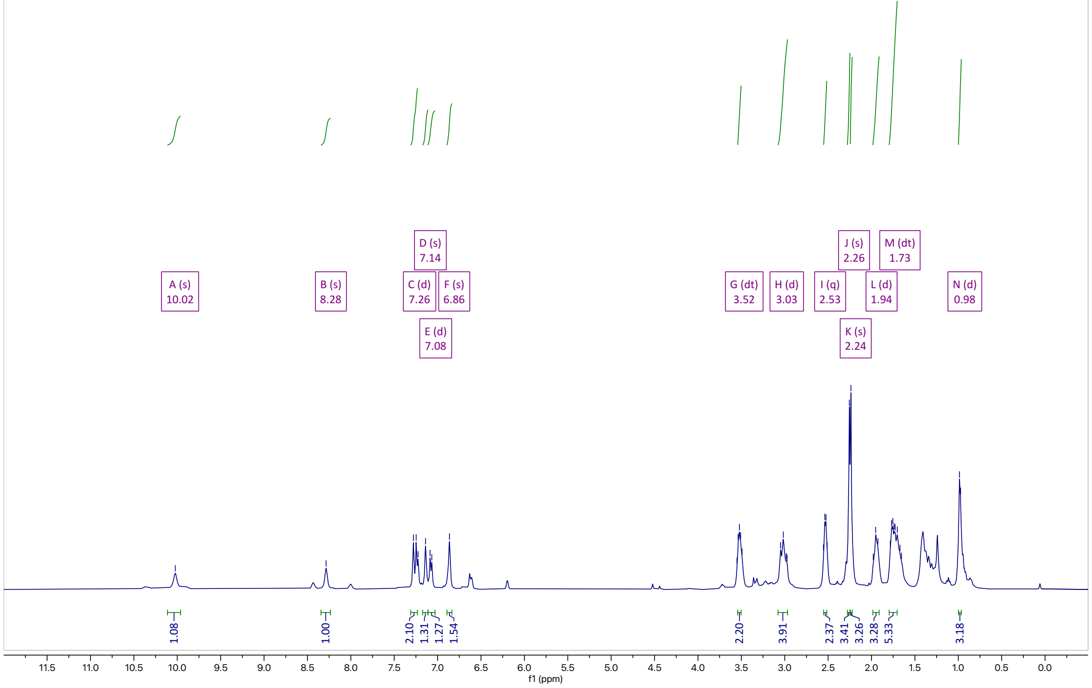

Compound 11

^{13}C NMR (101 MHz, CHLOROFORM-*D*) δ 161.33, 136.84, 134.33, 132.82, 130.04, 127.31, 126.63, 126.17, 122.78, 117.65, 106.38, 59.08, 54.47, 40.54, 34.61, 30.94, 24.46, 21.96, 19.96, 19.50.

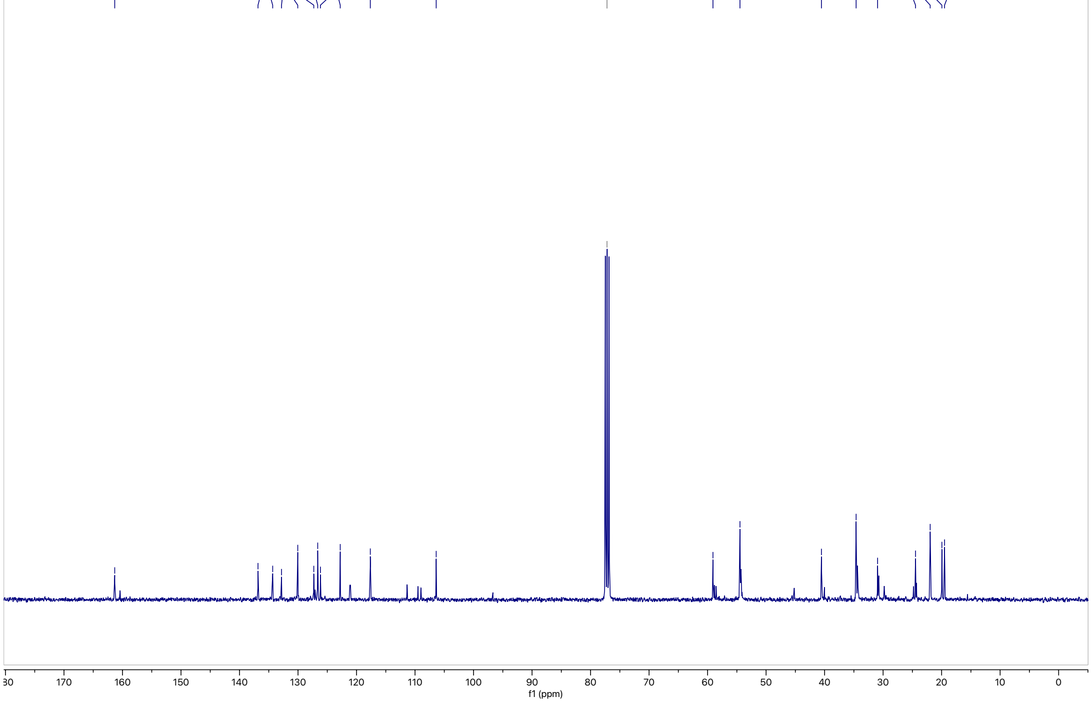

Compound 12

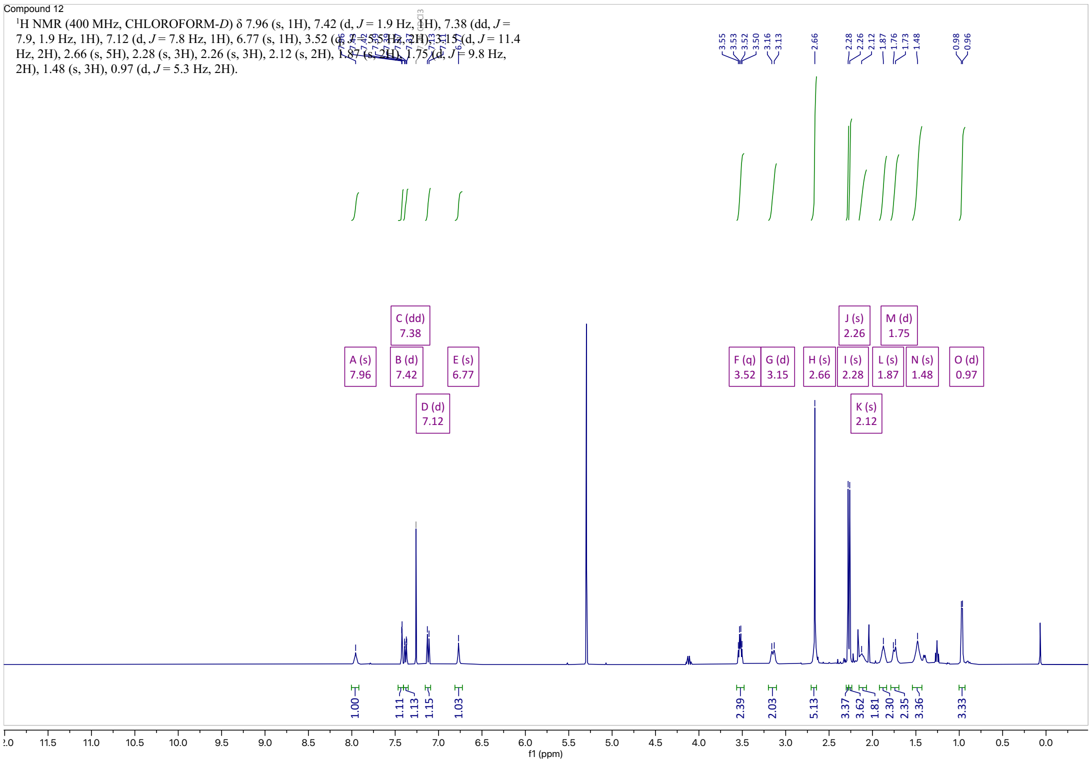
Compound 12
¹H NMR (400 MHz, CHLOROFORM-D) δ 7.96 (s, 1H), 7.42 (d, J = 1.9 Hz, 1H), 7.38 (dd, J = 7.9, 1.9 Hz, 1H), 7.12 (d, J = 7.8 Hz, 1H), 6.77 (s, 1H), 3.52 (q, J = 5.5 Hz, 2H), 3.15 (d, J = 11.4 Hz, 2H), 2.66 (s, 5H), 2.28 (s, 3H), 2.26 (s, 3H), 2.12 (s, 2H), 1.87 (s, 2H), 1.75 (d, J = 9.8 Hz, 2H), 1.48 (s, 3H), 0.97 (d, J = 5.3 Hz, 2H).
f1 (ppm)

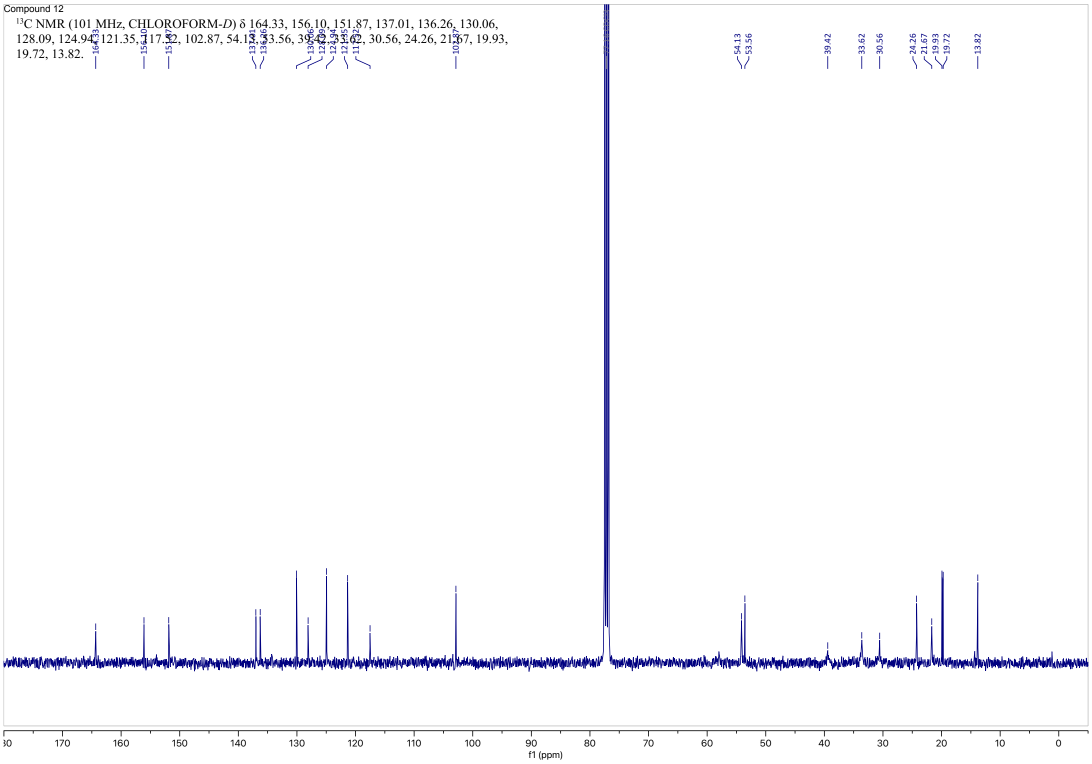
Compound 12
¹³C NMR (101 MHz, CHLOROFORM-D) δ 164.33, 156.10, 151.87, 137.01, 136.26, 130.06, 128.09, 124.94, 121.35, 117.52, 102.87, 54.13, 53.56, 39.42, 33.62, 30.56, 24.26, 21.67, 19.93, 19.72, 13.82.
f1 (ppm)

Compound 13

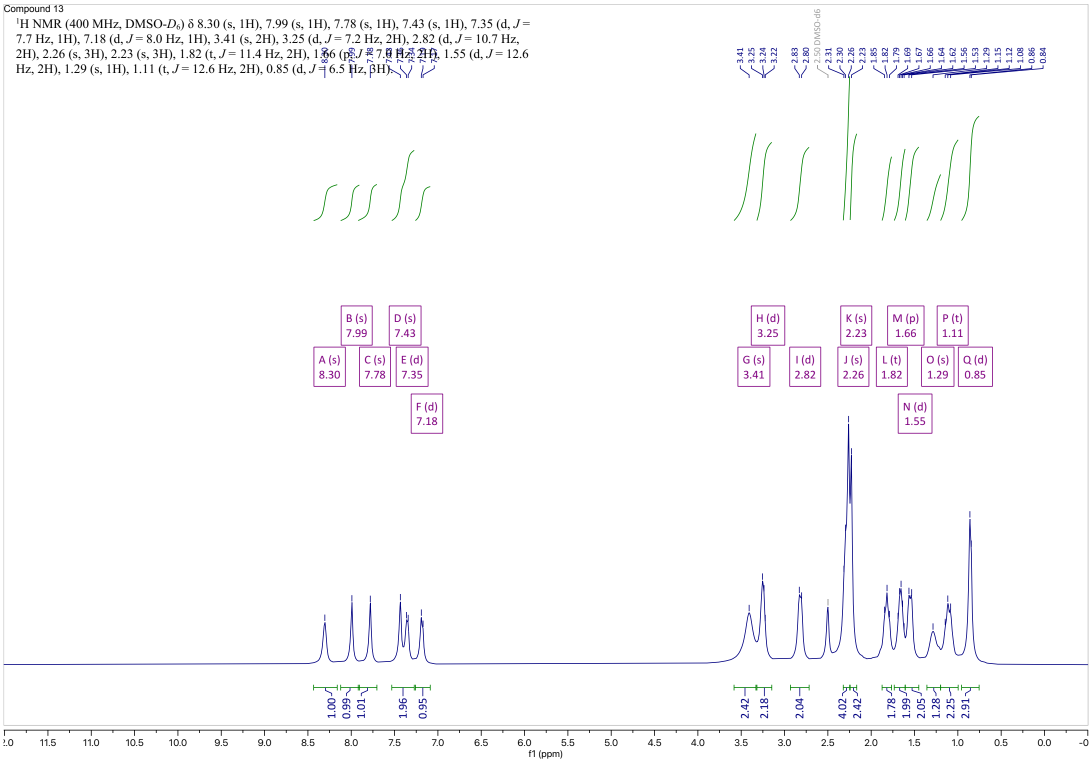
Compound 13
^{1}H NMR (400 MHz, DMSO-D_6) δ 8.30 (s, 1H), 7.99 (s, 1H), 7.78 (s, 1H), 7.43 (s, 1H), 7.35 (d, J = 7.7 Hz, 1H), 7.18 (d, J = 8.0 Hz, 1H), 3.41 (s, 2H), 3.25 (d, J = 7.2 Hz, 2H), 2.82 (d, J = 10.7 Hz, 2H), 2.26 (s, 3H), 2.23 (s, 3H), 1.82 (t, J = 11.4 Hz, 2H), 1.66 (p, J = 7.0 Hz, 2H), 1.55 (d, J = 12.6 Hz, 2H), 1.29 (s, 1H), 1.11 (t, J = 12.6 Hz, 2H), 0.85 (d, J = 6.5 Hz, 3H).
f1 (ppm)

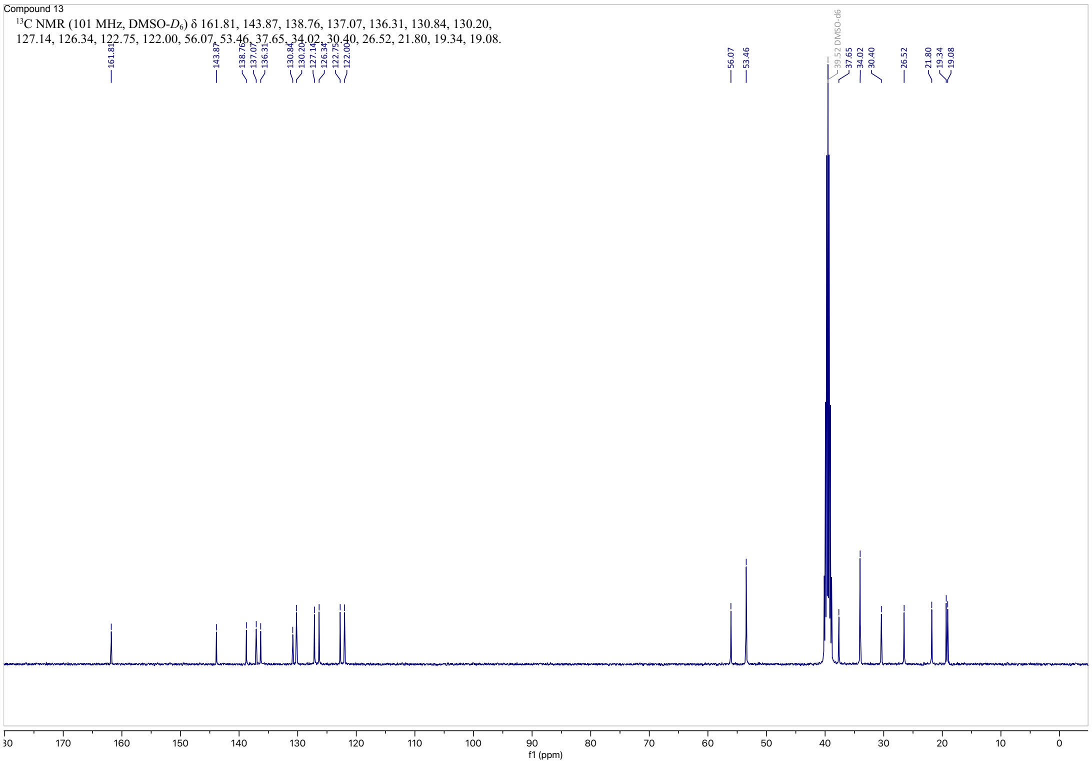
Compound 13
^{13}C NMR (101 MHz, DMSO-D_6) δ 161.81, 143.87, 138.76, 137.07, 136.31, 130.84, 130.20, 127.14, 126.34, 122.75, 122.00, 56.07, 53.46, 37.65, 34.02, 30.40, 26.52, 21.80, 19.34, 19.08.
f1 (ppm)

Compound 14

Compound 14

^{1}H NMR (400 MHz, CHLOROFORM-*D*) δ 8.63 (s, 1H), 8.34 (s, 1H), 7.76 (s, 1H), 7.68 (dd, *J* = 7.8, 1.9 Hz, 1H), 7.20 (d, *J* = 7.9 Hz, 1H), 3.59 (q, *J* = 5.5 Hz, 2H), 3.24 (d, *J* = 11.5 Hz, 2H), 2.79 (s, 2H), 2.32 (s, 3H), 2.30 (s, 3H), 2.00 – 1.93 (m, 2H), 1.84 – 1.72 (m, 3H), 1.60 (d, *J* = 25.9 Hz, 3H), 1.05 – 0.99 (m, 4H).

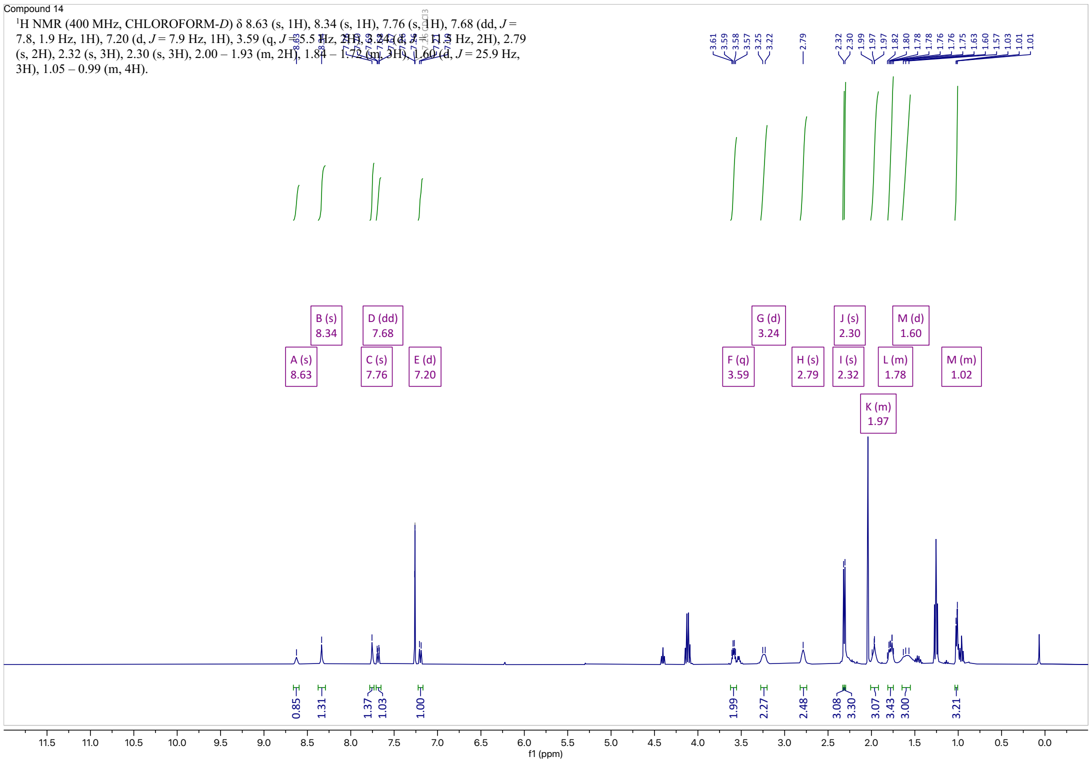

Compound 14

^{13}C NMR (101 MHz, CHLOROFORM-*D*) δ 171.30, 161.14, 144.87, 140.11, 137.60, 133.54, 131.05, 130.42, 127.94, 124.46, 72.20, 60.54, 30.91, 23.73, 21.19, 19.97, 19.84, 14.34, 13.82.

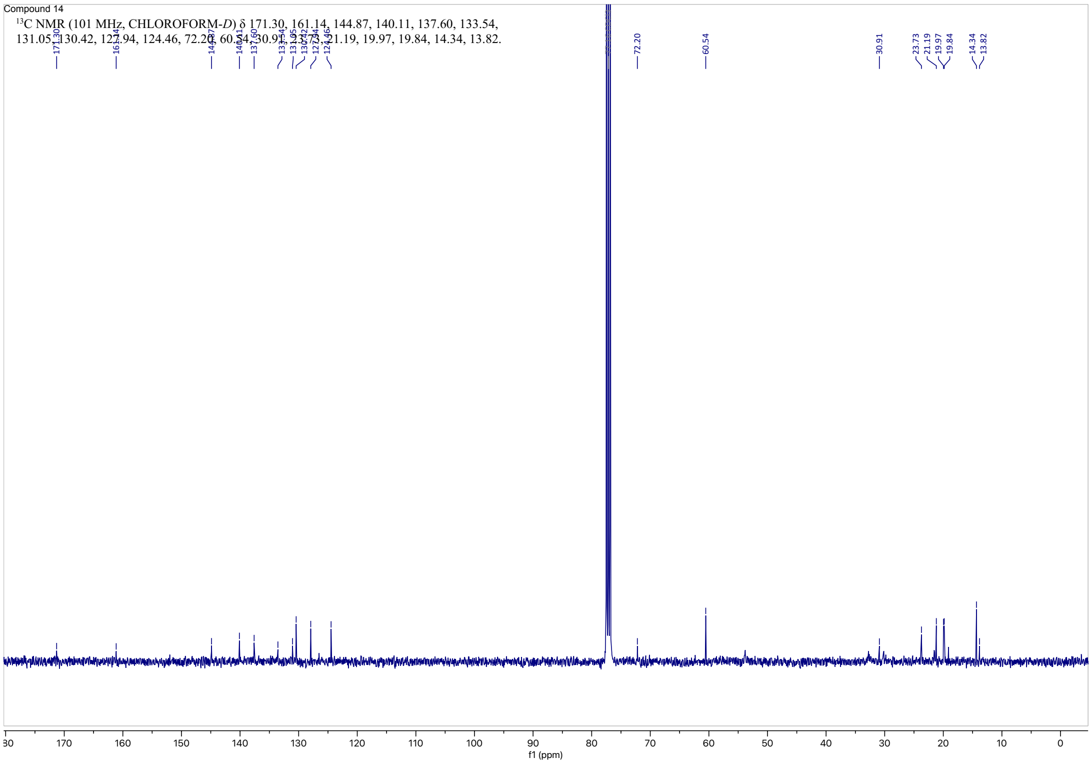

Compound 15

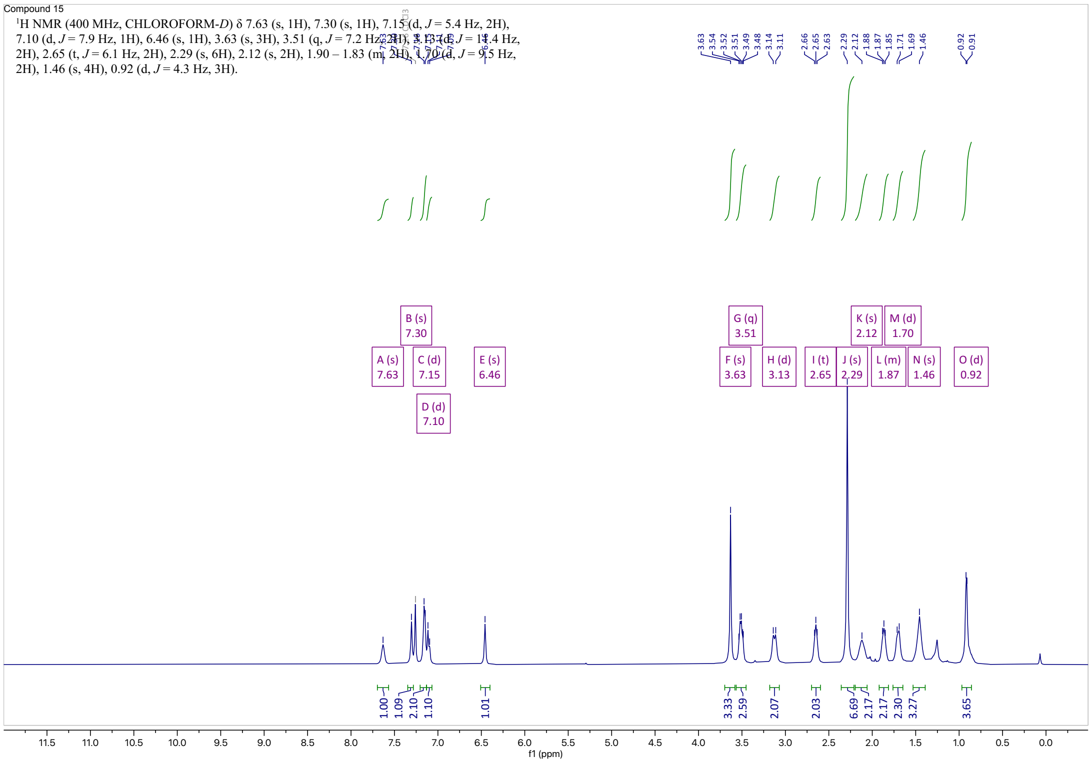
Compound 15
^{1}H NMR (400 MHz, CHLOROFORM-*D*) δ 7.63 (s, 1H), 7.30 (s, 1H), 7.15 (d, *J* = 5.4 Hz, 2H), 7.10 (d, *J* = 7.9 Hz, 1H), 6.46 (s, 1H), 3.63 (s, 3H), 3.51 (q, *J* = 7.2 Hz, 2H), 3.13 (d, *J* = 1[illegible].4 Hz, 2H), 2.65 (t, *J* = 6.1 Hz, 2H), 2.29 (s, 6H), 2.12 (s, 2H), 1.90 – 1.83 (m, 2H), 1.70 (d, *J* = 9.5 Hz, 2H), 1.46 (s, 4H), 0.92 (d, *J* = 4.3 Hz, 3H).
f1 (ppm)

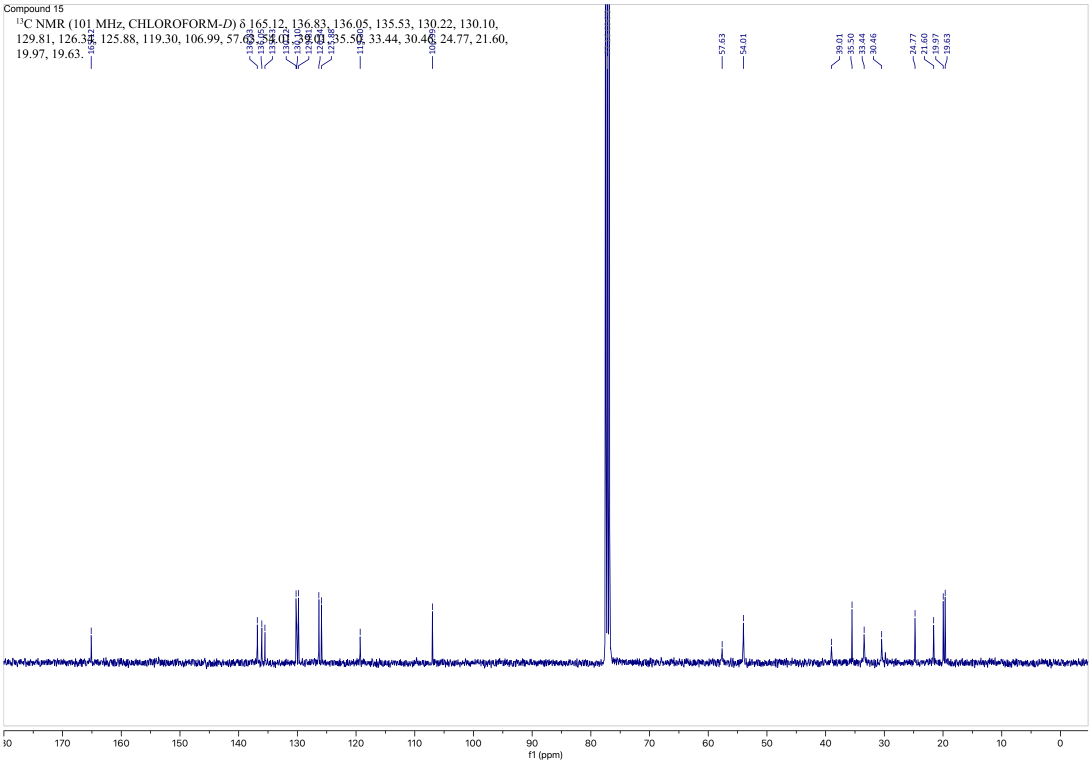
Compound 15
^{13}C NMR (101 MHz, CHLOROFORM-*D*) δ 165.12, 136.83, 136.05, 135.53, 130.22, 130.10, 129.81, 126.34, 125.88, 119.30, 106.99, 57.63, 54.01, 39.01, 35.50, 33.44, 30.46, 24.77, 21.60, 19.97, 19.63.
f1 (ppm)

Compound 16

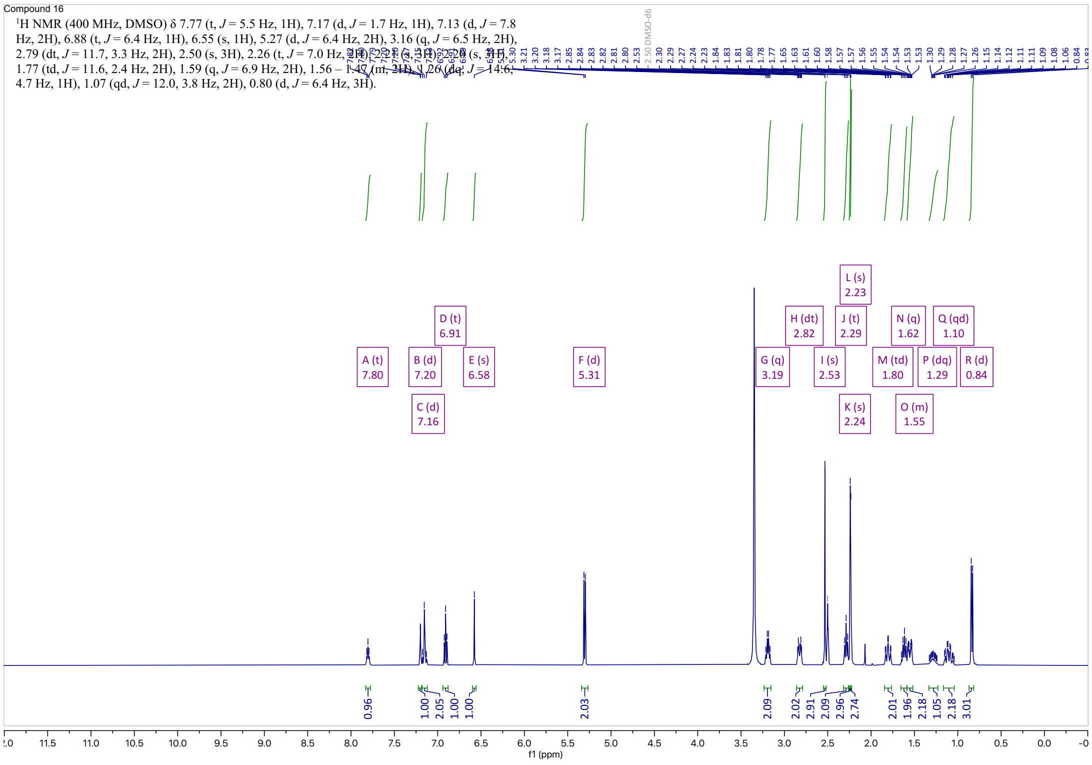
Compound 16
¹H NMR (400 MHz, DMSO) δ 7.77 (t, J = 5.5 Hz, 1H), 7.17 (d, J = 1.7 Hz, 1H), 7.13 (d, J = 7.8 Hz, 2H), 6.88 (t, J = 6.4 Hz, 1H), 6.55 (s, 1H), 5.27 (d, J = 6.4 Hz, 2H), 3.16 (q, J = 6.5 Hz, 2H), 2.79 (dt, J = 11.7, 3.3 Hz, 2H), 2.50 (s, 3H), 2.26 (t, J = 7.0 Hz, 2H), 2.21 (s, 3H), 2.20 (s, 3H), 1.77 (td, J = 11.6, 2.4 Hz, 2H), 1.59 (q, J = 6.9 Hz, 2H), 1.56 – 1.47 (m, 2H), 1.26 (dq, J = 14.6, 4.7 Hz, 1H), 1.07 (qd, J = 12.0, 3.8 Hz, 2H), 0.80 (d, J = 6.4 Hz, 3H).
f1 (ppm)

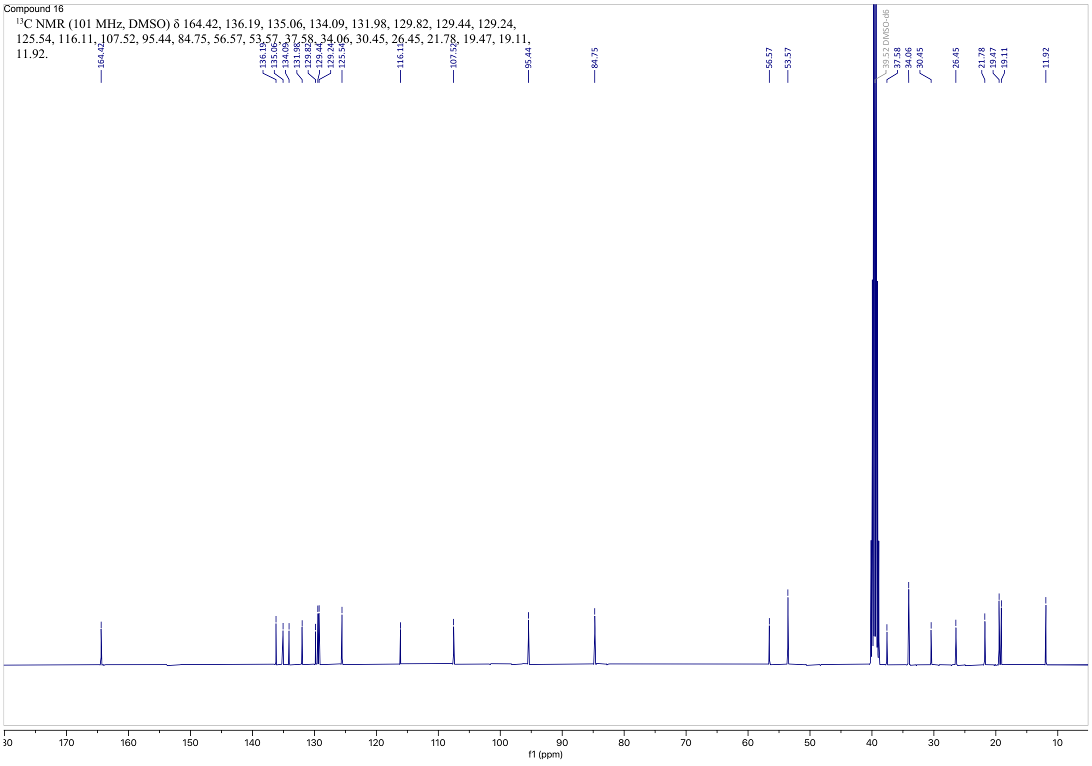
Compound 16
¹³C NMR (101 MHz, DMSO) δ 164.42, 136.19, 135.06, 134.09, 131.98, 129.82, 129.44, 129.24, 125.54, 116.11, 107.52, 95.44, 84.75, 56.57, 53.57, 37.58, 34.06, 30.45, 26.45, 21.78, 19.47, 19.11, 11.92.
f1 (ppm)

Compound 17

Compound 17

^{1}H NMR (400 MHz, DMSO) δ 7.70 (t, J = 5.5 Hz, 1H), 7.18 (d, J = 7.7 Hz, 1H), 7.08 (d, J = 1.8 Hz, 1H), 7.01 (dd, J = 7.6, 1.9 Hz, 1H), 6.32 (s, 1H), 4.57 (p, J = 9.2 Hz, 1H), 3.17 (q, J = 6.6 Hz, 2H), 2.84 (dt, J = 11.8, 3.4 Hz, 2H), 2.60 (s, 3H), 2.31 (t, J = 6.9 Hz, 2H), 2.24 (s, 6H), 1.99 – 1.69 (m, 8H), 1.66 – 1.44 (m, 6H), 1.29 (tdd, J = 10.9, 7.2, 4.1 Hz, 1H), 1.10 (qd, J = 12.0, 3.7 Hz, 2H), 0.82 (d, J = 6.5 Hz, 3H).

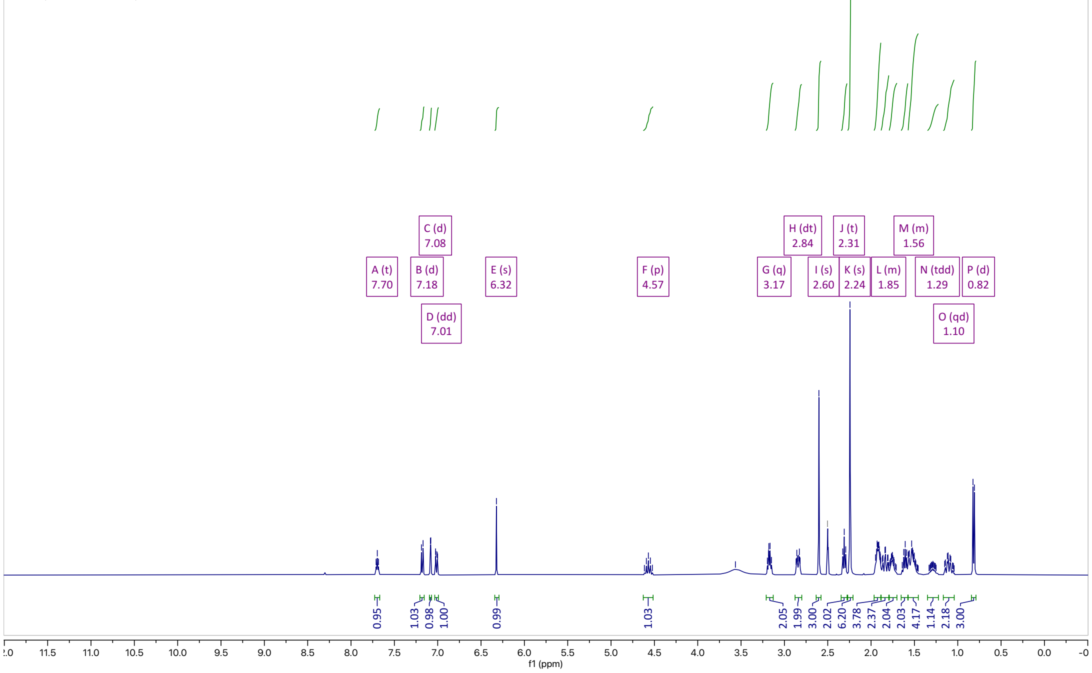

Compound 17

^{13}C NMR (101 MHz, DMSO) δ 164.96, 136.29, 135.42, 133.41, 132.25, 131.07, 130.38, 129.48, 126.56, 115.81, 106.90, 56.54, 53.47, 37.52, 33.87, 31.11, 30.32, 26.32, 24.74, 21.67, 19.42, 19.09, 12.85.

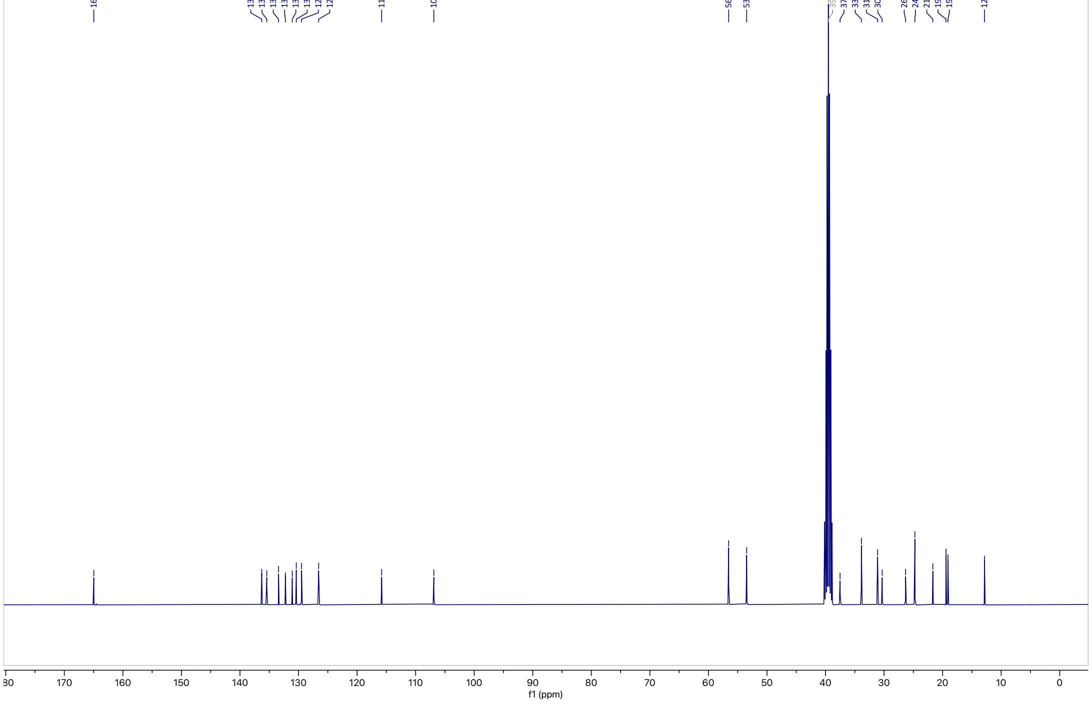

Compound 18

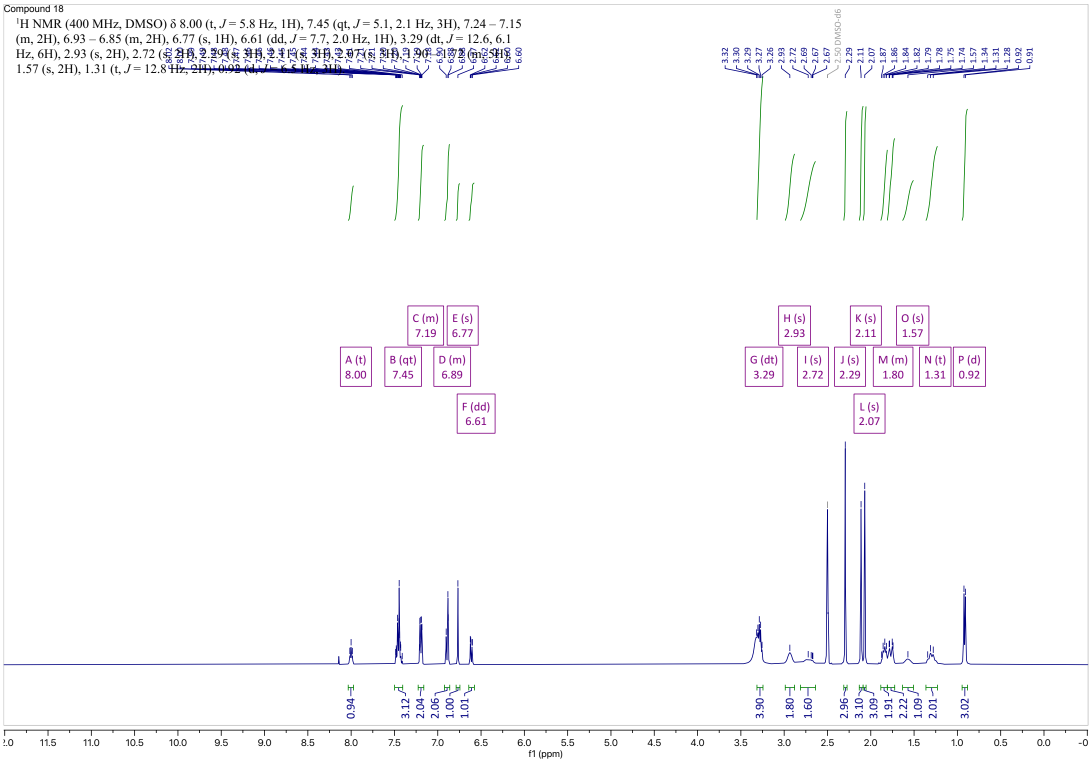
Compound 18
^{1}H NMR (400 MHz, DMSO) δ 8.00 (t, J = 5.8 Hz, 1H), 7.45 (qt, J = 5.1, 2.1 Hz, 3H), 7.24 – 7.15 (m, 2H), 6.93 – 6.85 (m, 2H), 6.77 (s, 1H), 6.61 (dd, J = 7.7, 2.0 Hz, 1H), 3.29 (dt, J = 12.6, 6.1 Hz, 6H), 2.93 (s, 2H), 2.72 (s, 2H), 2.29 (s, 3H), 2.11 (s, 3H), 2.07 (s, 3H), 1.90 – 1.72 (m, 5H), 1.57 (s, 2H), 1.31 (t, J = 12.8 Hz, 2H), 0.92 (d, J = 6.5 Hz, 3H).
f1 (ppm)

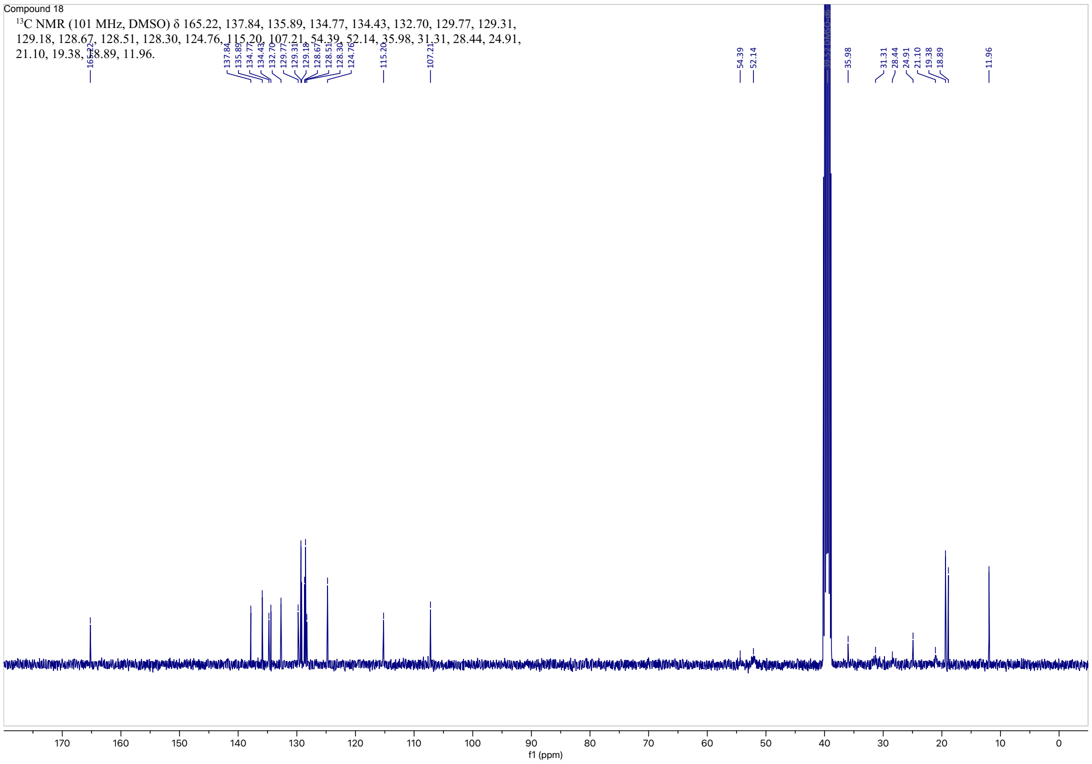
Compound 18
^{13}C NMR (101 MHz, DMSO) δ 165.22, 137.84, 135.89, 134.77, 134.43, 132.70, 129.77, 129.31, 129.18, 128.67, 128.51, 128.30, 124.76, 115.20, 107.21, 54.39, 52.14, 35.98, 31.31, 28.44, 24.91, 21.10, 19.38, 18.89, 11.96.
f1 (ppm)

Compound 19

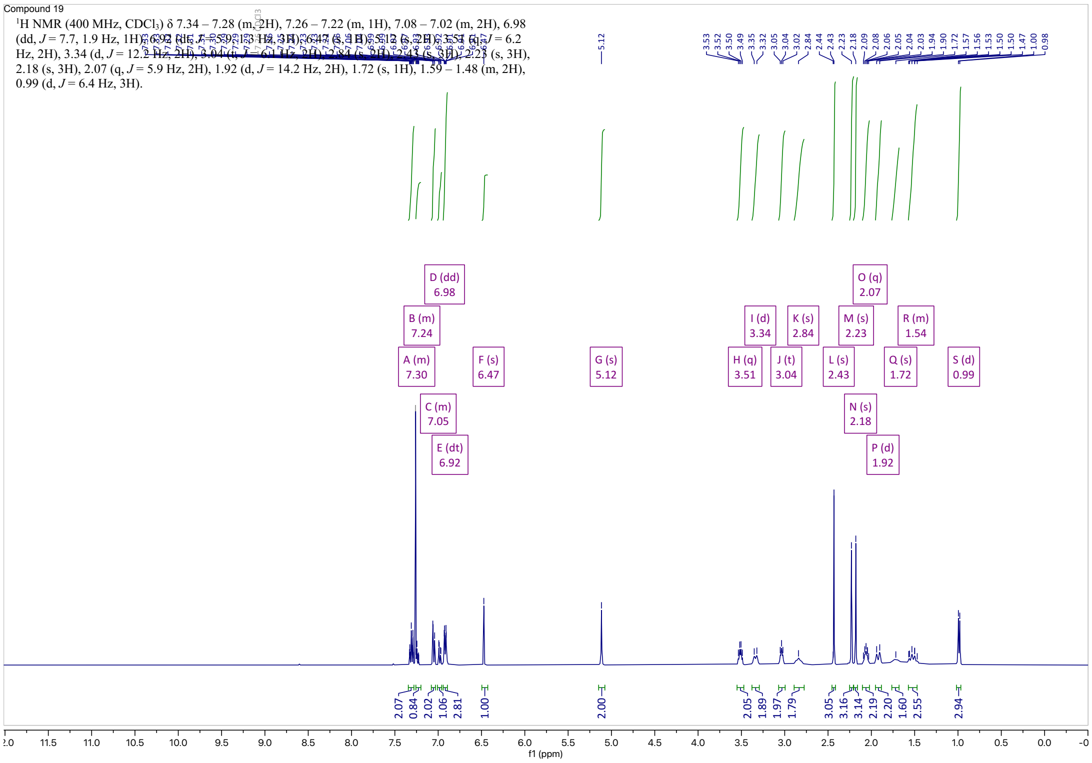
Compound 19
¹H NMR (400 MHz, CDCl₃) δ 7.34 – 7.28 (m, 2H), 7.26 – 7.22 (m, 1H), 7.08 – 7.02 (m, 2H), 6.98 (dd, J = 7.7, 1.9 Hz, 1H), 6.92 (dt, J = 5.9, 1.3 Hz, 3H), 6.47 (s, 1H), 5.12 (s, 2H), 3.51 (q, J = 6.2 Hz, 2H), 3.34 (d, J = 12.2 Hz, 2H), 3.04 (t, J = 6.1 Hz, 2H), 2.84 (s, 2H), 2.43 (s, 3H), 2.23 (s, 3H), 2.18 (s, 3H), 2.07 (q, J = 5.9 Hz, 2H), 1.92 (d, J = 14.2 Hz, 2H), 1.72 (s, 1H), 1.59 – 1.48 (m, 2H), 0.99 (d, J = 6.4 Hz, 3H).
f1 (ppm)

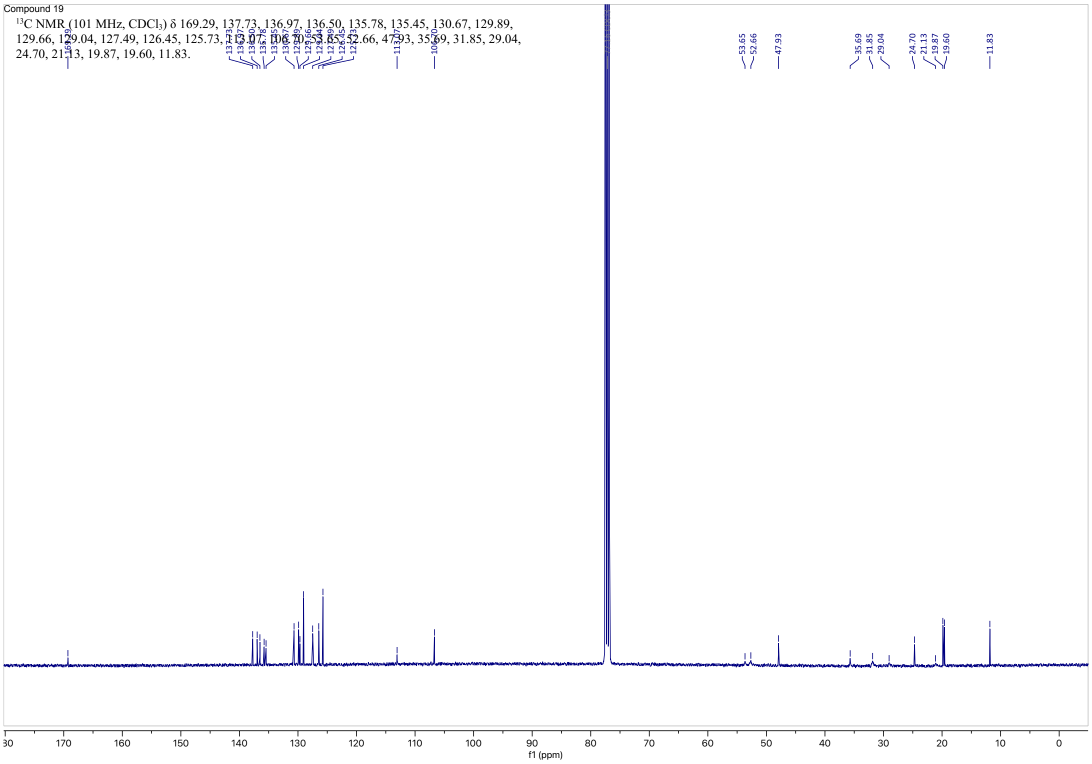
Compound 19
¹³C NMR (101 MHz, CDCl₃) δ 169.29, 137.73, 136.97, 136.50, 135.78, 135.45, 130.67, 129.89, 129.66, 129.04, 127.49, 126.45, 125.73, 113.07, 106.70, 53.65, 52.66, 47.93, 35.69, 31.85, 29.04, 24.70, 21.13, 19.87, 19.60, 11.83.
f1 (ppm)

Compound 20

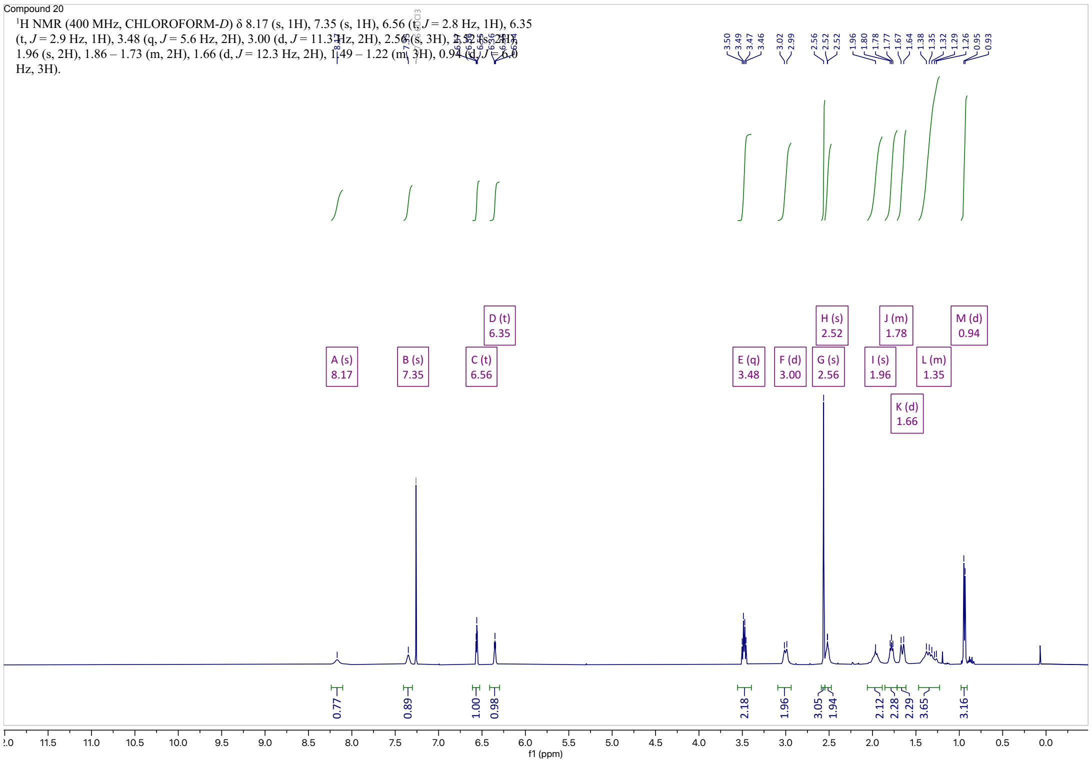
Compound 20
^{1}H NMR (400 MHz, CHLOROFORM-*D*) δ 8.17 (s, 1H), 7.35 (s, 1H), 6.56 (t, *J* = 2.8 Hz, 1H), 6.35 (t, *J* = 2.9 Hz, 1H), 3.48 (q, *J* = 5.6 Hz, 2H), 3.00 (d, *J* = 11.3 Hz, 2H), 2.56 (s, 3H), 2.52 (s, 2H), 1.96 (s, 2H), 1.86 – 1.73 (m, 2H), 1.66 (d, *J* = 12.3 Hz, 2H), 1.49 – 1.22 (m, 3H), 0.94 (d, *J* = 6.0 Hz, 3H).
A (s) 8.17
B (s) 7.35
C (t) 6.56
D (t) 6.35
E (q) 3.48
F (d) 3.00
G (s) 2.56
H (s) 2.52
I (s) 1.96
J (m) 1.78
K (d) 1.66
L (m) 1.35
M (d) 0.94
0.77
0.89
1.00
0.98
2.18
1.96
3.05
1.94
2.12
2.28
2.29
3.65
3.16
f1 (ppm)

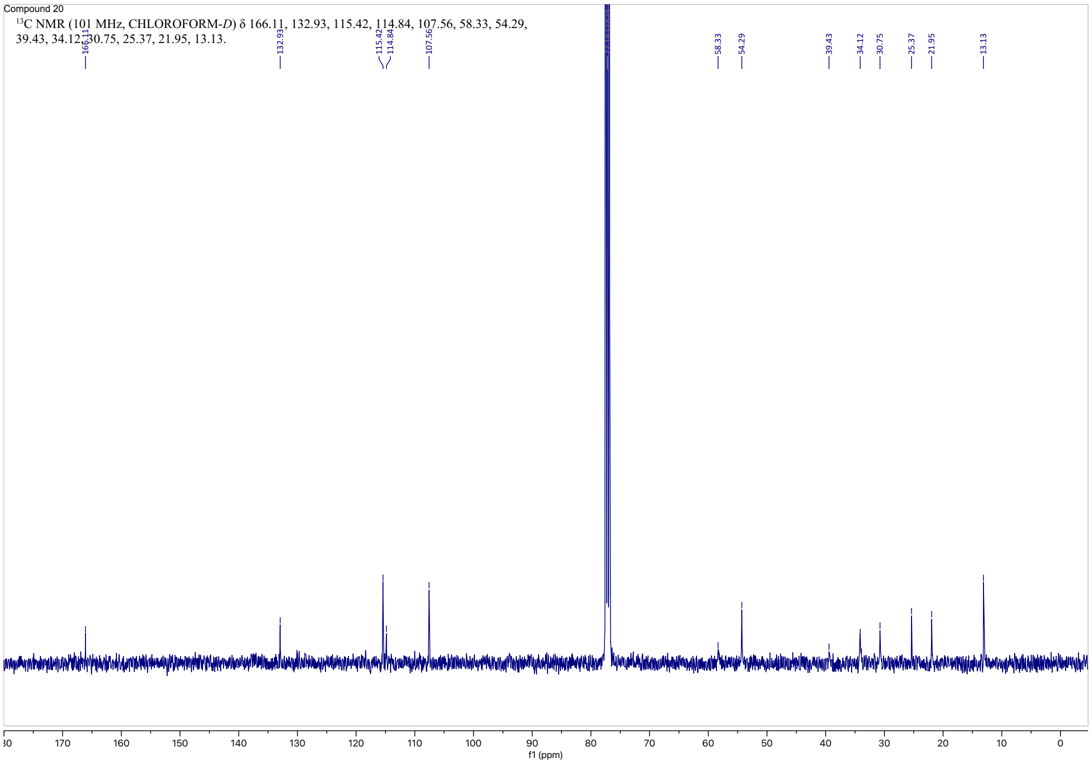
Compound 20
^{13}C NMR (101 MHz, CHLOROFORM-*D*) δ 166.11, 132.93, 115.42, 114.84, 107.56, 58.33, 54.29, 39.43, 34.12, 30.75, 25.37, 21.95, 13.13.
f1 (ppm)

Compound 21

Compound 21

^{1}H NMR (400 MHz, $CDCl_3$) δ 8.60 (s, 1H), 7.11 – 7.03 (m, 1H), 6.10 (d, J = 2.7 Hz, 1H), 3.42 (q, J = 6.0 Hz, 2H), 3.31 (d, J = 11.9 Hz, 2H), 2.89 (t, J = 6.9 Hz, 2H), 2.60 – 2.48 (m, 1H), 2.47 (d, J = 1.3 Hz, 3H), 1.95 (dt, J = 15.5, 7.3 Hz, 4H), 1.77 (dd, J = 13.8, 9.1 Hz, 4H), 1.71 – 1.62 (m, 2H), 1.60 – 1.46 (m, 4H), 1.30 (t, J = 10.0 Hz, 4H), 1.23 – 1.12 (m, 1H), 0.97 (d, J = 5.3 Hz, 3H).

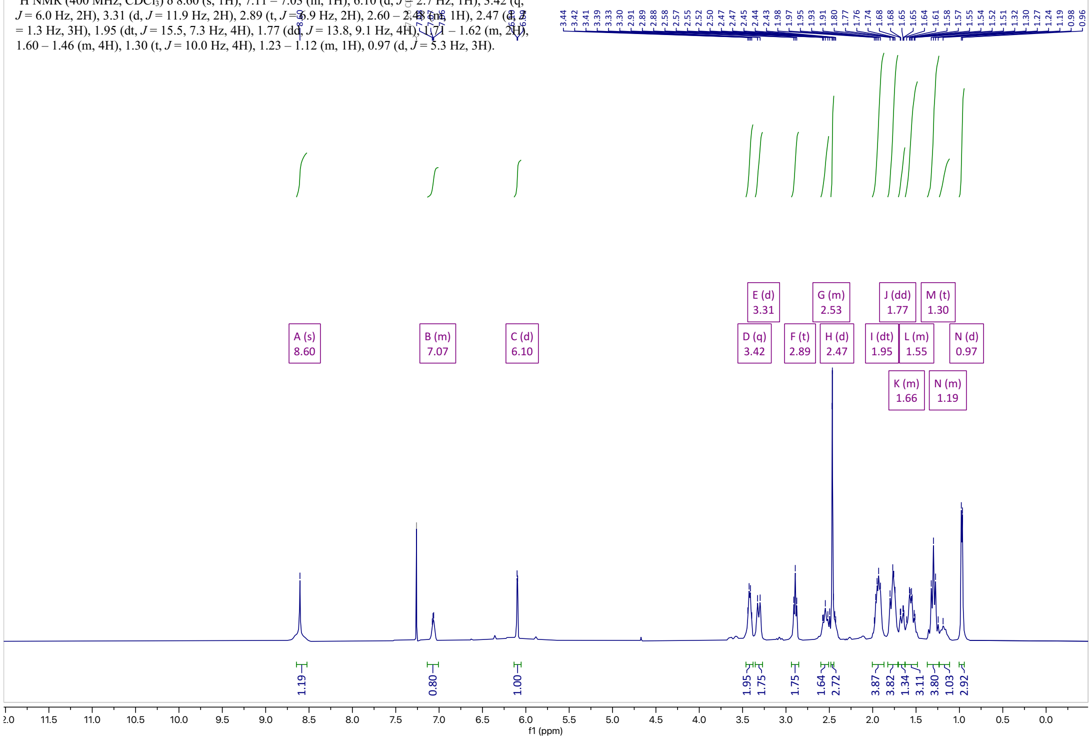

Compound 21

^{13}C NMR (101 MHz, $CDCl_3$) δ 168.91, 167.66, 136.58, 132.07, 113.02, 102.04, 54.60, 52.64, 36.55, 36.48, 33.10, 31.49, 29.36, 26.29, 26.11, 24.58, 21.09, 13.15.

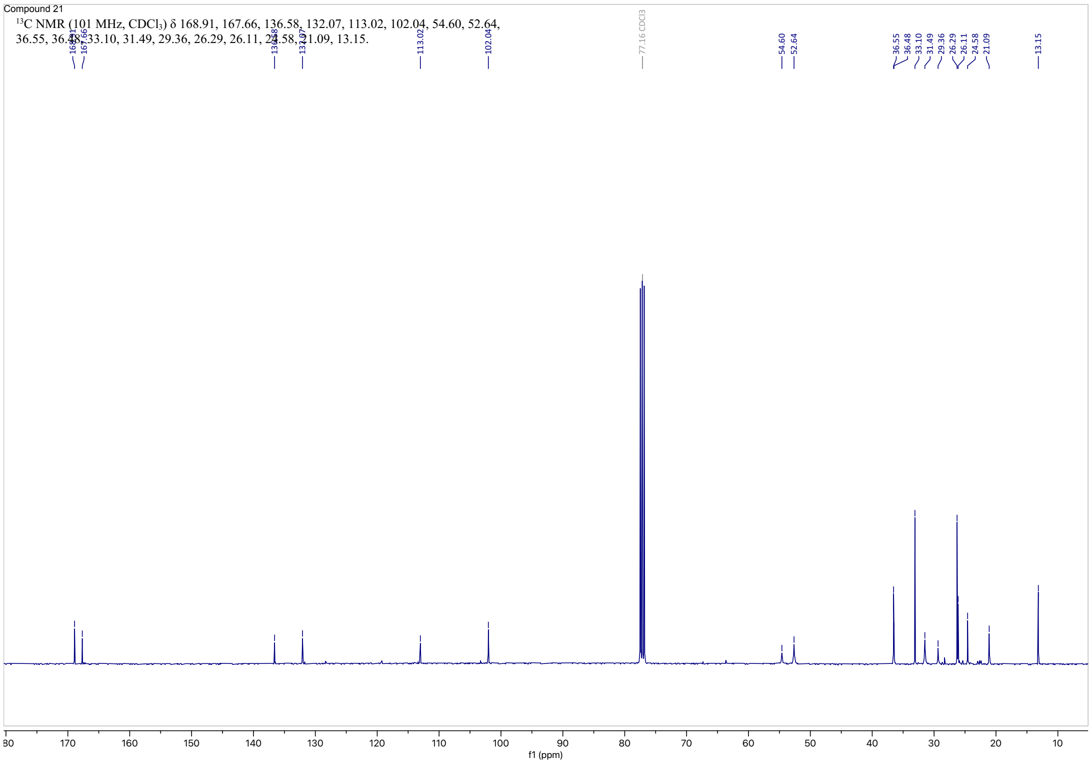

Compound 22

Compound 22

^{1}H NMR (400 MHz, DMSO-D_6) δ 11.32 (s, 1H), 7.70 (t, J = 5.4 Hz, 1H), 7.57 (d, J = 7.6 Hz, 2H), 7.35 (t, J = 7.3 Hz, 2H), 7.15 (t, J = 7.3 Hz, 1H), 6.83 (s, 1H), 3.20 (q, J = 7.4 Hz, 2H), 2.88 (d, J = 10.8 Hz, 2H), 2.47 (s, 3H), 2.37 (d, J = 9.3 Hz, 2H), 1.90 (s, 2H), 1.65 (t, J = 6.9 Hz, 2H), 1.59 (d, J = 13.5 Hz, 3H), 1.33 (s, 1H), 1.17 (dd, J = 17.5, 8.0 Hz, 2H), 0.87 (d, J = 6.3 Hz, 3H).

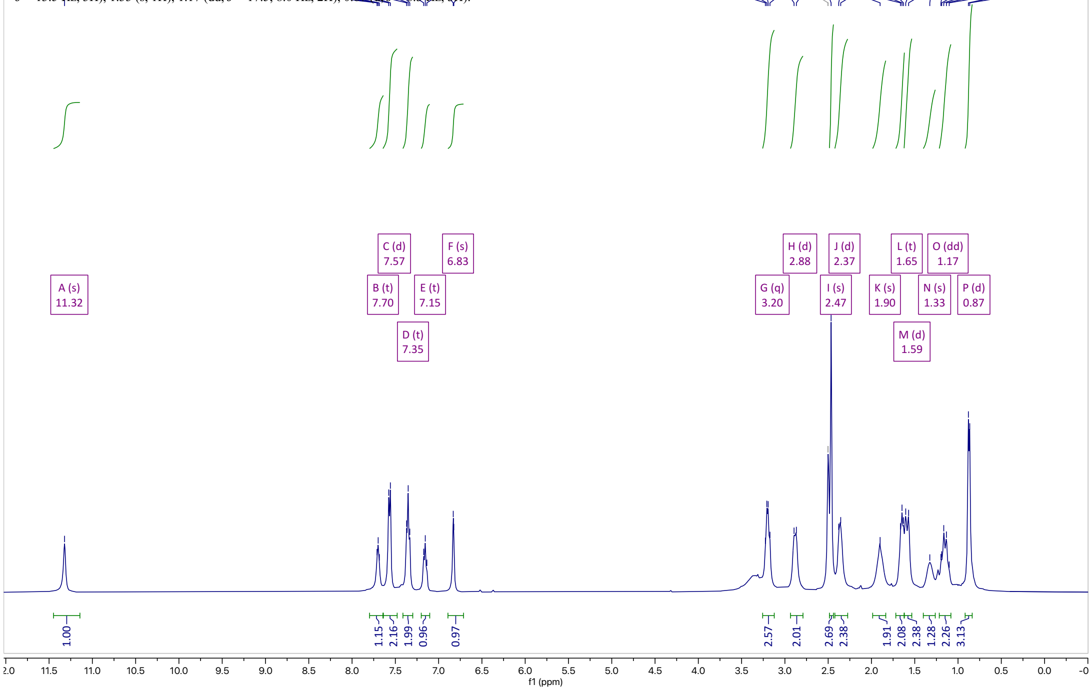

Compound 22

^{13}C NMR (101 MHz, DMSO-D_6) δ 164.84, 133.57, 132.33, 128.73, 128.57, 125.61, 123.10, 115.72, 104.52, 56.35, 53.40, 37.32, 33.81, 30.25, 26.40, 21.72, 12.59.

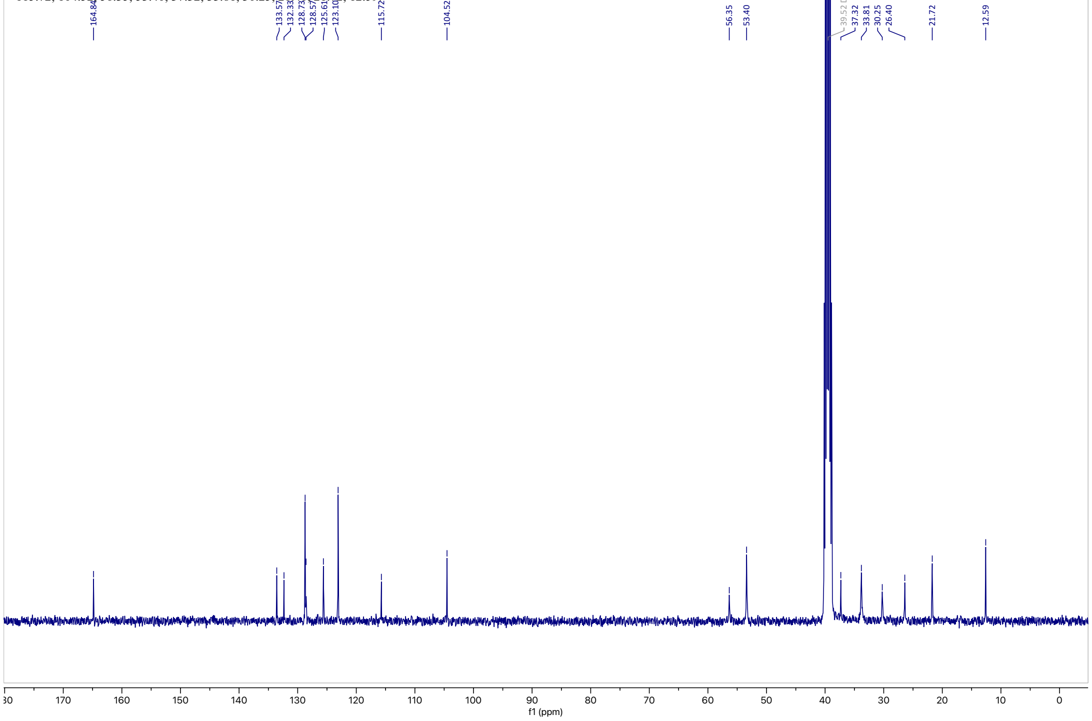

Compound 23

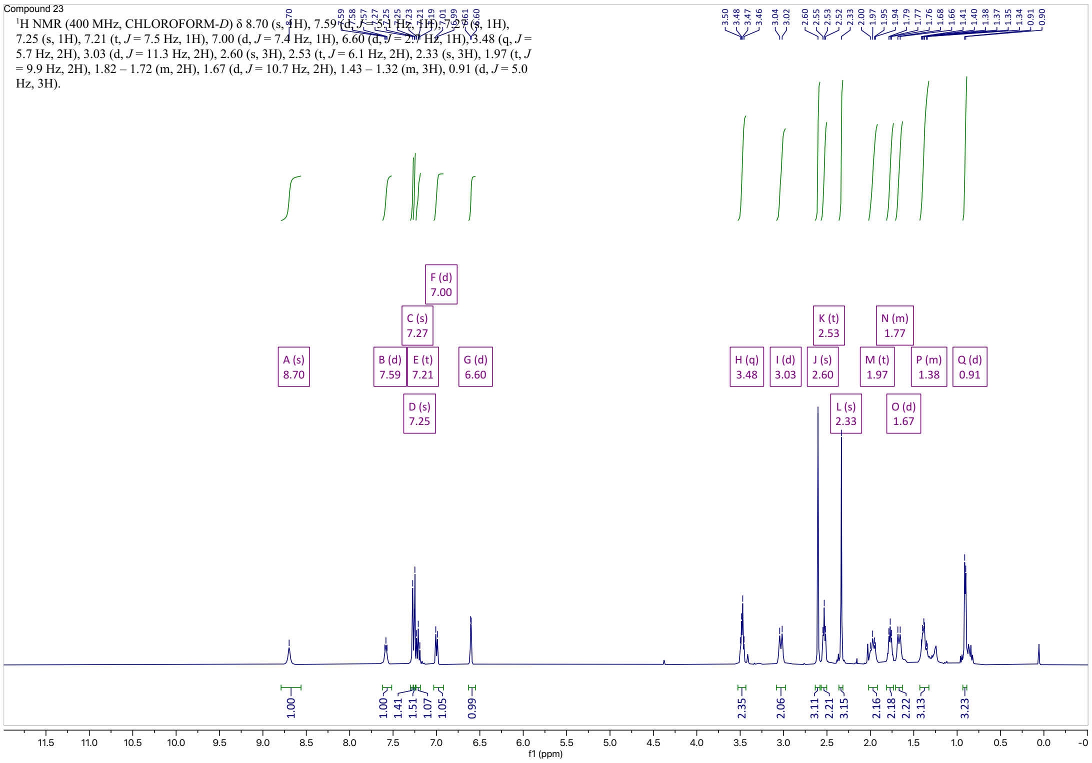
Compound 23
1H NMR (400 MHz, CHLOROFORM-D) δ 8.70 (s, 1H), 7.59 (d, J = 5.1 Hz, 1H), 7.27 (s, 1H), 7.25 (s, 1H), 7.21 (t, J = 7.5 Hz, 1H), 7.00 (d, J = 7.4 Hz, 1H), 6.60 (d, J = 2.7 Hz, 1H), 3.48 (q, J = 5.7 Hz, 2H), 3.03 (d, J = 11.3 Hz, 2H), 2.60 (s, 3H), 2.53 (t, J = 6.1 Hz, 2H), 2.33 (s, 3H), 1.97 (t, J = 9.9 Hz, 2H), 1.82 – 1.72 (m, 2H), 1.67 (d, J = 10.7 Hz, 2H), 1.43 – 1.32 (m, 3H), 0.91 (d, J = 5.0 Hz, 3H).
A (s) 8.70
B (d) 7.59
C (s) 7.27
D (s) 7.25
E (t) 7.21
F (d) 7.00
G (d) 6.60
H (q) 3.48
I (d) 3.03
J (s) 2.60
K (t) 2.53
L (s) 2.33
M (t) 1.97
N (m) 1.77
O (d) 1.67
P (m) 1.38
Q (d) 0.91
f1 (ppm)

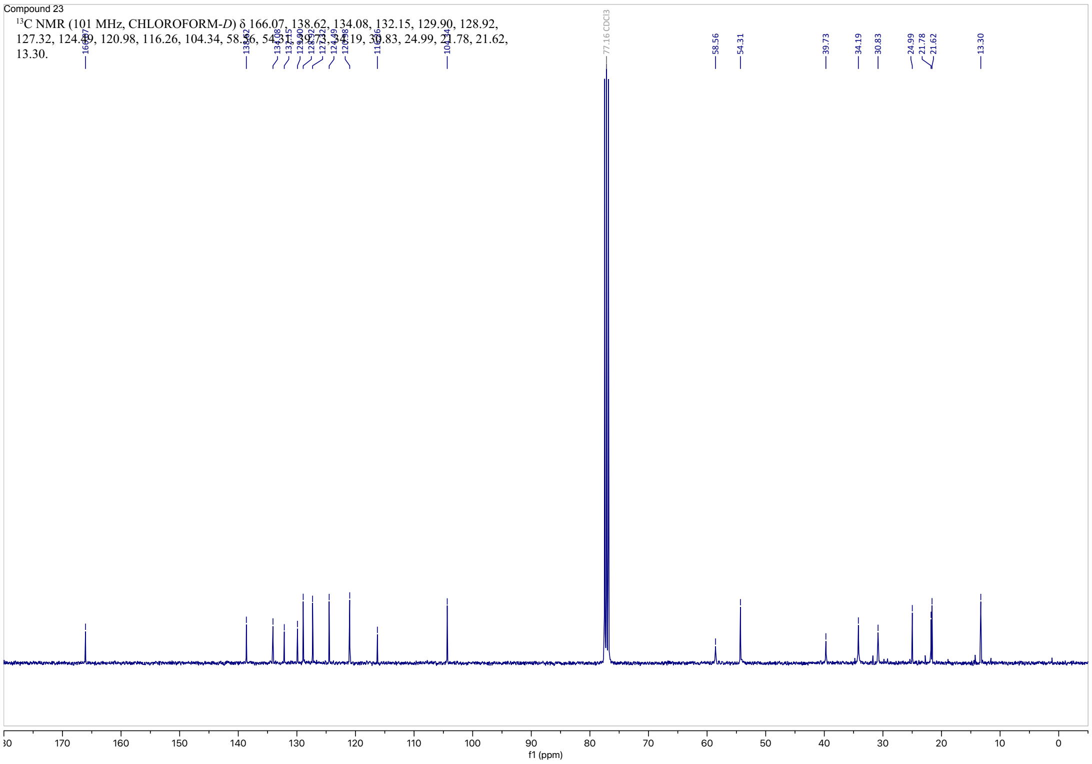
Compound 23
13C NMR (101 MHz, CHLOROFORM-D) δ 166.07, 138.62, 134.08, 132.15, 129.90, 128.92, 127.32, 124.49, 120.98, 116.26, 104.34, 58.56, 54.31, 39.73, 34.19, 30.83, 24.99, 21.78, 21.62, 13.30.
77.16 CDCl3
f1 (ppm)

Compound 24

Compound 24

^{1}H NMR (400 MHz, CHLOROFORM-*D*) δ 8.81 (s, 1H), 7.56 (s, 1H), 7.40 – 7.35 (m, 1H), 7.12 (d, *J* = 7.7 Hz, 1H), 6.60 – 6.55 (m, 1H), 3.47 (s, 2H), 3.03 (d, *J* = 11.2 Hz, 2H), 2.60 (s, 3H), 2.53 (s, 2H), 2.32 (s, 3H), 2.04 – 1.93 (m, 3H), 1.77 (s, 2H), 1.66 (d, *J* = 10.1 Hz, 2H), 1.39 (s, 3H), 0.92 (d, *J* = 5.0 Hz, 3H).

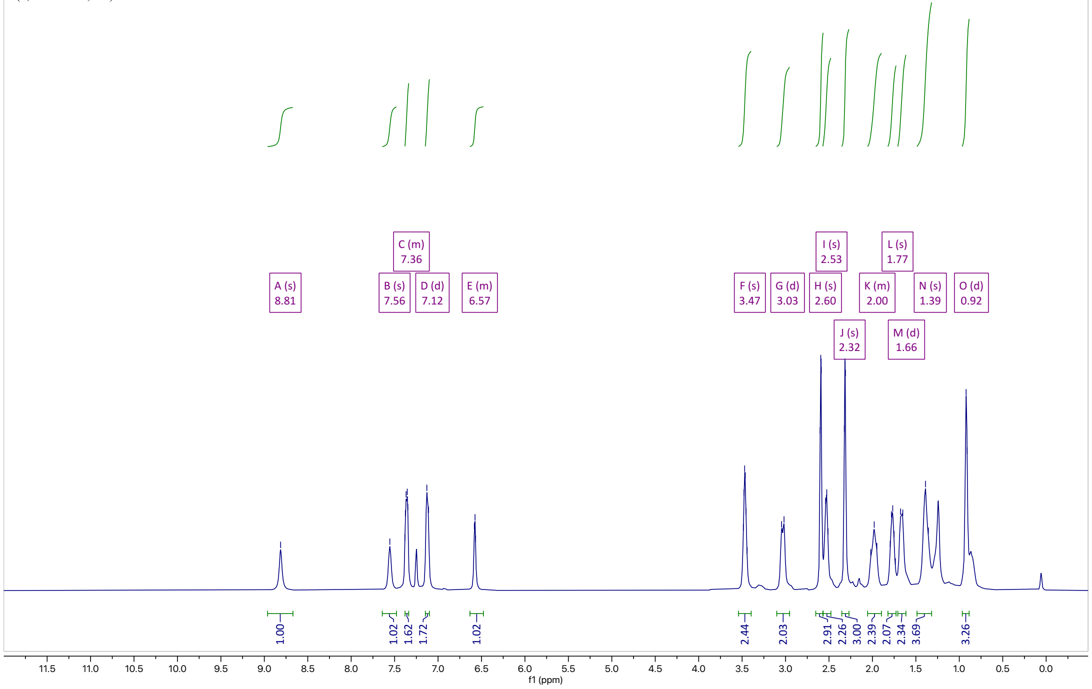

Compound 24

^{13}C NMR (101 MHz, CHLOROFORM-*D*) δ 166.14, 136.19, 133.88, 129.96, 129.65, 129.48, 123.77, 116.10, 103.84, 58.50, 54.31, 39.66, 34.13, 30.82, 24.99, 21.74, 21.23, 13.27.

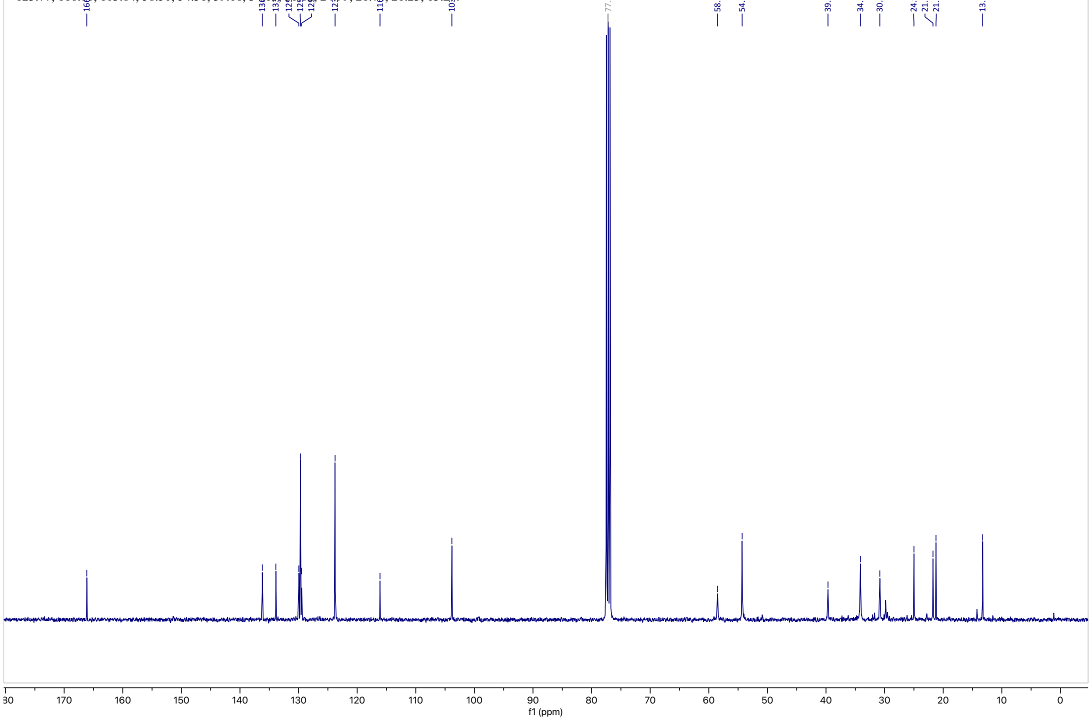

Compound 25

Compound 25

^{1}H NMR (400 MHz, CHLOROFORM-*D*) δ 8.59 (s, 1H), 7.59 (s, 1H), 7.09 (s, 2H), 6.85 (s, 1H), 6.57 (d, *J* = 2.7 Hz, 1H), 3.50 (dq, *J* = 6.9, 3.7 Hz, 2H), 3.03 (d, *J* = 11.2 Hz, 2H), 2.62 (s, 3H), 2.56 – 2.46 (m, 2H), 2.31 (d, *J* = 2.5 Hz, 6H), 2.01 – 1.90 (m, 2H), 1.77 (p, *J* = 6.1 Hz, 2H), 1.68 (d, *J* = 10.8 Hz, 2H), 1.39 (d, *J* = 8.5 Hz, 2H), 1.26 (s, 1H), 0.95 – 0.89 (m, 3H).

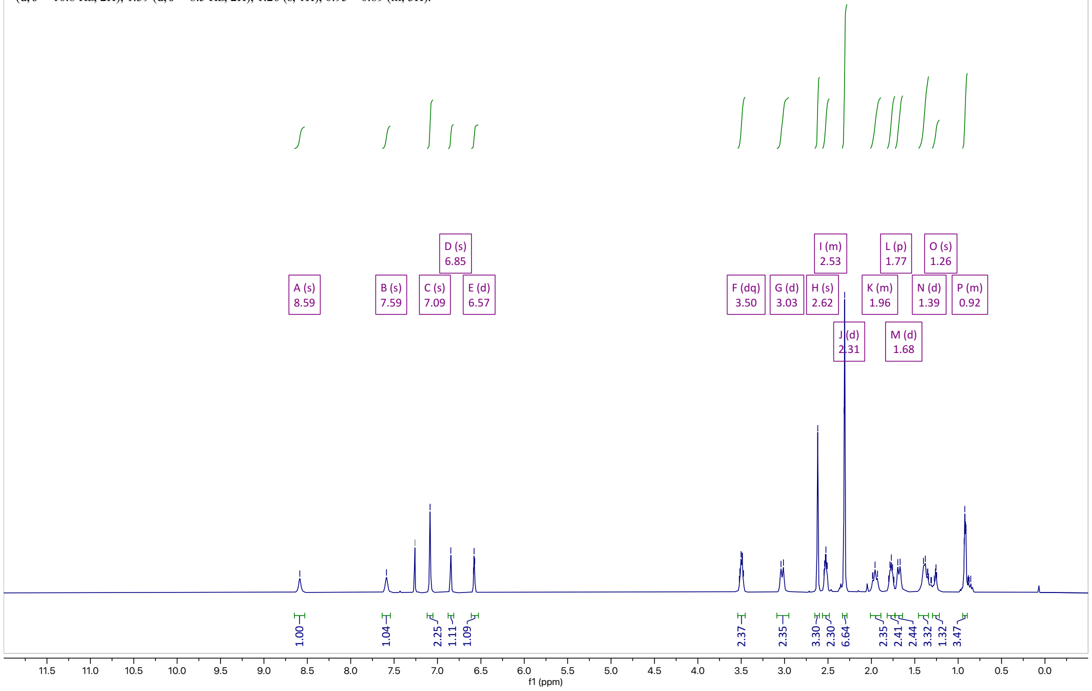

Compound 25

^{13}C NMR (101 MHz, CHLOROFORM-*D*) δ 166.02, 138.53, 133.93, 132.09, 129.96, 128.31, 121.69, 116.25, 104.17, 58.79, 54.38, 39.91, 34.40, 30.91, 25.06, 21.90, 21.48, 13.31.

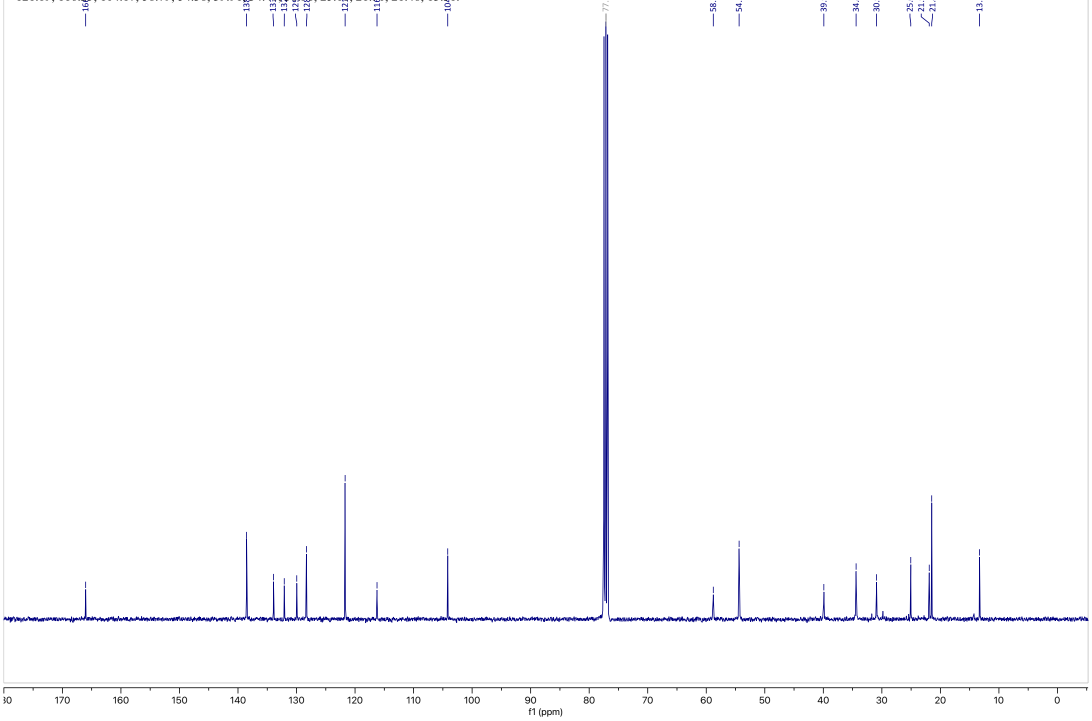

Compound 26

Compound 26

^{1}H NMR (400 MHz, METHANOL-D_4) δ 8.30 (d, J = 5.5 Hz, 1H), 7.45 (s, 1H), 7.37 (d, J = 5.5 Hz, 1H), 7.10 – 7.05 (m, 1H), 3.47 – 3.35 (m, 4H), 2.97 (t, J = 7.4 Hz, 2H), 2.74 (t, J = 12.3 Hz, 2H), 2.57 (d, J = 1.5 Hz, 3H), 2.53 (s, 3H), 1.98 (t, J = 7.1 Hz, 2H), 1.90 (d, J = 17.8 Hz, 2H), 1.67 (s, 1H), 1.45 (q, J = 12.8 Hz, 2H), 1.05 – 0.98 (m, 3H).

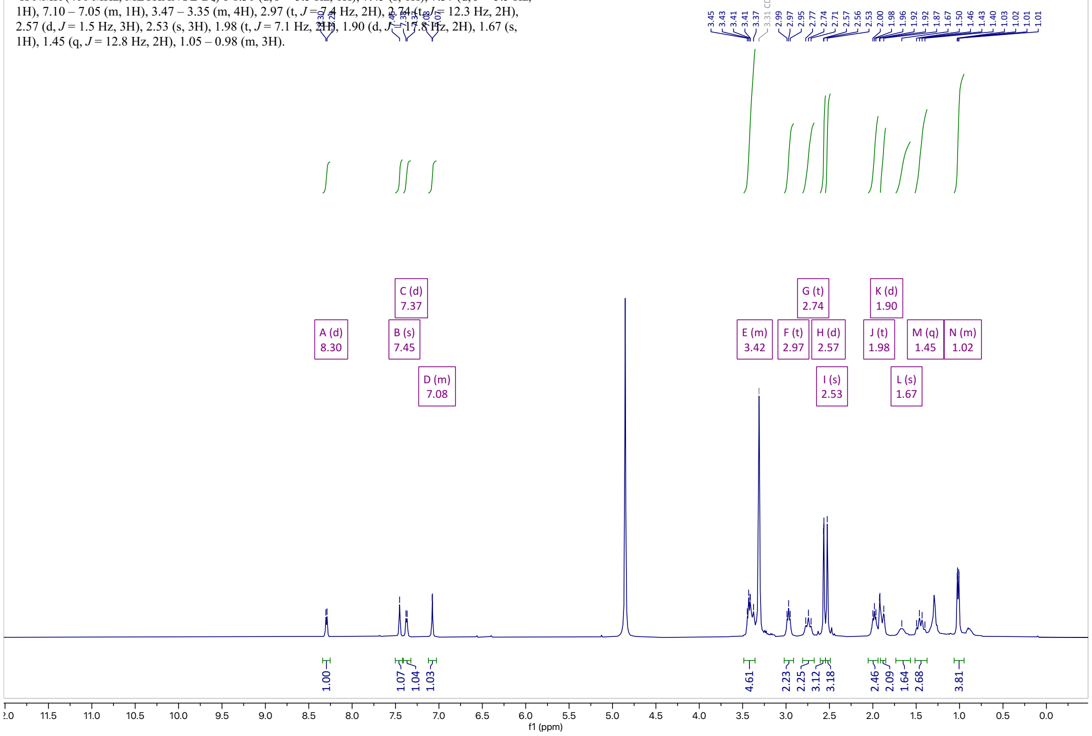

Compound 26

^{13}C NMR (101 MHz, METHANOL-D_4) δ 159.67, 149.85, 142.09, 138.55, 128.43, 118.53, 116.61, 108.93, 55.77, 54.00, 37.24, 33.02, 30.31, 26.44, 23.83, 21.34, 13.00.

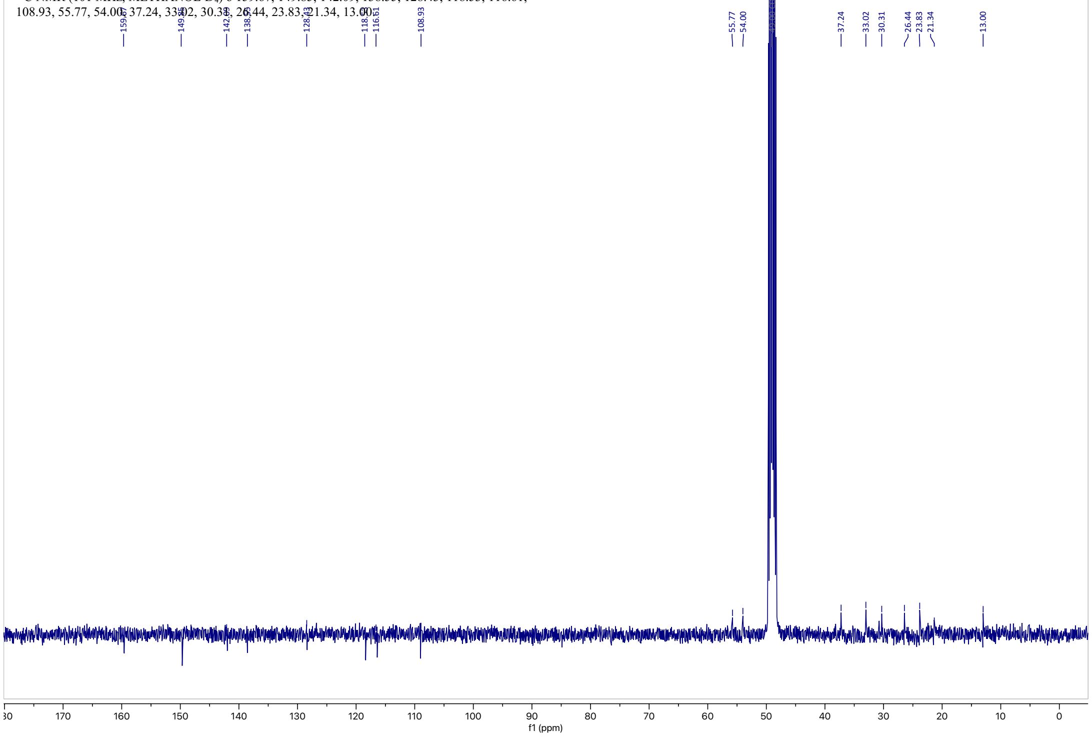

Compound 27

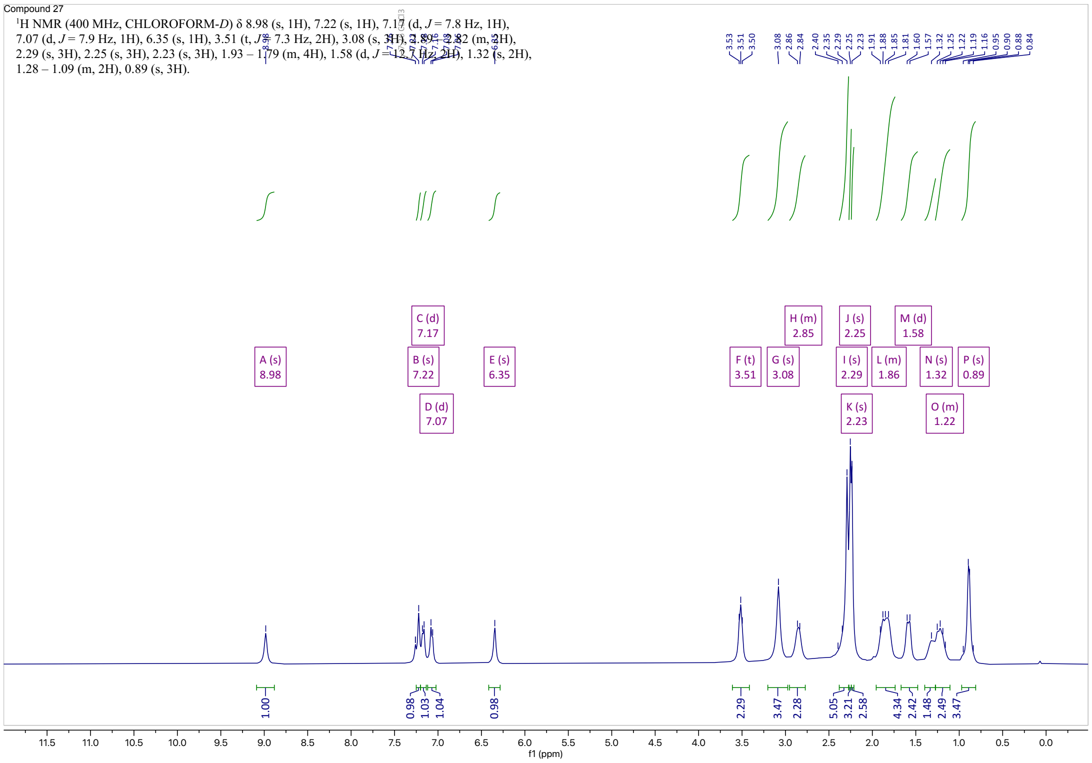
Compound 27
^{1}H NMR (400 MHz, CHLOROFORM-*D*) δ 8.98 (s, 1H), 7.22 (s, 1H), 7.17 (d, *J* = 7.8 Hz, 1H), 7.07 (d, *J* = 7.9 Hz, 1H), 6.35 (s, 1H), 3.51 (t, *J* = 7.3 Hz, 2H), 3.08 (s, 3H), 2.89 – 2.82 (m, 2H), 2.29 (s, 3H), 2.25 (s, 3H), 2.23 (s, 3H), 1.93 – 1.79 (m, 4H), 1.58 (d, *J* = 12.7 Hz, 2H), 1.32 (s, 2H), 1.28 – 1.09 (m, 2H), 0.89 (s, 3H).
f1 (ppm)

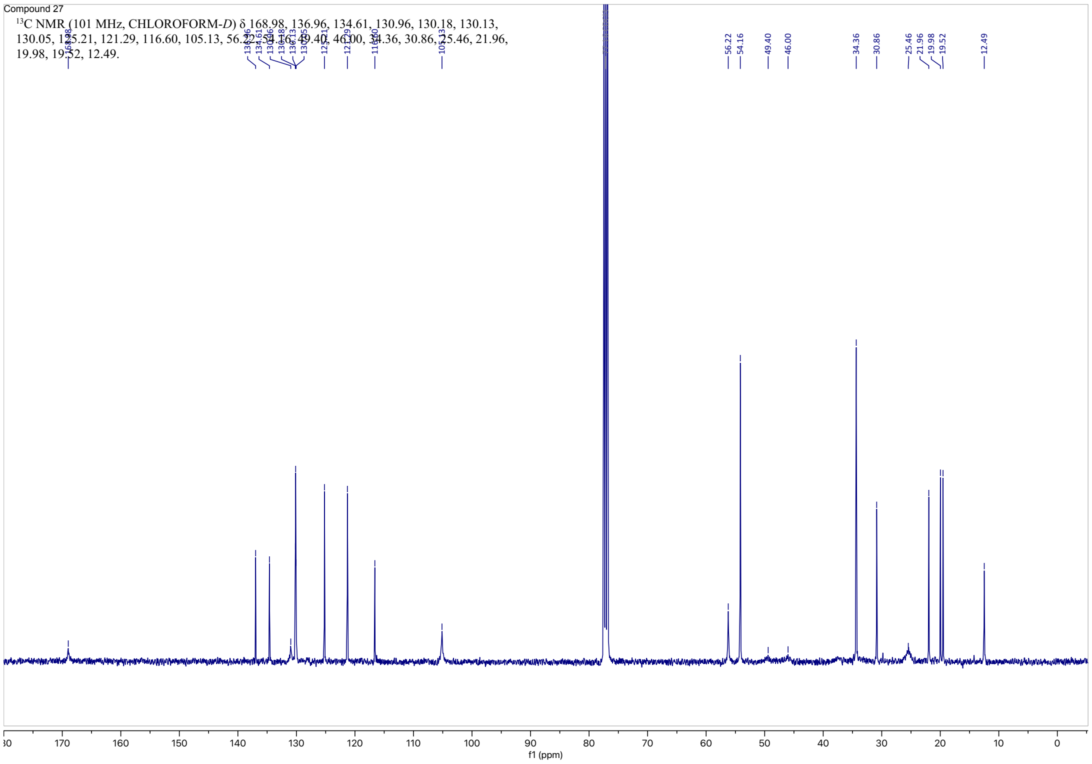
Compound 27
^{13}C NMR (101 MHz, CHLOROFORM-*D*) δ 168.98, 136.96, 134.61, 130.96, 130.18, 130.13, 130.05, 125.21, 121.29, 116.60, 105.13, 56.22, 54.16, 49.40, 46.00, 34.36, 30.86, 25.46, 21.96, 19.98, 19.52, 12.49.
f1 (ppm)

Compound 28

Compound 28

^{1}H NMR (400 MHz, CHLOROFORM-*D*) δ 8.43 (s, 1H), 7.24 (s, 1H), 7.18 (d, *J* = 8.0 Hz, 1H), 7.12 (d, *J* = 7.8 Hz, 1H), 6.76 (d, *J* = 2.9 Hz, 1H), 4.26 (t, *J* = 6.4 Hz, 2H), 2.91 (d, *J* = 11.0 Hz, 2H), 2.57 (s, 3H), 2.47 (t, *J* = 7.7 Hz, 2H), 2.28 (s, 3H), 2.26 (s, 3H), 2.00 – 1.88 (m, 4H), 1.62 (d, 2H), 1.35 (s, 1H), 1.26 (dt, *J* = 12.3, 7.8 Hz, 2H), 0.92 (d, *J* = 6.3 Hz, 3H).

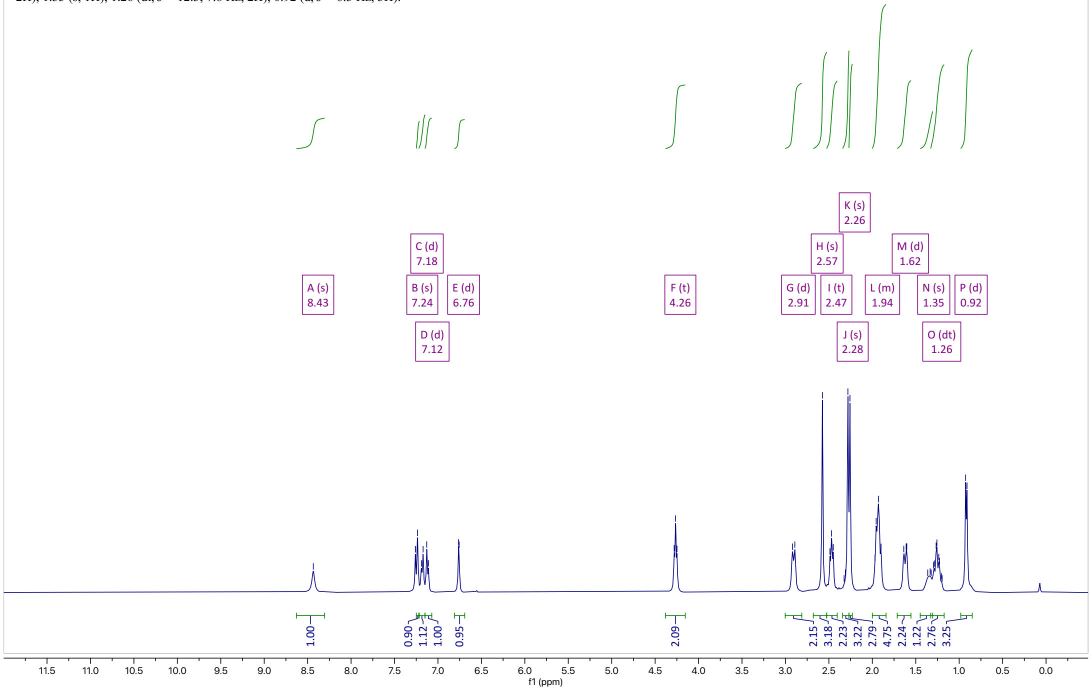

Compound 28

^{13}C NMR (101 MHz, CHLOROFORM-*D*) δ 165.73, 137.26, 135.78, 135.28, 130.35, 130.29, 129.61, 125.25, 121.29, 113.32, 106.85, 62.39, 55.97, 54.22, 34.49, 30.96, 26.84, 22.04, 20.01, 19.58, 13.58.

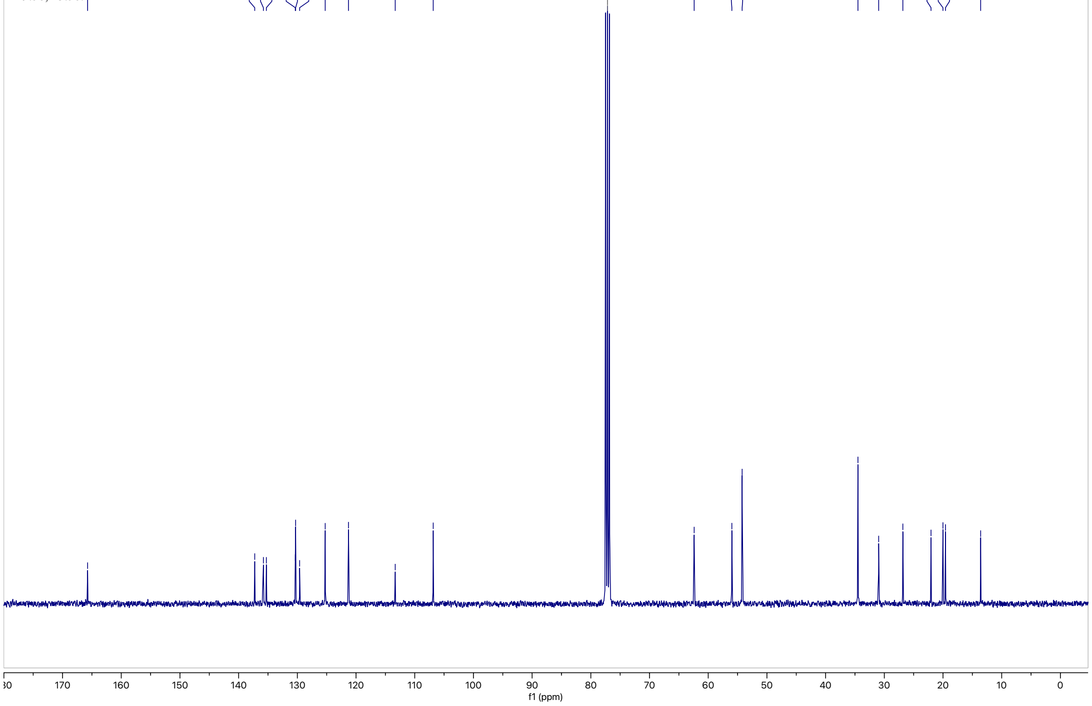

Compound 29

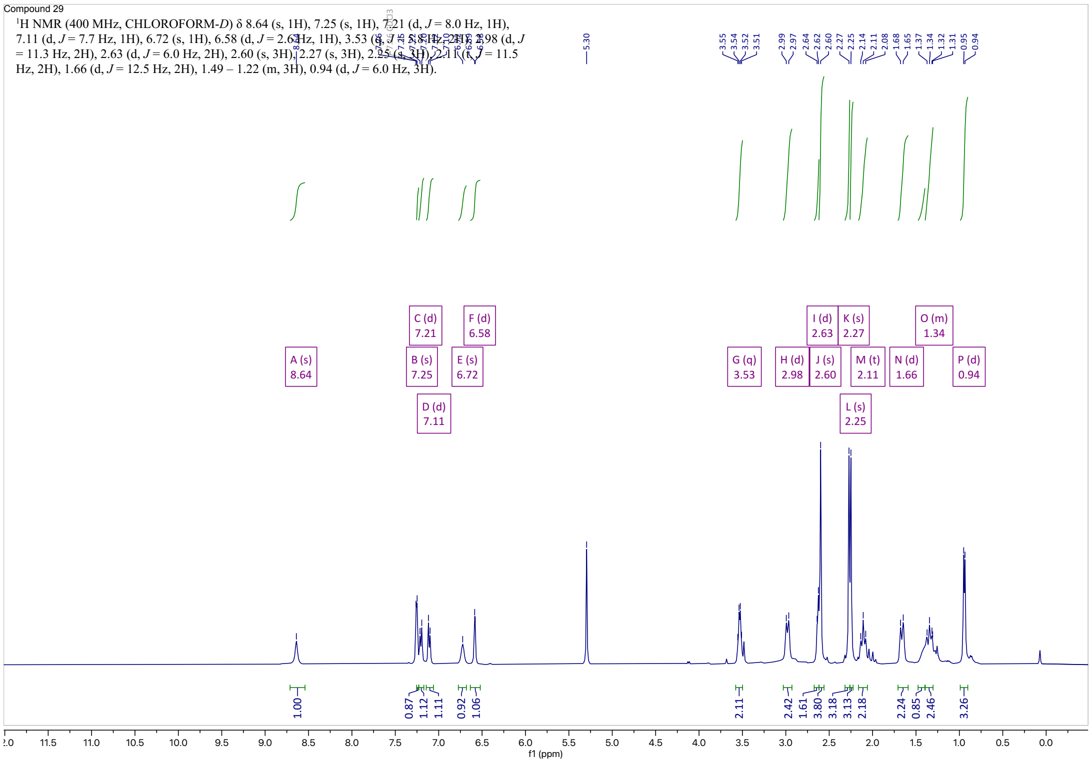
Compound 29
1H NMR (400 MHz, CHLOROFORM-D) δ 8.64 (s, 1H), 7.25 (s, 1H), 7.21 (d, J = 8.0 Hz, 1H), 7.11 (d, J = 7.7 Hz, 1H), 6.72 (s, 1H), 6.58 (d, J = 2.6 Hz, 1H), 3.53 (q, J = 5.8 Hz, 2H), 2.98 (d, J = 11.3 Hz, 2H), 2.63 (d, J = 6.0 Hz, 2H), 2.60 (s, 3H), 2.27 (s, 3H), 2.25 (s, 3H), 2.11 (t, J = 11.5 Hz, 2H), 1.66 (d, J = 12.5 Hz, 2H), 1.49 – 1.22 (m, 3H), 0.94 (d, J = 6.0 Hz, 3H).
f1 (ppm)

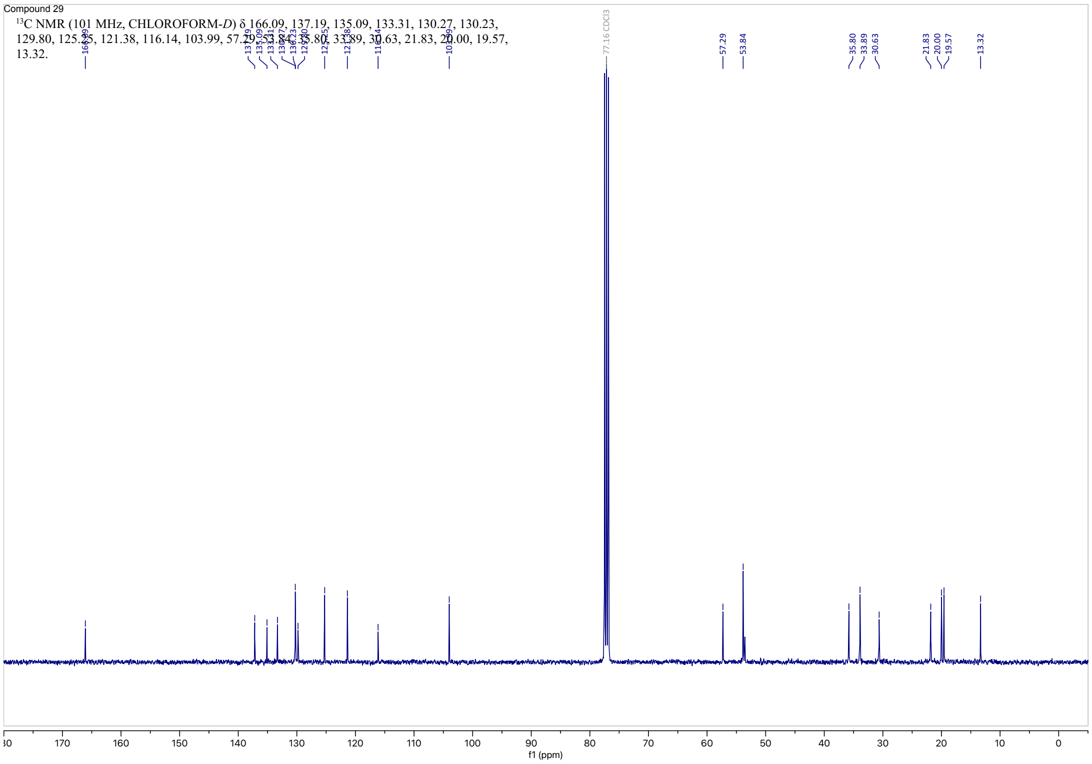
Compound 29
13C NMR (101 MHz, CHLOROFORM-D) δ 166.09, 137.19, 135.09, 133.31, 130.27, 130.23, 129.80, 125.25, 121.38, 116.14, 103.99, 57.29, 53.84, 35.80, 33.89, 30.63, 21.83, 20.00, 19.57, 13.32.
77.16 CDCl3
f1 (ppm)

Compound 30

Compound 30

^{1}H NMR (400 MHz, CHLOROFORM-*D*) δ 8.91 (s, 1H), 7.24 (s, 1H), 7.19 (d, *J* = 7.6 Hz, 1H), 7.08 (d, *J* = 7.9 Hz, 1H), 6.45 (s, 1H), 6.06 (s, 1H), 3.41 – 3.35 (m, 2H), 2.91 – 2.83 (m, 2H), 2.56 (s, 3H), 2.32 (s, 3H), 2.24 (d, *J* = 6.7 Hz, 6H), 1.93 – 1.83 (m, 2H), 1.57 (s, 7H), 1.26 (d, *J* = 14.7 Hz, 4H).

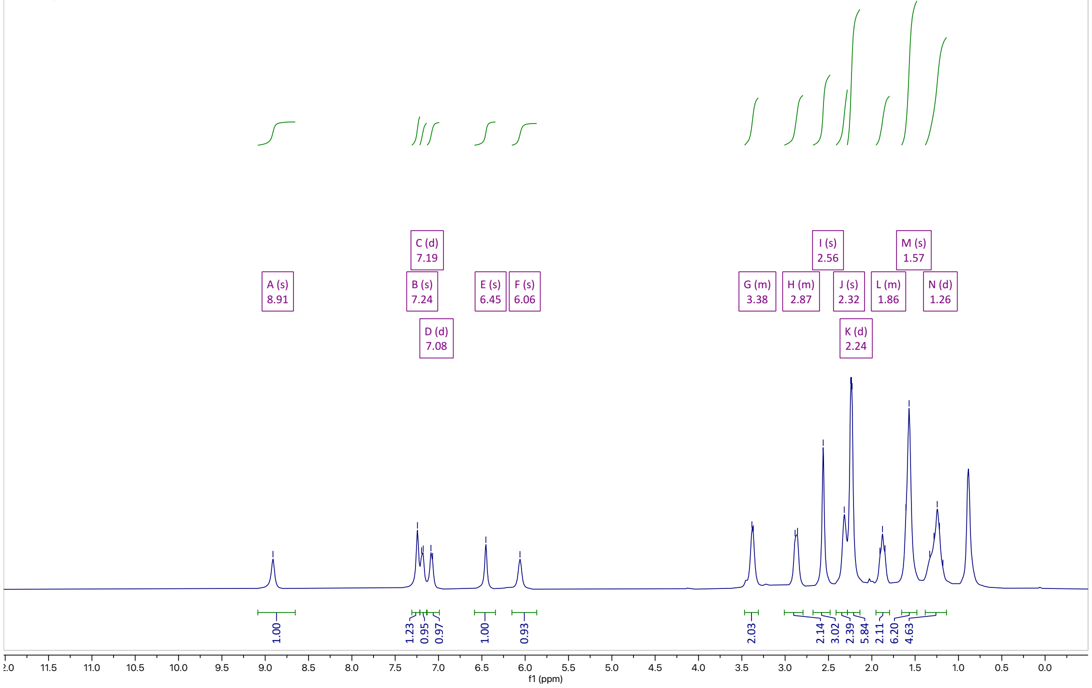

Compound 30

^{13}C NMR (101 MHz, CHLOROFORM-*D*) δ 166.11, 137.11, 134.97, 133.63, 130.24, 130.21, 129.79, 125.21, 121.26, 115.96, 103.37, 58.62, 54.10, 39.25, 34.27, 30.87, 28.02, 24.64, 21.95, 19.95, 19.54, 13.18.

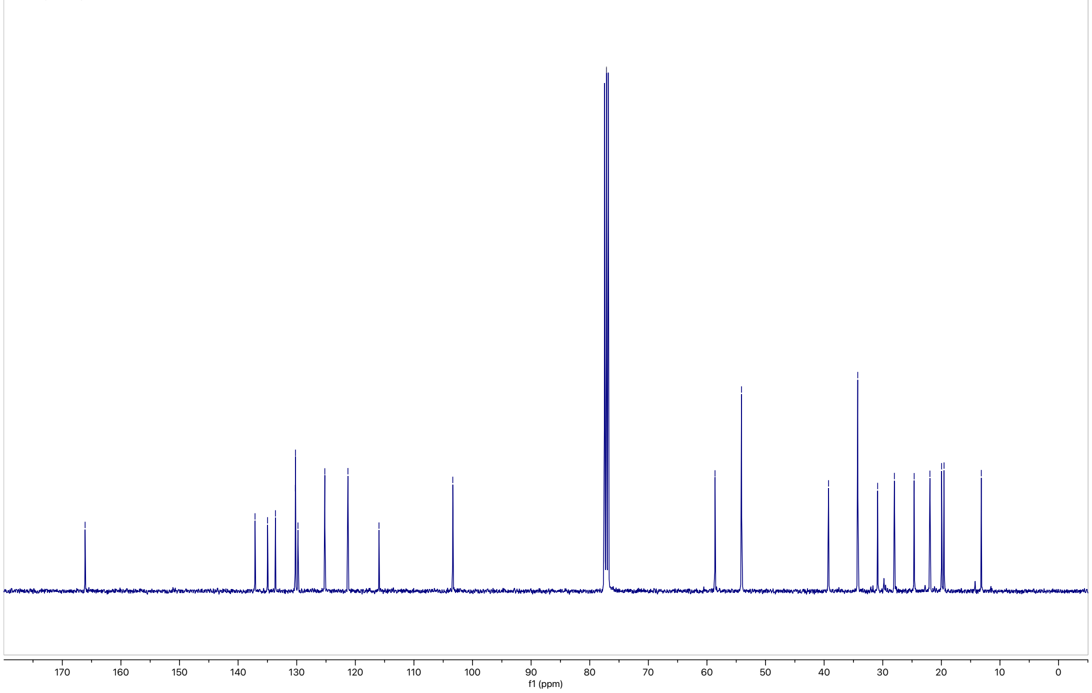

Compound 31

Compound 31

^{1}H NMR (400 MHz, CHLOROFORM-*D*) δ 8.70 (s, 1H), 7.24 (s, 1H), 7.19 (d, *J* = 7.8 Hz, 1H), 7.10 (d, *J* = 7.9 Hz, 1H), 6.45 (d, *J* = 2.8 Hz, 1H), 5.83 (t, *J* = 5.9 Hz, 1H), 3.38 (q, *J* = 6.9 Hz, 2H), 2.88 (d, *J* = 11.1 Hz, 2H), 2.59 (s, 3H), 2.27 (s, 3H), 2.25 (s, 3H), 1.89 (t, *J* = 11.6 Hz, 2H), 1.65 – 1.47 (m, 7H), 1.35 (p, *J* = 7.8 Hz, 3H), 0.90 (d, *J* = 6.2 Hz, 3H).

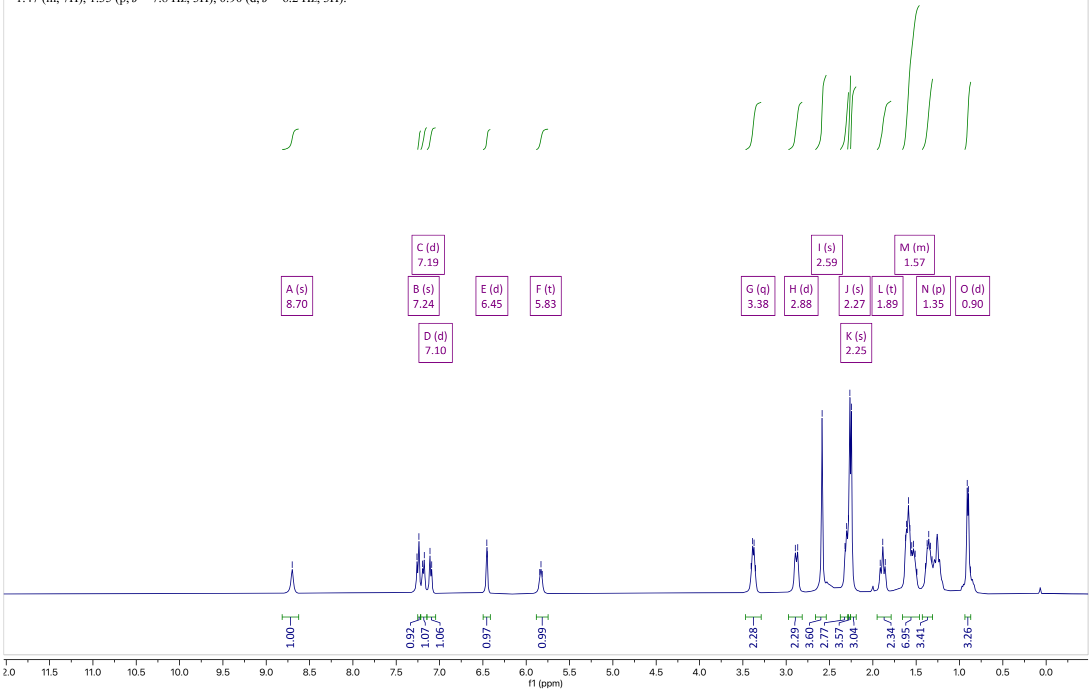

Compound 31

^{13}C NMR (101 MHz, CHLOROFORM-*D*) δ 165.97, 137.19, 135.12, 133.63, 130.27, 129.73, 125.21, 121.28, 115.99, 103.28, 59.07, 54.12, 39.32, 34.31, 30.92, 30.08, 26.81, 25.28, 21.99, 19.99, 19.56, 13.23.

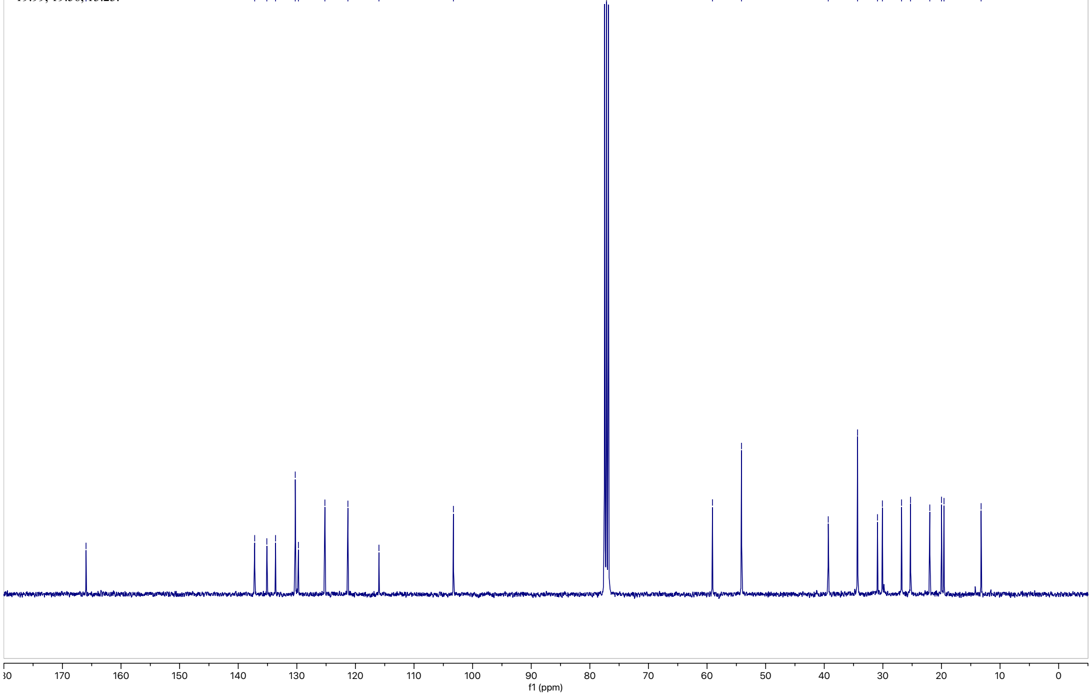

Compound 34

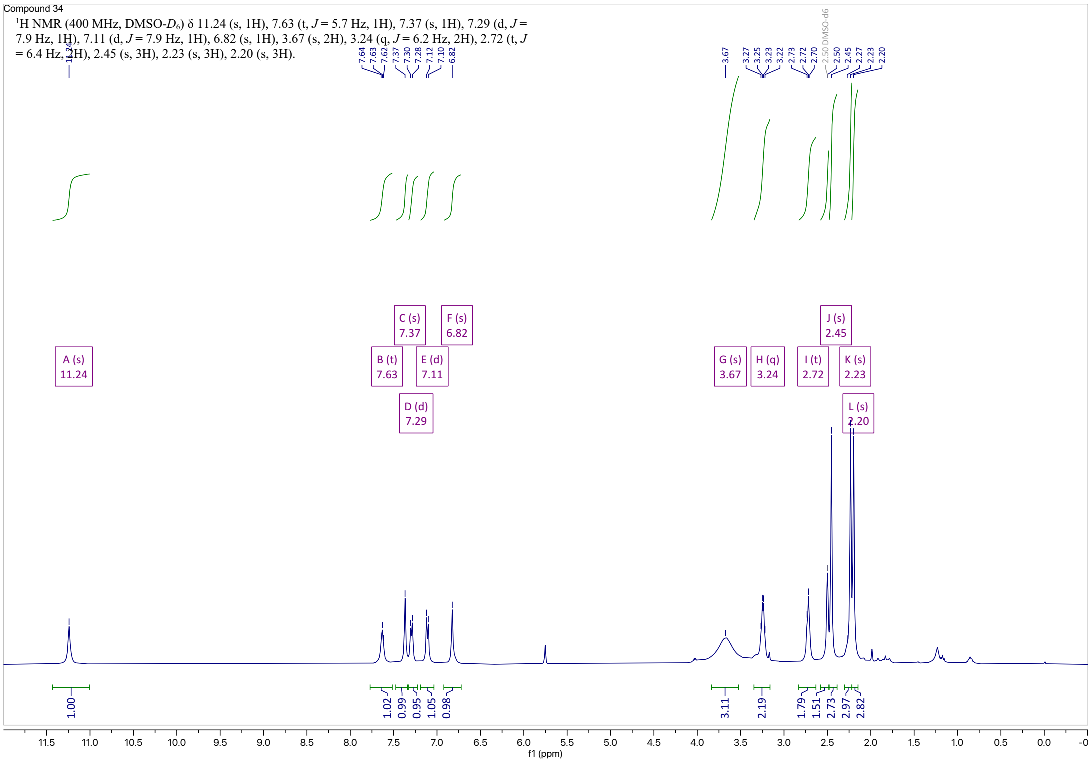
Compound 34
^{1}H NMR (400 MHz, DMSO-D_6) δ 11.24 (s, 1H), 7.63 (t, J = 5.7 Hz, 1H), 7.37 (s, 1H), 7.29 (d, J = 7.9 Hz, 1H), 7.11 (d, J = 7.9 Hz, 1H), 6.82 (s, 1H), 3.67 (s, 2H), 3.24 (q, J = 6.2 Hz, 2H), 2.72 (t, J = 6.4 Hz, 2H), 2.45 (s, 3H), 2.23 (s, 3H), 2.20 (s, 3H).
f1 (ppm)

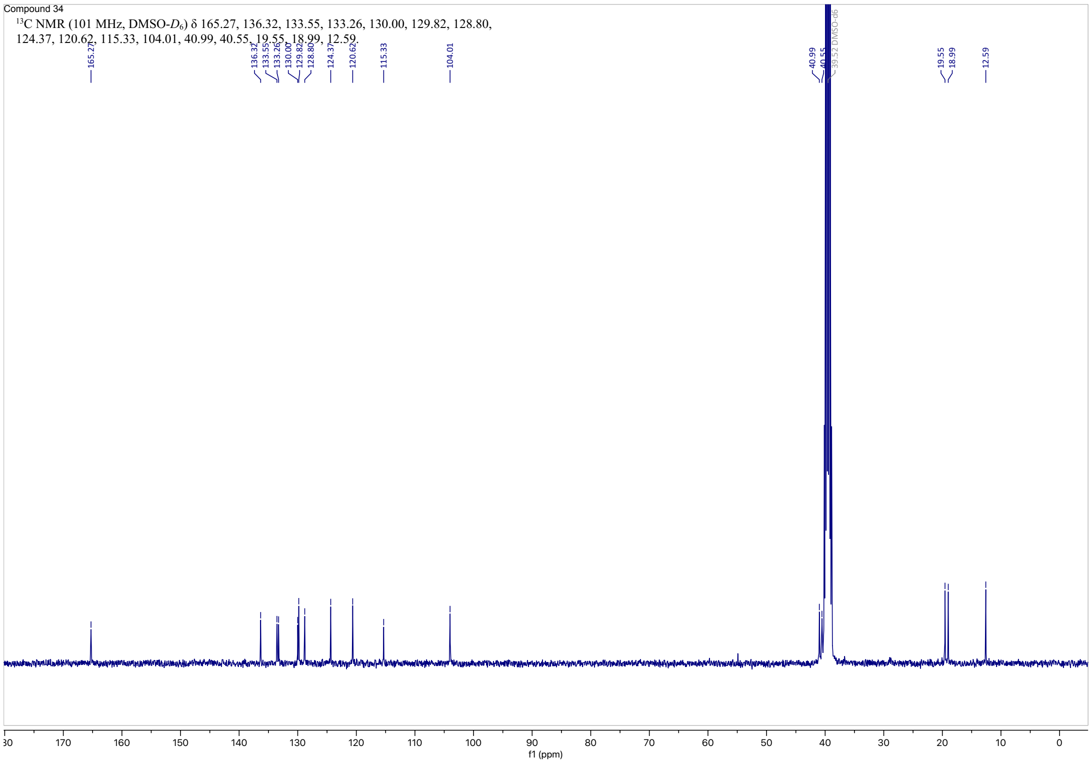
Compound 34
^{13}C NMR (101 MHz, DMSO-D_6) δ 165.27, 136.32, 133.55, 133.26, 130.00, 129.82, 128.80, 124.37, 120.62, 115.33, 104.01, 40.99, 40.55, 19.55, 18.99, 12.59.
f1 (ppm)

Compound 35

Compound 35

^{1}H NMR (400 MHz, CHLOROFORM-*D*) δ 8.73 (s, 1H), 7.23 (s, 1H), 7.19 (d, *J* = 7.8 Hz, 1H), 7.08 (d, *J* = 8.0 Hz, 1H), 6.77 (s, 1H), 6.60 (s, 1H), 4.15 (s, 1H), 3.55 (s, 2H), 2.90 (s, 2H), 2.67 (t, *J* = 7.3 Hz, 2H), 2.58 (s, 3H), 2.25 (d, *J* = 6.9 Hz, 6H), 1.59 (q, *J* = 8.2 Hz, 2H), 0.97 – 0.83 (m, 3H).

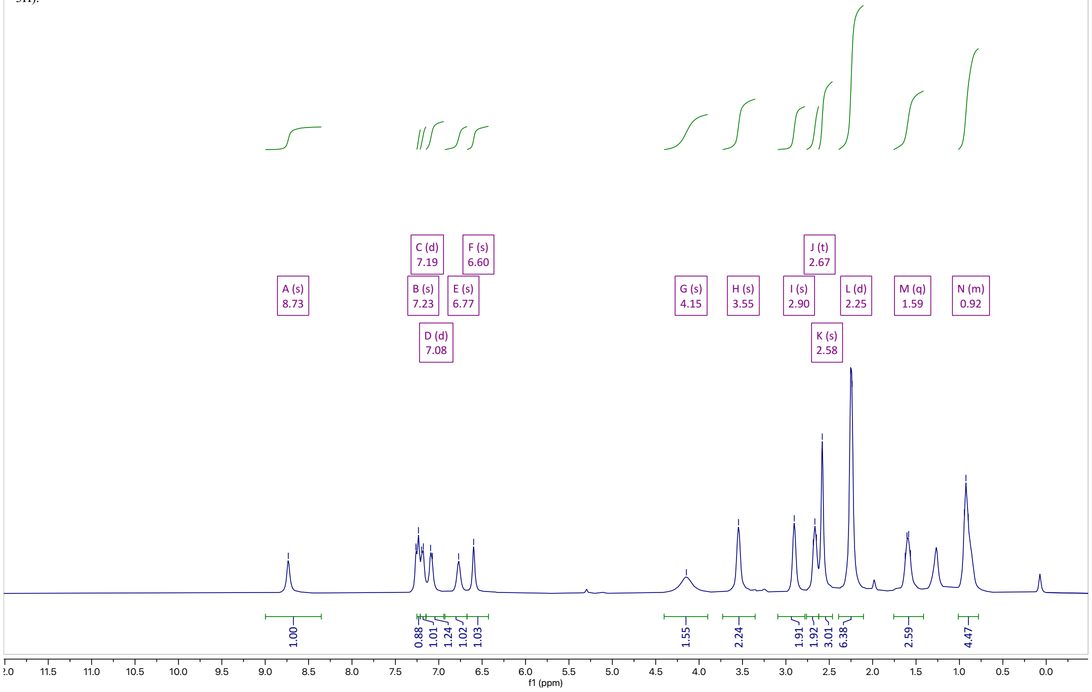

Compound 35

^{13}C NMR (101 MHz, CHLOROFORM-*D*) δ 166.50, 137.17, 135.09, 133.84, 130.27, 129.72, 125.17, 121.33, 115.71, 103.85, 51.10, 48.97, 38.32, 22.29, 19.99, 19.56, 13.31, 11.69.

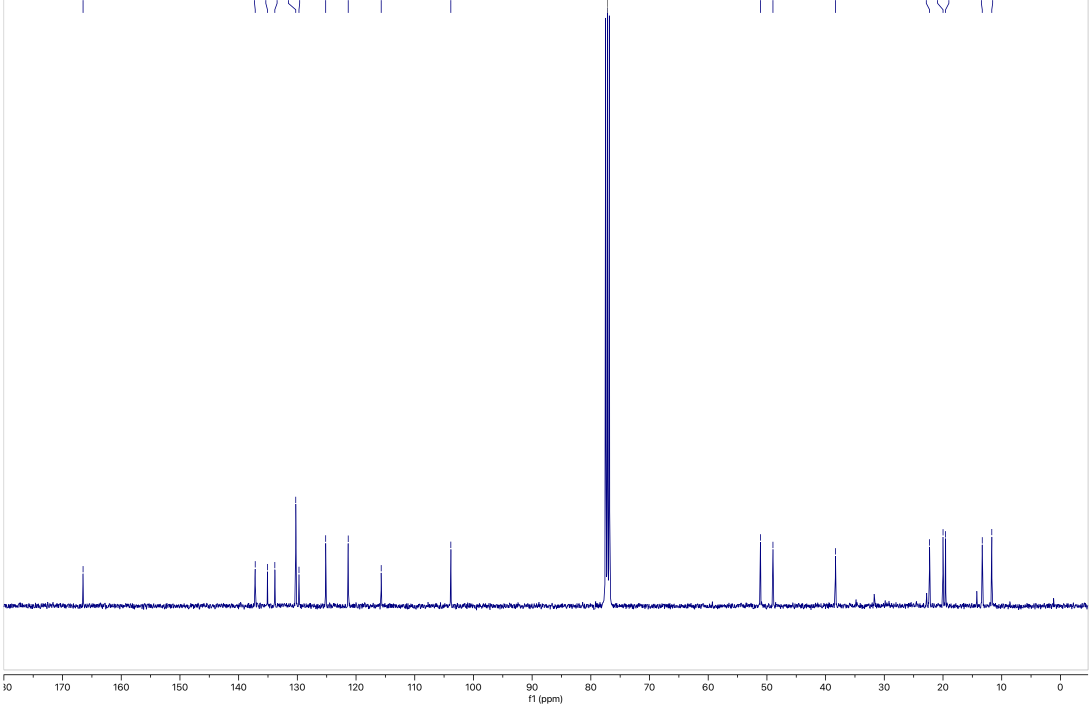

Compound 36

Compound 36

^{1}H NMR (400 MHz, CHLOROFORM-*D*) δ 8.29 (s, 1H), 7.20 (s, 1H), 7.15 (d, 1H), 7.11 (d, *J* = 7.8 Hz, 1H), 6.38 (s, 1H), 3.62 (t, *J* = 6.5 Hz, 2H), 3.50 – 3.41 (m, 2H), 2.92 (s, 2H), 2.64 (s, 2H), 2.39 (s, 3H), 2.28 (s, 3H), 2.26 (s, 3H), 1.72 – 1.43 (m, 4H), 0.97 – 0.86 (m, 6H).

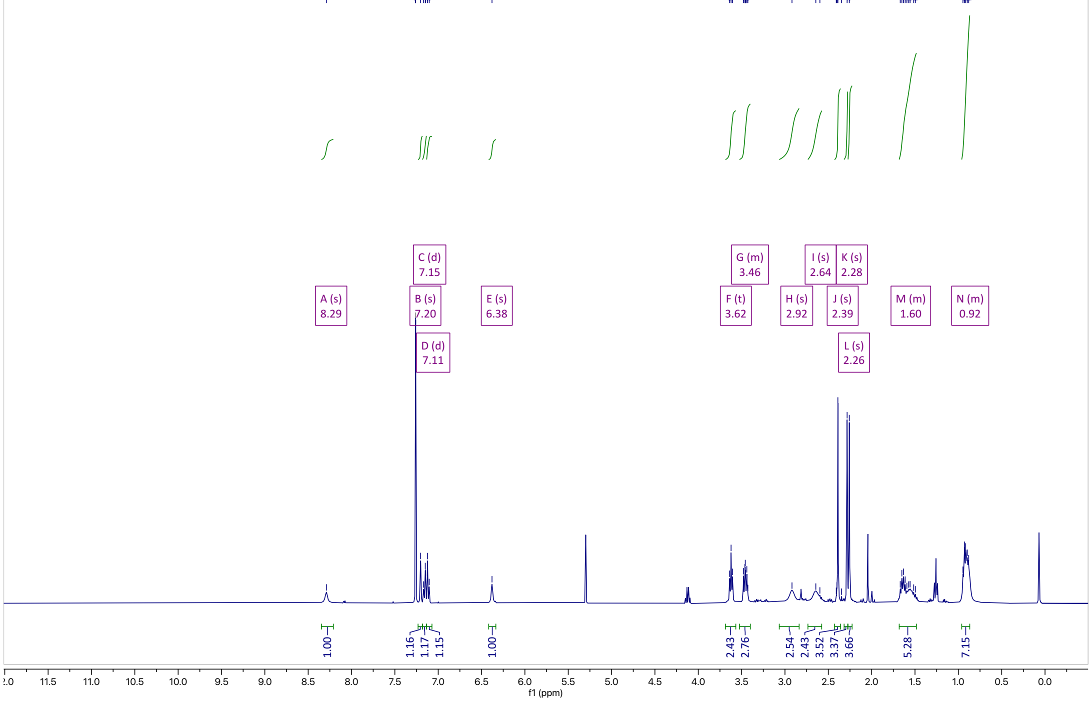

Compound 36

^{13}C NMR (101 MHz, CHLOROFORM-*D*) δ 166.84, 137.24, 135.06, 130.30, 130.19, 125.17, 122.42, 121.27, 104.91, 60.55, 48.24, 20.04, 19.58, 14.35, 12.64, 11.78, 11.31.

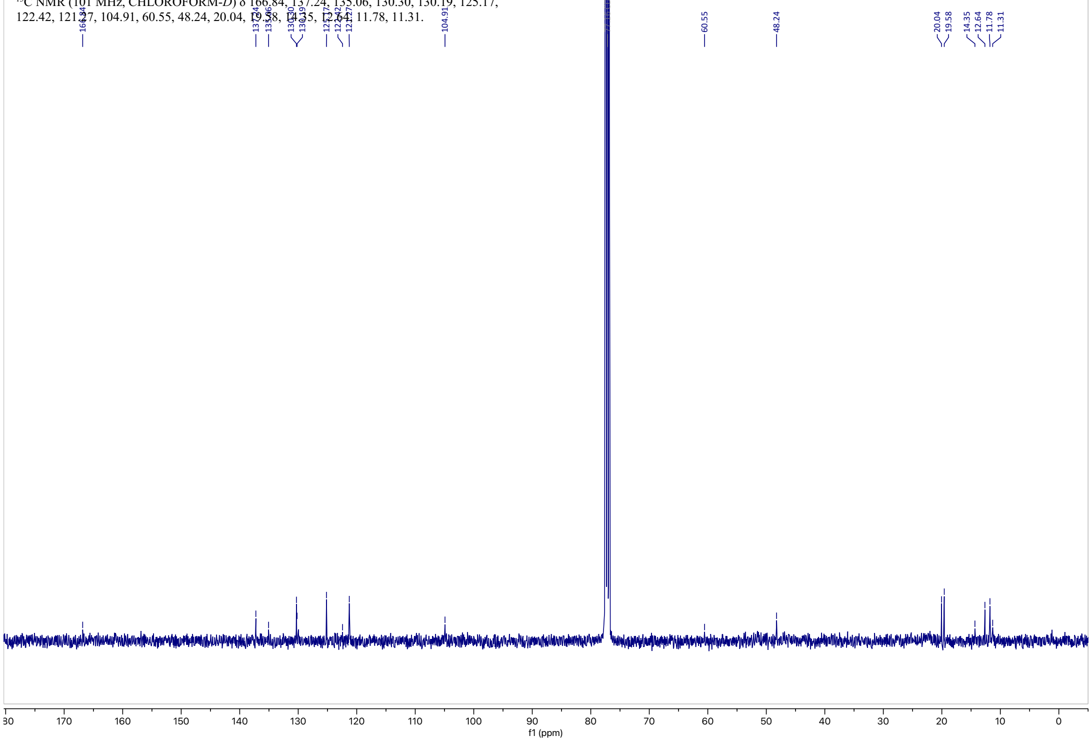

Compound 37

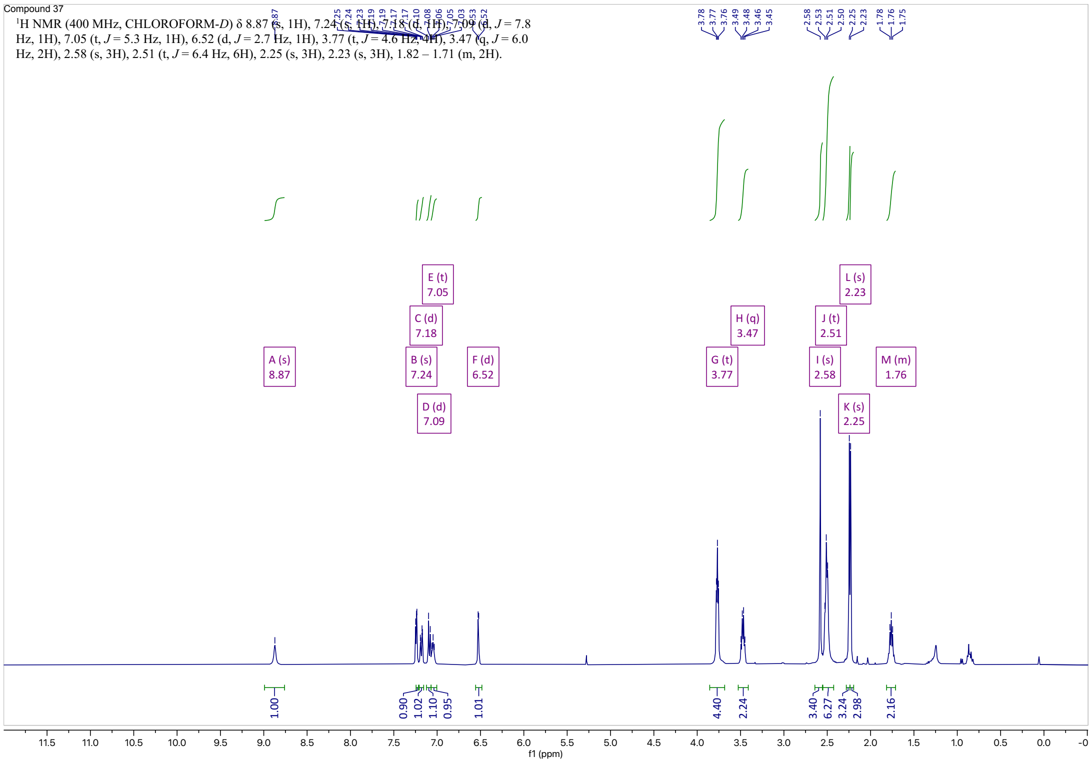

Compound 37
¹H NMR (400 MHz, CHLOROFORM-*D*) δ 8.87 (s, 1H), 7.24 (s, 1H), 7.18 (d, 1H), 7.09 (d, *J* = 7.8 Hz, 1H), 7.05 (t, *J* = 5.3 Hz, 1H), 6.52 (d, *J* = 2.7 Hz, 1H), 3.77 (t, *J* = 4.6 Hz, 4H), 3.47 (q, *J* = 6.0 Hz, 2H), 2.58 (s, 3H), 2.51 (t, *J* = 6.4 Hz, 6H), 2.25 (s, 3H), 2.23 (s, 3H), 1.82 – 1.71 (m, 2H).
A (s) 8.87
B (s) 7.24
C (d) 7.18
D (d) 7.09
E (t) 7.05
F (d) 6.52
G (t) 3.77
H (q) 3.47
I (s) 2.58
J (t) 2.51
K (s) 2.25
L (s) 2.23
M (m) 1.76
f1 (ppm)

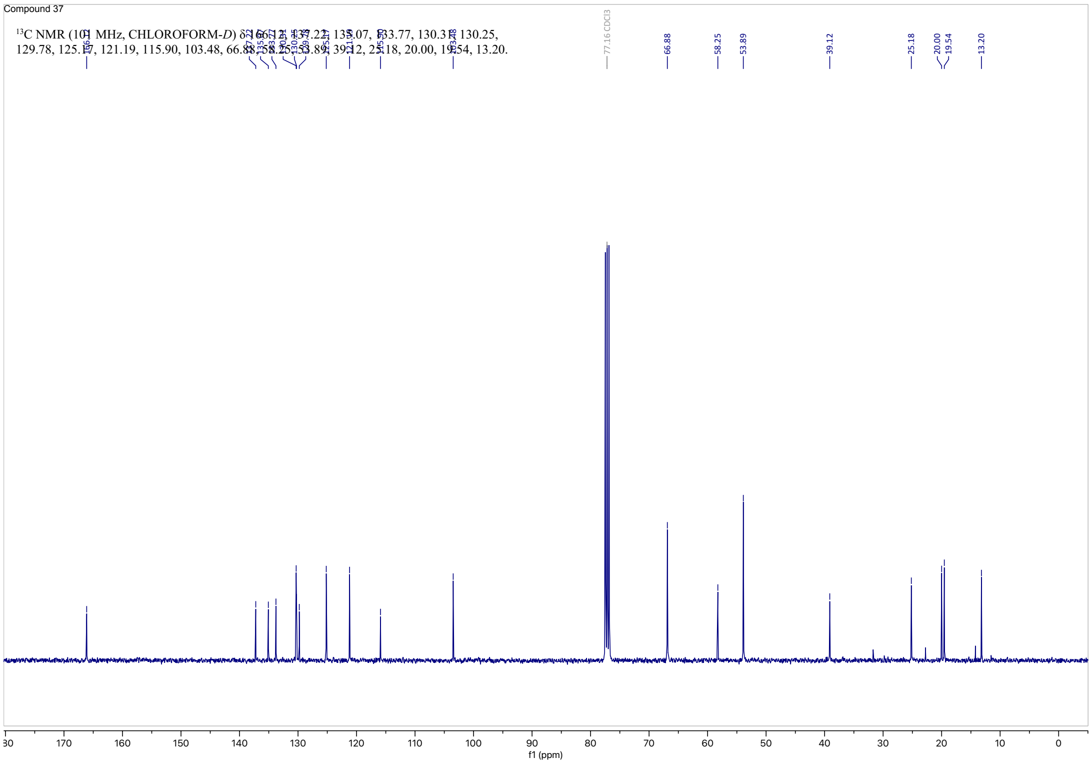

Compound 37
¹³C NMR (101 MHz, CHLOROFORM-*D*) δ 166.12, 137.22, 135.07, 133.77, 130.31, 130.25, 129.78, 125.17, 121.19, 115.90, 103.48, 66.88, 58.25, 53.89, 39.12, 25.18, 20.00, 19.54, 13.20.
77.16 CDCl3
f1 (ppm)

Compound 39

Compound 39

^{1}H NMR (400 MHz, DMSO-D_6) δ 11.20 (s, 1H), 7.60 (t, J = 5.7 Hz, 1H), 7.36 (s, 1H), 7.29 (d, J = 7.9 Hz, 1H), 7.11 (d, J = 7.9 Hz, 1H), 6.76 (s, 1H), 3.25 (t, J = 5.5 Hz, 2H), 3.23 – 3.14 (m, 2H), 2.81 (s, 3H), 2.63 (t, 2H), 2.45 (s, 3H), 2.38 (t, J = 6.9 Hz, 2H), 2.24 (s, 3H), 2.20 (s, 3H), 2.09 (s, 2H), 1.64 (t, 2H).

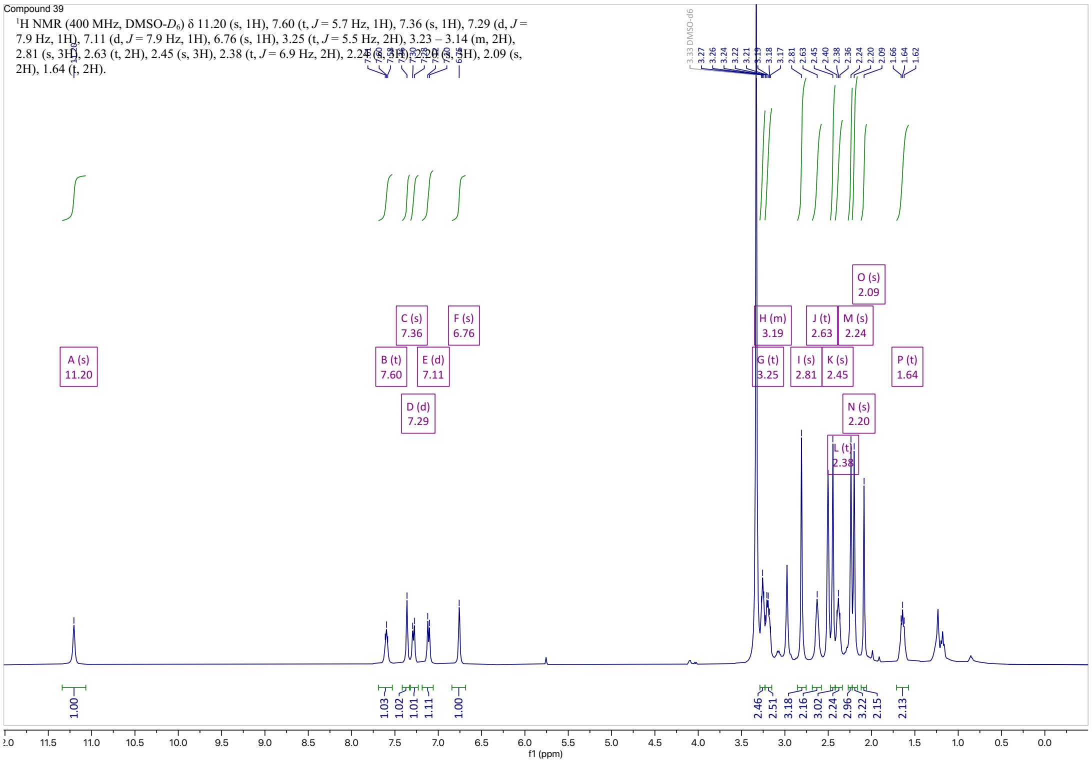

Compound 39

^{13}C NMR (101 MHz, DMSO-D_6) δ 165.96, 164.92, 136.32, 133.53, 133.04, 130.01, 129.81, 128.76, 124.37, 120.62, 115.52, 103.81, 57.04, 54.69, 49.23, 47.92, 36.70, 32.97, 30.68, 26.54, 19.55, 19.00, 12.57.

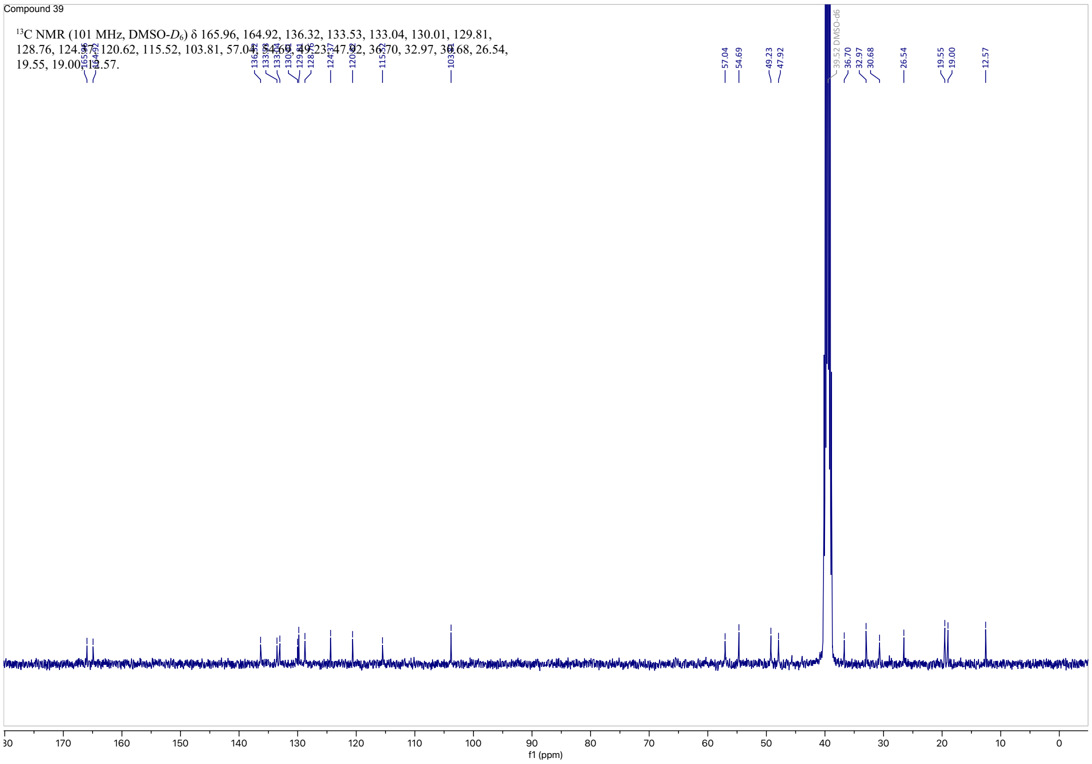

Compound 40

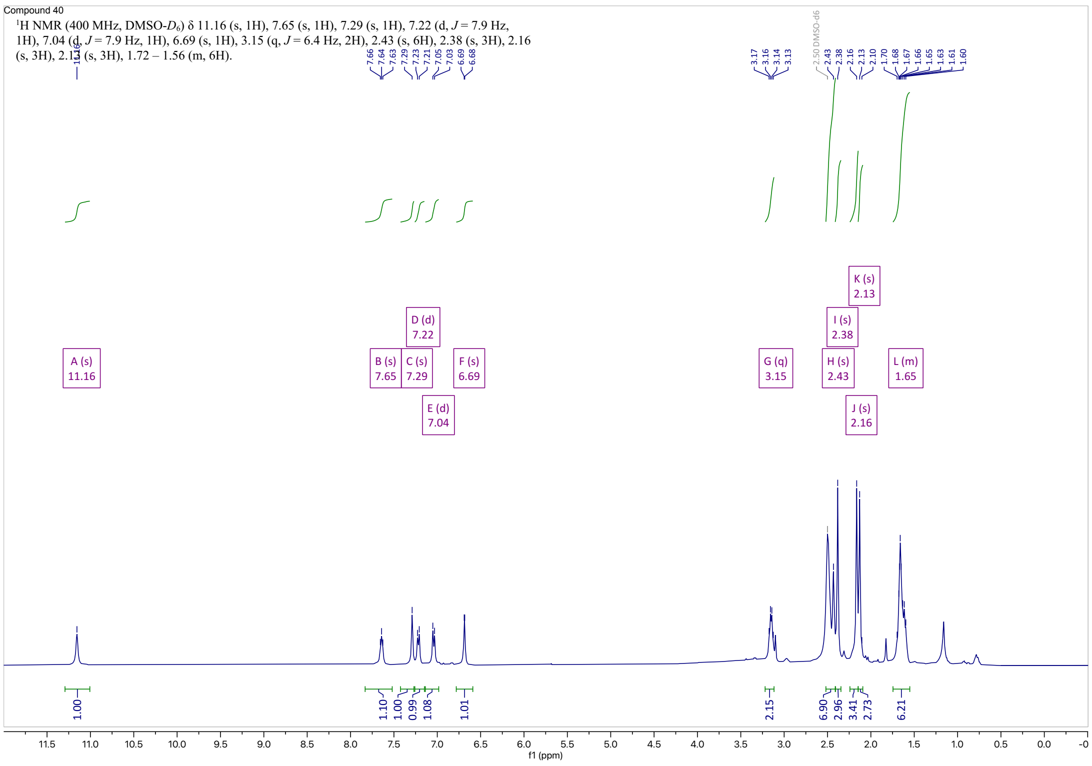
Compound 40
^{1}H NMR (400 MHz, DMSO-D_6) δ 11.16 (s, 1H), 7.65 (s, 1H), 7.29 (s, 1H), 7.22 (d, J = 7.9 Hz, 1H), 7.04 (d, J = 7.9 Hz, 1H), 6.69 (s, 1H), 3.15 (q, J = 6.4 Hz, 2H), 2.43 (s, 6H), 2.38 (s, 3H), 2.16 (s, 3H), 2.13 (s, 3H), 1.72 – 1.56 (m, 6H).
f1 (ppm)

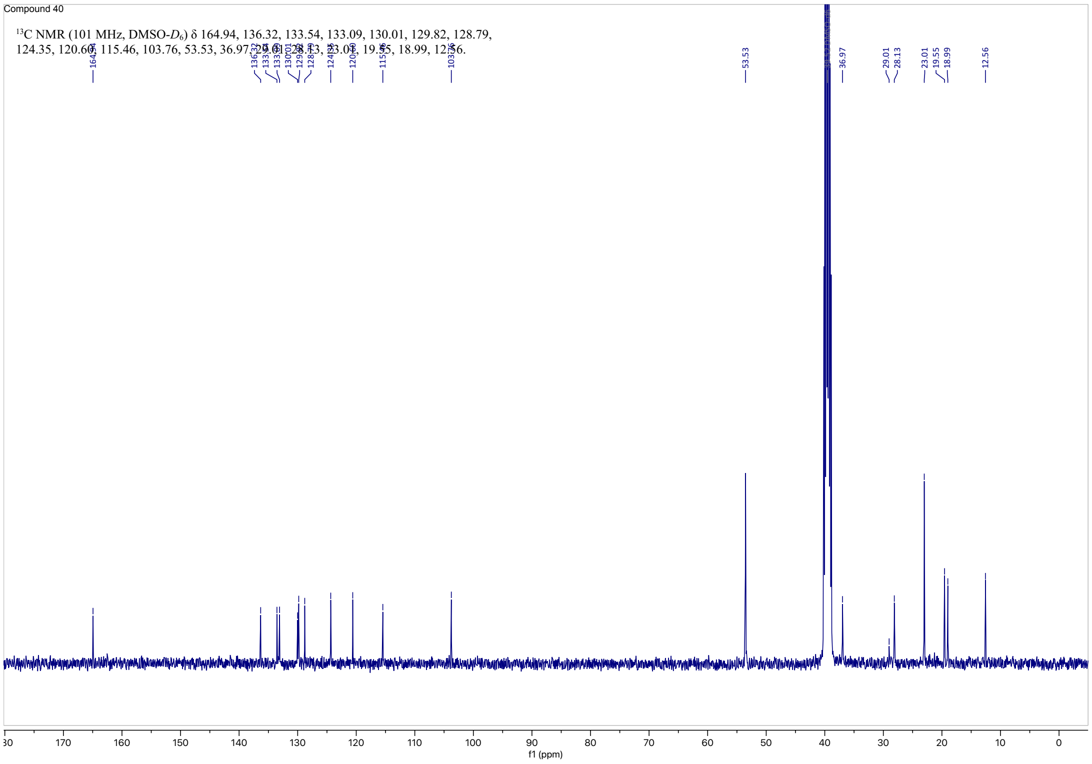
Compound 40
^{13}C NMR (101 MHz, DMSO-D_6) δ 164.94, 136.32, 133.54, 133.09, 130.01, 129.82, 128.79, 124.35, 120.60, 115.46, 103.76, 53.53, 36.97, 29.01, 28.13, 23.01, 19.55, 18.99, 12.56.
f1 (ppm)

Compound 41

Compound 41

^{1}H NMR (400 MHz, DMSO-D_6) δ 11.23 (s, 1H), 7.74 (s, 1H), 7.32 (s, 1H), 7.25 (d, J = 7.9 Hz, 1H), 7.08 (d, J = 7.9 Hz, 1H), 6.75 (d, J = 2.6 Hz, 1H), 3.23 – 3.03 (m, 4H), 2.66 (s, 4H), 2.42 (s, 3H), 2.20 (s, 3H), 2.16 (s, 3H), 1.73 (s, 2H), 1.61 (s, 4H), 1.43 (s, 2H).

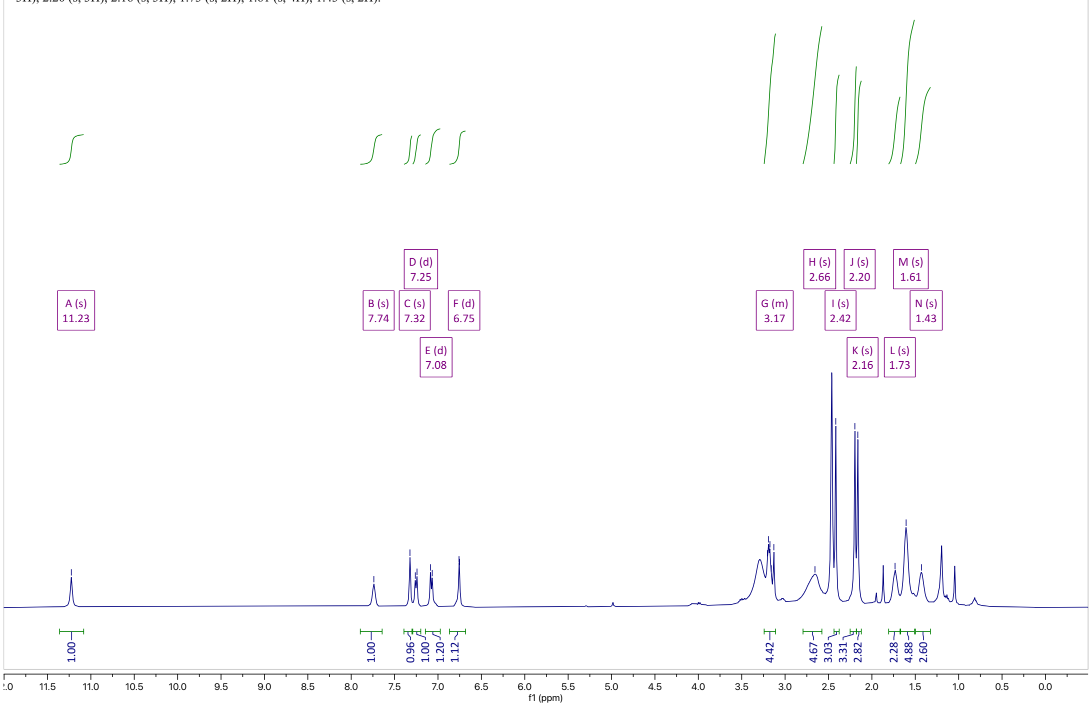

Compound 41

^{13}C NMR (101 MHz, DMSO-D_6) δ 165.21, 136.33, 133.60, 133.30, 129.96, 129.83, 128.85, 124.36, 120.62, 115.22, 103.85, 53.45, 36.43, 29.03, 27.43, 25.29, 23.92, 19.55, 18.99, 12.57.

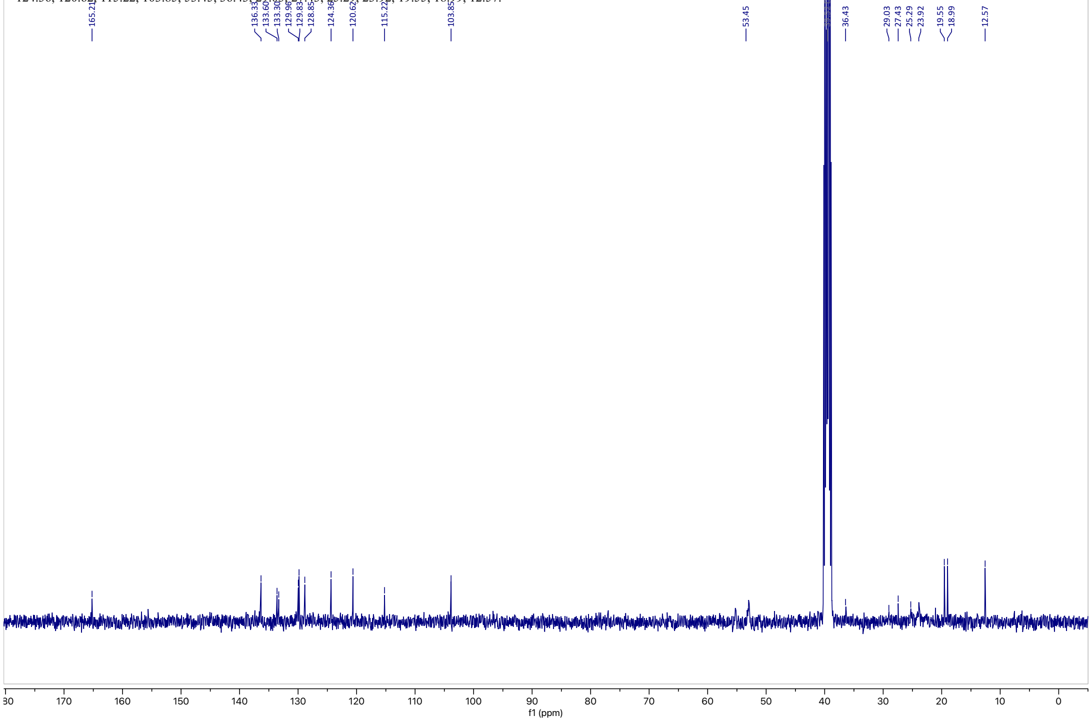

Compound 42

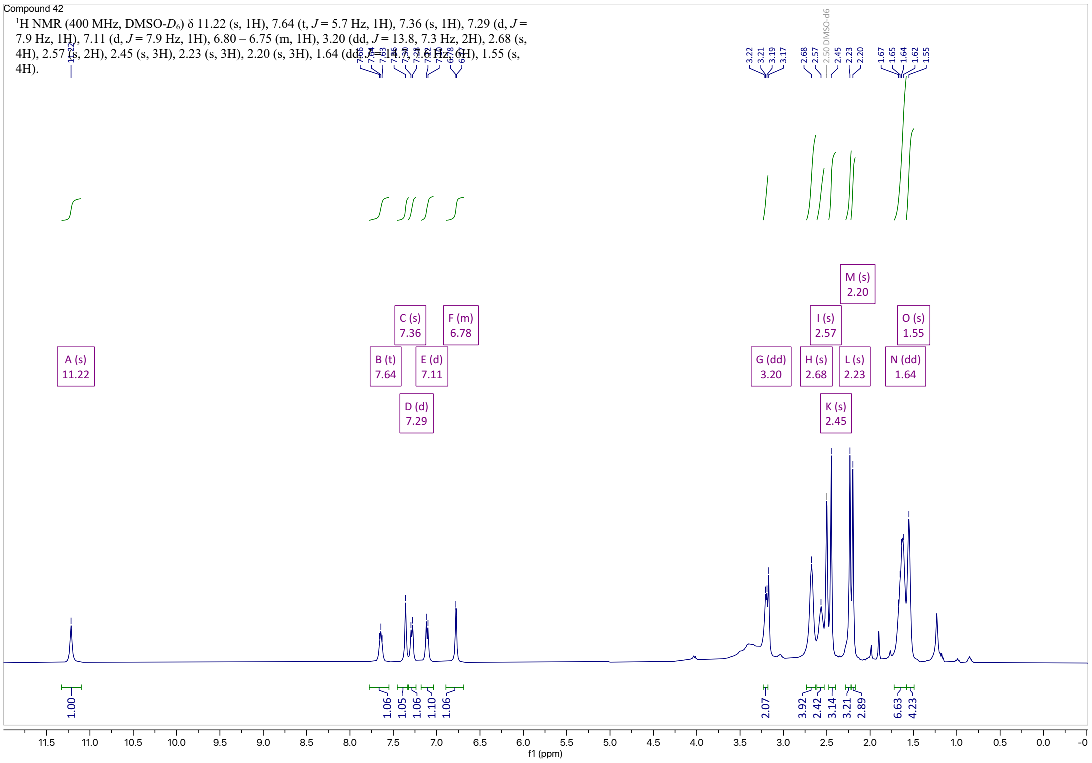
Compound 42
^{1}H NMR (400 MHz, DMSO-D_6) δ 11.22 (s, 1H), 7.64 (t, J = 5.7 Hz, 1H), 7.36 (s, 1H), 7.29 (d, J = 7.9 Hz, 1H), 7.11 (d, J = 7.9 Hz, 1H), 6.80 – 6.75 (m, 1H), 3.20 (dd, J = 13.8, 7.3 Hz, 2H), 2.68 (s, 4H), 2.57 (s, 2H), 2.45 (s, 3H), 2.23 (s, 3H), 2.20 (s, 3H), 1.64 (dd, J = 14.7, 7.6 Hz, 6H), 1.55 (s, 4H).
f1 (ppm)

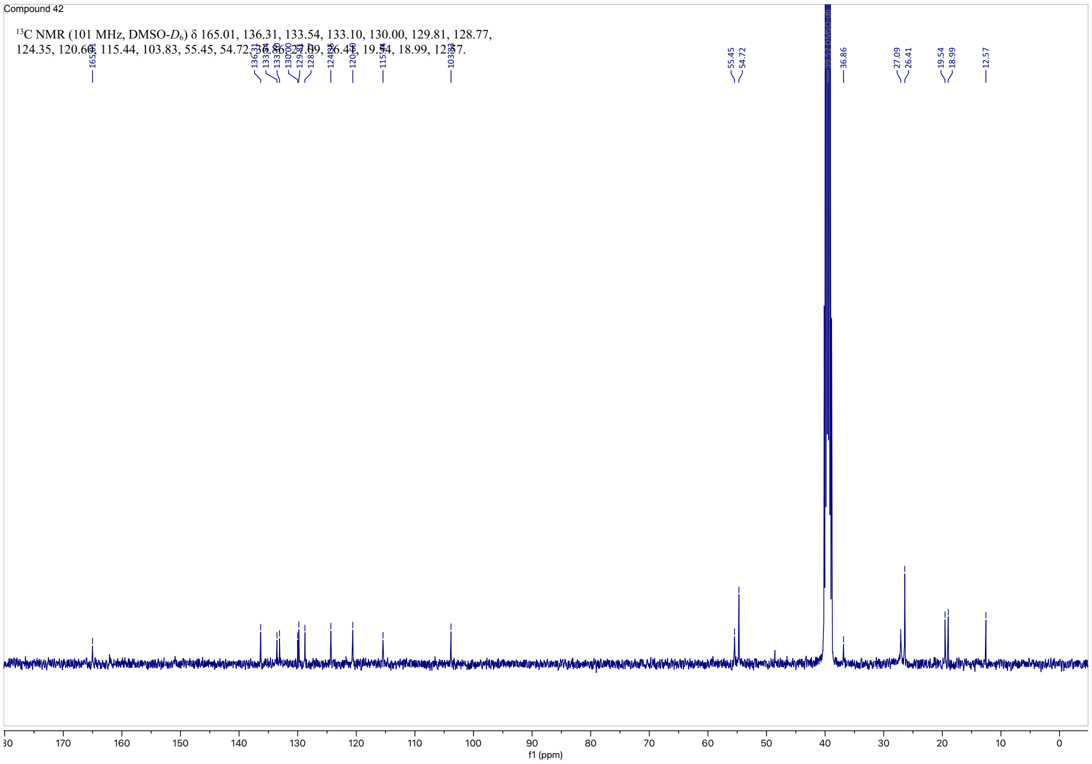
Compound 42
^{13}C NMR (101 MHz, DMSO-D_6) δ 165.01, 136.31, 133.54, 133.10, 130.00, 129.81, 128.77, 124.35, 120.60, 115.44, 103.83, 55.45, 54.72, 36.86, 27.09, 26.41, 19.54, 18.99, 12.57.
f1 (ppm)

Compound 43

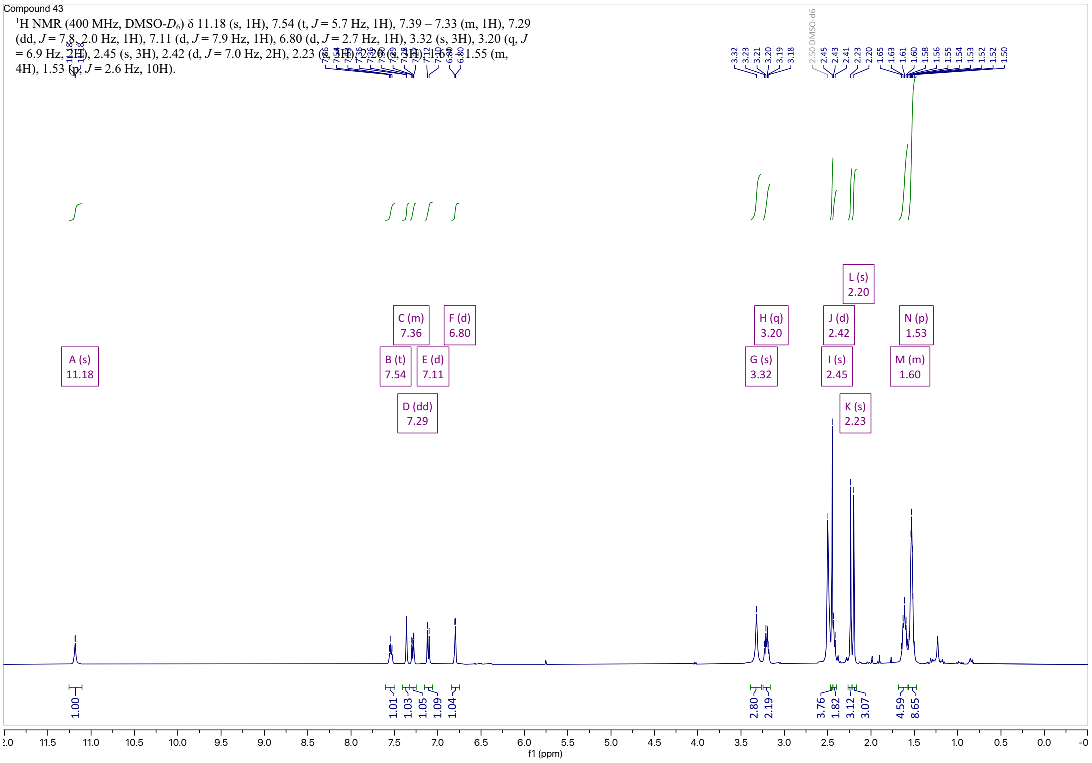
Compound 43
^{1}H NMR (400 MHz, DMSO-D_6) δ 11.18 (s, 1H), 7.54 (t, J = 5.7 Hz, 1H), 7.39 – 7.33 (m, 1H), 7.29 (dd, J = 7.8, 2.0 Hz, 1H), 7.11 (d, J = 7.9 Hz, 1H), 6.80 (d, J = 2.7 Hz, 1H), 3.32 (s, 3H), 3.20 (q, J = 6.9 Hz, 2H), 2.45 (s, 3H), 2.42 (d, J = 7.0 Hz, 2H), 2.23 (s, 3H), 2.20 (s, 3H), 1.67 – 1.55 (m, 4H), 1.53 (p, J = 2.6 Hz, 10H).
A (s) 11.18
B (t) 7.54
C (m) 7.36
D (dd) 7.29
E (d) 7.11
F (d) 6.80
G (s) 3.32
H (q) 3.20
I (s) 2.45
J (d) 2.42
K (s) 2.23
L (s) 2.20
M (m) 1.60
N (p) 1.53
2.50 DMSO-d6
f1 (ppm)

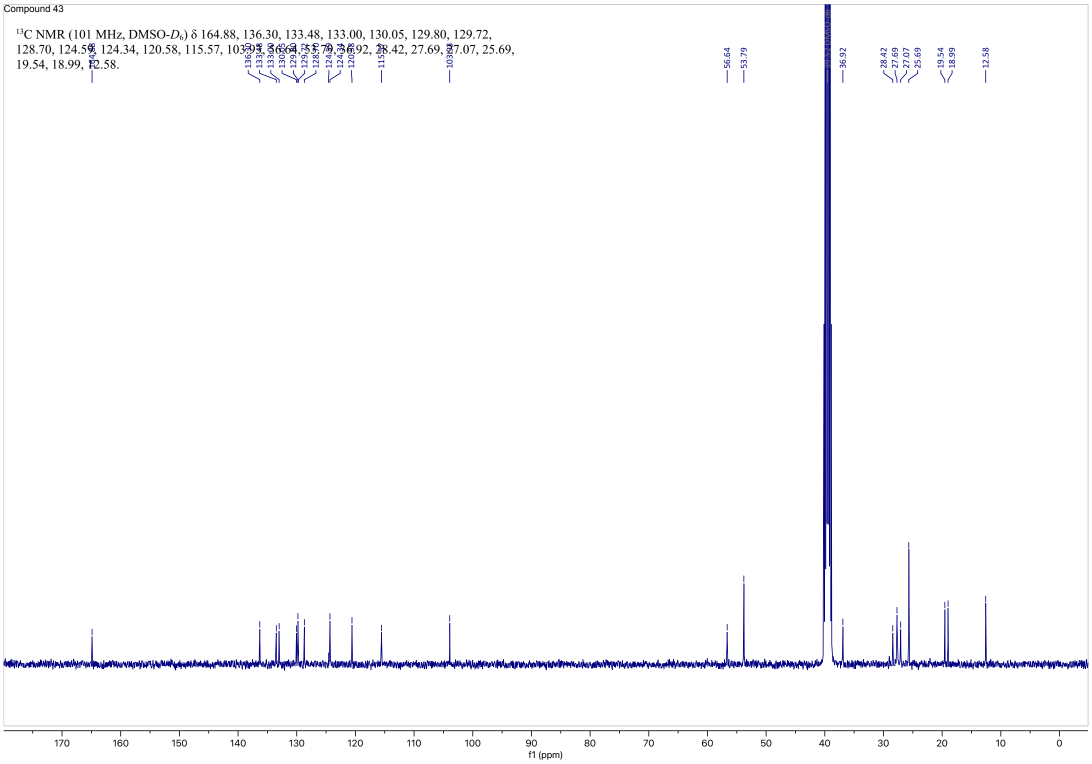
Compound 43
^{13}C NMR (101 MHz, DMSO-D_6) δ 164.88, 136.30, 133.48, 133.00, 130.05, 129.80, 129.72, 128.70, 124.59, 124.34, 120.58, 115.57, 103.93, 56.64, 53.79, 36.92, 28.42, 27.69, 27.07, 25.69, 19.54, 18.99, 12.58.
f1 (ppm)

Compound 44

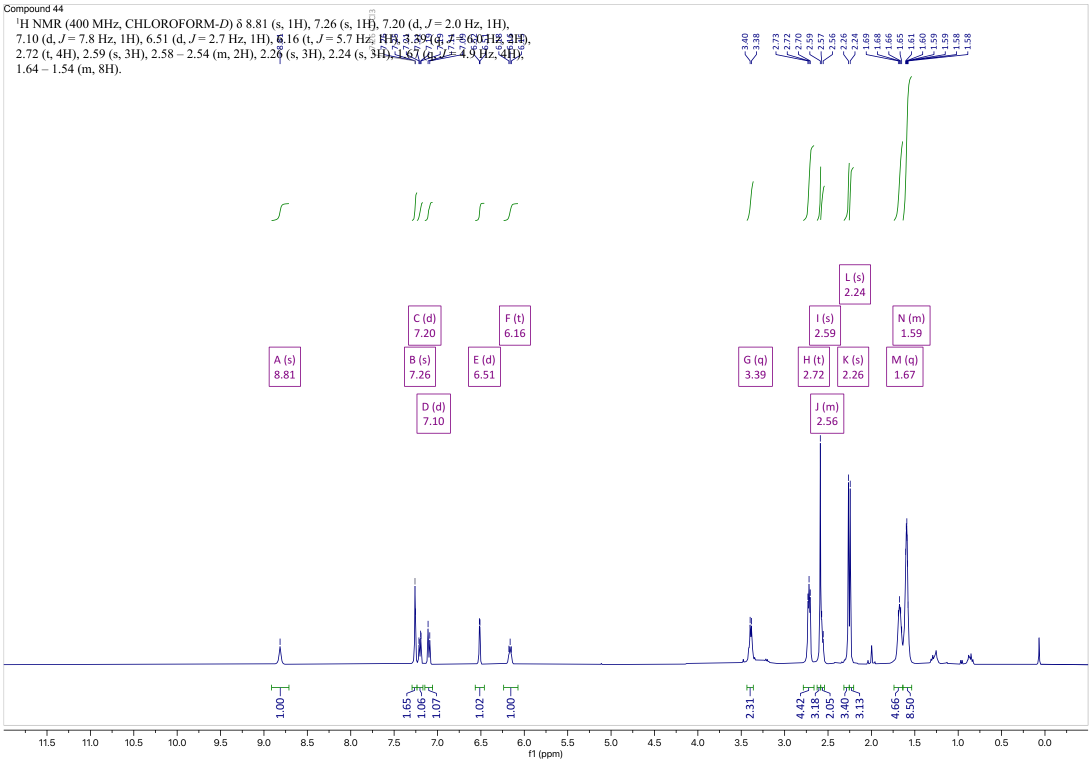
Compound 44
^{1}H NMR (400 MHz, CHLOROFORM-*D*) δ 8.81 (s, 1H), 7.26 (s, 1H), 7.20 (d, *J* = 2.0 Hz, 1H), 7.10 (d, *J* = 7.8 Hz, 1H), 6.51 (d, *J* = 2.7 Hz, 1H), 6.16 (t, *J* = 5.7 Hz, 1H), 3.39 (q, *J* = 6.0 Hz, 2H), 2.72 (t, 4H), 2.59 (s, 3H), 2.58 – 2.54 (m, 2H), 2.26 (s, 3H), 2.24 (s, 3H), 1.67 (q, *J* = 4.9 Hz, 4H), 1.64 – 1.54 (m, 8H).
f1 (ppm)

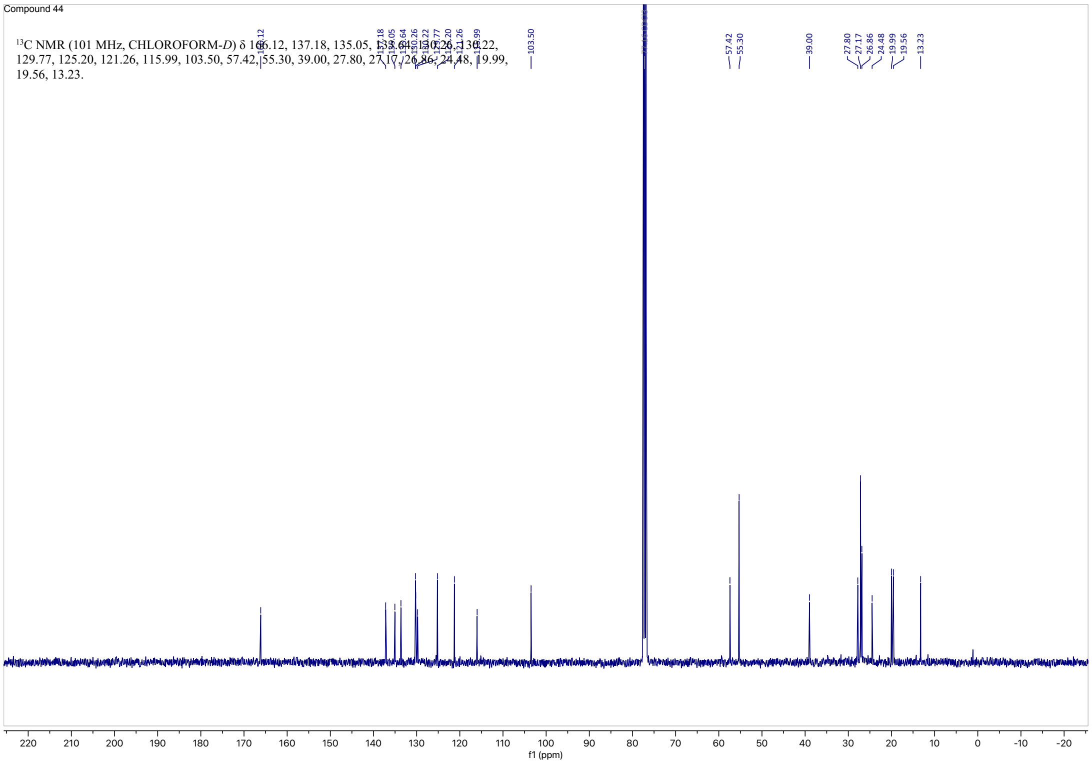
Compound 44
^{13}C NMR (101 MHz, CHLOROFORM-*D*) δ 166.12, 137.18, 135.05, 133.64, 130.26, 130.22, 129.77, 125.20, 121.26, 115.99, 103.50, 57.42, 55.30, 39.00, 27.80, 27.17, 26.86, 24.48, 19.99, 19.56, 13.23.
f1 (ppm)

Compound 45

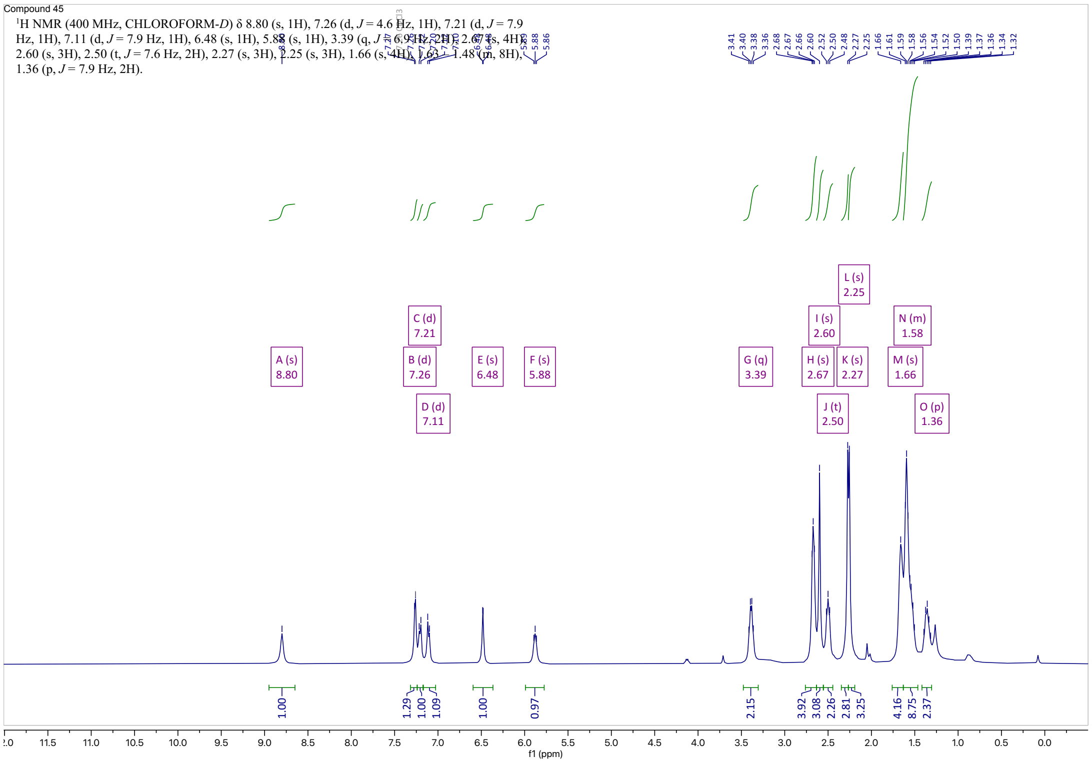
Compound 45
¹H NMR (400 MHz, CHLOROFORM-*D*) δ 8.80 (s, 1H), 7.26 (d, *J* = 4.6 Hz, 1H), 7.21 (d, *J* = 7.9 Hz, 1H), 7.11 (d, *J* = 7.9 Hz, 1H), 6.48 (s, 1H), 5.88 (s, 1H), 3.39 (q, *J* = 6.9 Hz, 2H), 2.67 (s, 4H), 2.60 (s, 3H), 2.50 (t, *J* = 7.6 Hz, 2H), 2.27 (s, 3H), 2.25 (s, 3H), 1.66 (s, 4H), 1.63 – 1.48 (m, 8H), 1.36 (p, *J* = 7.9 Hz, 2H).
f1 (ppm)

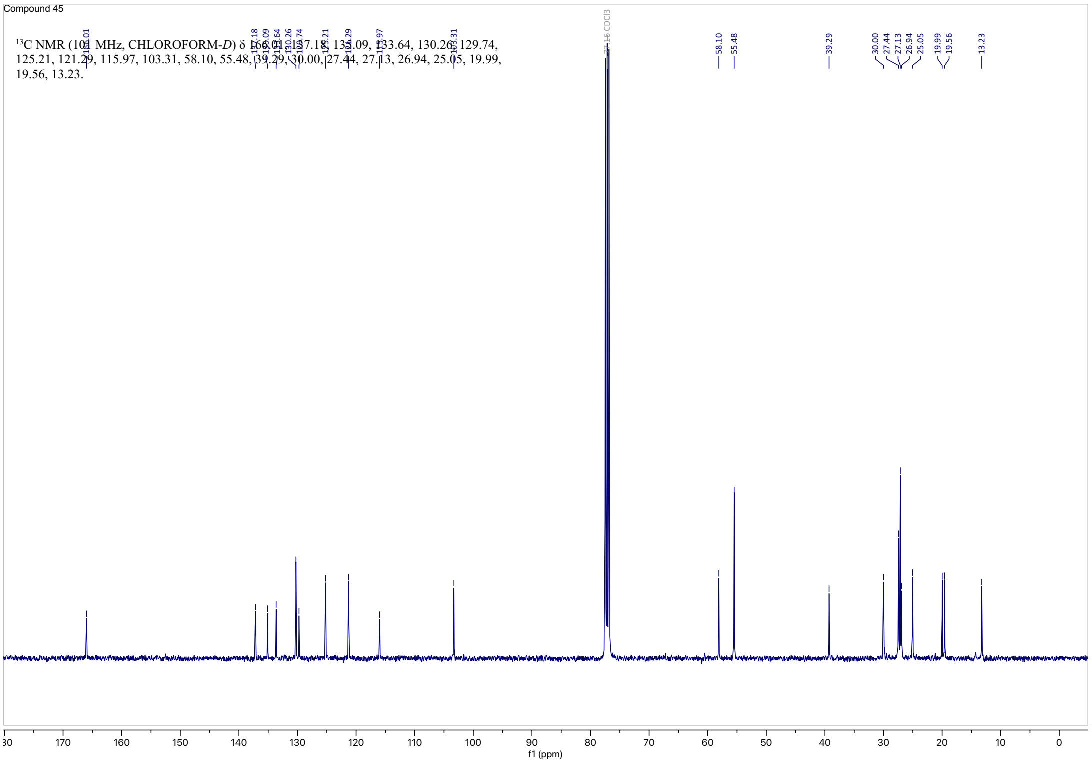
Compound 45
¹³C NMR (101 MHz, CHLOROFORM-*D*) δ 166.01, 137.18, 135.09, 133.64, 130.26, 129.74, 125.21, 121.29, 115.97, 103.31, 58.10, 55.48, 39.29, 30.00, 27.44, 27.13, 26.94, 25.05, 19.99, 19.56, 13.23.
77.16 CDCl3
f1 (ppm)
